# Indolo[2,3-*e*]benzazocines and indolo[2,3-*f*]benzazonines and their copper(ii) complexes as microtubule destabilizing agents†

**DOI:** 10.1039/d3dt01632c

**Published:** 2023-07-04

**Authors:** Christopher Wittmann, Orsolya Dömötör, Irina Kuznetcova, Gabriella Spengler, Jóhannes Reynisson, Lauren Holder, Gavin J. Miller, Eva A. Enyedy, Ruoli Bai, Ernest Hamel, Vladimir B. Arion

**Affiliations:** a Institute of Inorganic Chemistry, University of Vienna Währinger Strasse 42 A-1090 Vienna Austria vladimir.arion@univie.ac.at; b MTA-SZTE Lendület Functional Metal Complexes Research Group, Department of Inorganic and Analytical Chemistry, University of Szeged Dóm tér 7 H-6720 Szeged Hungary; c Department of Medical Microbiology, Albert Szent-Györgyi Health Center and Albert Szent-Györgyi Medical School, University of Szeged Semmelweis u. 6 H-6725 Szeged Hungary; d Schools of Chemical and Physical Sciences and Pharmacy and Bioengineering, Keele University Staffordshire ST5 5BG UK; e Molecular Pharmacology Branch, Developmental Therapeutics Program, Division of Cancer Diagnosis and Treatment, National Cancer Institute, Frederick National Laboratory for Cancer Research, National Institutes of Health Frederick Maryland 21702 USA

## Abstract

A series of four indolo[2,3-*e*]benzazocines HL^1^–HL^4^ and two indolo[2,3-*f*]benzazonines HL^5^ and HL^6^, as well as their respective copper(ii) complexes 1–6, were synthesized and characterized by ^1^H and ^13^C NMR spectroscopy, ESI mass spectrometry, single crystal X-ray diffraction (SC-XRD) and combustion analysis (C, H, N). SC-XRD studies of precursors Vd, VIa·0.5MeOH, of ligands HL^4^ and HL^6^·DCM, and complexes 2·2DMF, 5·2DMF, 5′·^i^PrOH·MeOH provided insights into the energetically favored conformations of eight- and nine-membered heterocycles in the four-ring systems. In addition, proton dissociation constants (p*K*_a_) of HL^1^, HL^2^ and HL^5^, complexes 1, 2 and 5, overall stability constants (log *β*) of 1, 2 and 5 in 30% (v/v) DMSO/H_2_O at 298 K, as well as thermodynamic solubility of HL^1^–HL^6^ and 1–6 in aqueous solution at pH 7.4 were determined by UV–vis spectroscopy. All compounds were tested for antiproliferative activity against Colo320, Colo205 and MCF-7 cell lines and showed IC_50_ values in the low micromolar to sub-micromolar concentration range, while some of them (HL^1^, HL^5^ and HL^6^, 1, 2 and 6) showed remarkable selectivity towards malignant cell lines. Ethidium bromide displacement studies provided evidence that DNA is not the primary target for these drugs. Rather, inhibition of tubulin assembly is likely the underlying mechanism responsible for their antiproliferative activity. Tubulin disassembly experiments showed that HL^1^ and 1 are effective microtubule destabilizing agents binding to the colchicine site. This was also confirmed by molecular modelling investigations. To the best of our knowledge, complex 1 is the first reported transition metal complex to effectively bind to the tubulin-colchicine pocket.

## Abstract 2

The tridentate ligand HL^1^ and its complex 1 showed good antiproliferative activity by inhibition of tubulin assembly.

## Graphical abstract

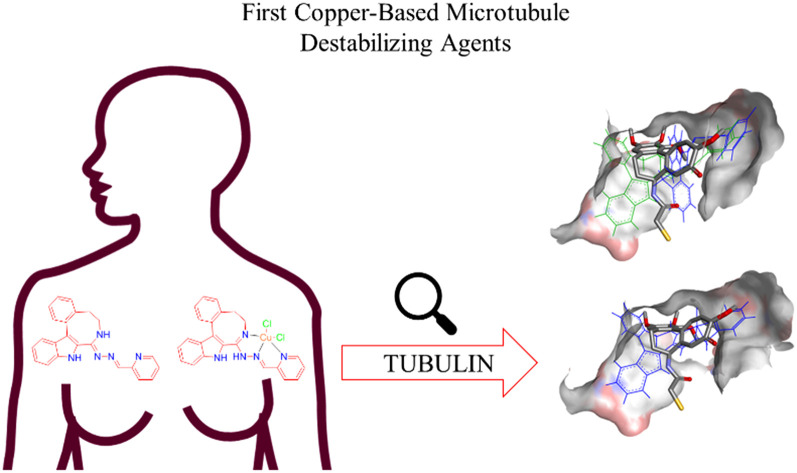

## 1 Introduction

**Chart 1:**
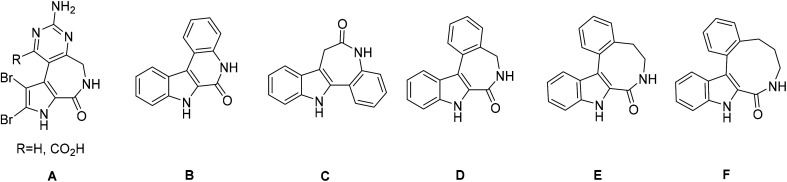
The frameworks of the naturally occurring latonduines (A) and of indolo[2,3-*c*]quinoline (B), indolo[3,2-*d*]benzazepine (paullone, C), indolo[2,3-*d*]benzazepine (D), indolo[2,3-*e*]benzazocine (E) and indolo[2,3-*f*]benzazonine (F).

Alkaloids, which are natural secondary metabolites found in certain plants, animals and fungi, have attracted remarkable attention in medicinal chemistry.^1^ Latonduines (structure A in Chart 1) derived from alkaloids extracted from marine sponge *Stylissa carteri*^2^ and other multiring systems, such as indolobenzazepines, exhibit good antiproliferative activity.^3^ All these fused heterocycles contain the indole moiety, which is also present in vinca alkaloids, and are potent inhibitors of tubulin polymerization.^4^ Strikingly, when this moiety is fused to organic scaffolds, the resulting molecules have been found to interfere with the mitotic spindle and thus inhibit cell proliferation, leading to apoptosis.^5–7^ Latonduines in their natural form are non-cytotoxic,^2^ but increased cytotoxicity and high affinity for microtubules occurred when the indole moiety was condensed to the main latonduine core.^8,9^ In contrast to latonduine derivatives and indolobenzazepines (see Chart 1, structures C and D), which have a bent structure, indoloquinolines (see Chart 1, structure B) also contain the indole moiety and are flat heterocyclic systems.^10,11^ Apart from antimalarial properties, indoloquinolines were found to exhibit antibacterial, antifungal, anti-inflammatory and anticancer effects.^10–13^ Indolobenzazepines, in contrast, have offered, so far, a far narrower spectrum of activities, and, in particular, good cytotoxicity effects towards cancer cells. Their mode of action seems to depend on the position of fusion of the indole moiety to a benzazepine ring, *i.e.* [3,2-*d*] or [2,3-*d*]. While paullones (indolo[3,2-*d*]benzazepines) are potent cyclin-dependent kinase (Cdk), mitochondrial malate dehydrogenase (MDH), sirtuin-1 (SIRT-1)- and glycogen-kinase synthase 3β (GSK3β) inhibitors,^14–18^ the isomeric indolo[2,3-*d*]benzazepines have been reported as effective microtubule targeting agents (MTA) binding in the colchicine site of the beta-subunit of tubulin.^8,9,19^ Indolo[2,3-*e*]benzazocine scaffolds (see Chart 1, structure E), which contain an eight-membered azocine ring instead of a seven-membered azepine ring, retain high cytotoxic activity and maintain the same mode of action, while further ring expansion to indolo[2,3-*f*]benzazonines (see Chart 1, structure F) was reported to result in reduced antiproliferative activity.^9^ Structural comparison of related four-ring systems, namely indolo[2,3-*c*]quinoline, isomeric indolo[3,2-*d*]benzazepine and indolo[2,3-*d*]benzazepine, as well as indolo[2,3-*e*]benzazocine and indolo[2,3-*f*]benzazonine, is shown in Chart 1.

It is worth noting that many indole-containing drugs are potent MTAs.^5–7^ Microtubules play important roles in the cell cycle and cell proliferation. They also are a major component of the cell cytoskeleton and are required for cell motility and vesicular transport.^5,20,21^ MTAs can be divided into microtubule-stabilizing agents (MSA) and microtubule-destabilizing agents (MDA). While MDAs hinder tubulin polymerization, MSAs inhibit depolymerization, both events eventually leading to apoptotic cell death.^21–25^ So far only a few Hg(ii), Au(i) and Ag(i) complexes have been reported as MTAs.^26–28^ Affinity for microtubules with microtubule destabilization was recently reported^29^ for functionalized indolobenzazepines and products of their reactions with Ru(ii)/Os(ii)–arene complexes. It should be noted that to the best of our knowledge, first-row transition metal complexes as MDAs or MTAs have not been reported.

**Chart 2:**
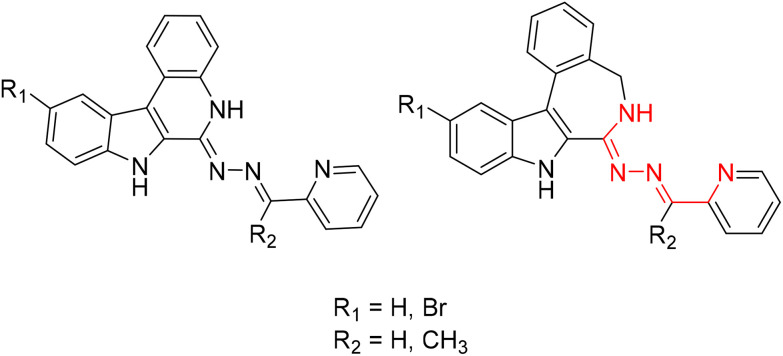
Structural relationship between previously reported indoloquinoline and indolobenzazepine tridentate ligands.

All the four-ring core systems shown in Chart 1 are not potentially chelating ligands, but they could be easily modified to provide a suitable binding site for a first-row transition metal. Previous research showed that indolobenzazepines and indoloquinolines had respectable antiproliferative activity.^9,30–34^ The potentially tridentate ligands (see Chart 2) allowed for the preparation of complexes with remarkable thermodynamic stability and that were resistant to hydrolysis.^30,33,34^

The chemical modifications of the proligands had little effect on the cytotoxicity and the mode of action of these compounds. However, upon complex formation the pharmacological profile can be enhanced, often resulting in increased cytotoxicity, as has been reported for indolo[2,3-*c*]quinolines and indolo[2,3-*d*]benzazepines.^30,31,34–36^ By taking advantage of the availability of four-ring systems incorporating eight-membered and nine-membered rings and our recently developed procedure for creation of a potentially tridentate binding site, we aimed at extending the variety of heterocyclic systems in a search for new biologically active ligands.^30,31^ Moreover, given the same type of structural changes in organic scaffolds compared to indolobenzazepines, new structure–activity relationships were envisioned.

Copper(ii) was chosen for complex formation, as complexes of this metal with this type of ligand usually exhibit an enhanced antiproliferative activity and pharmacological profile compared to their metal-free counterparts.^30–32,34,37^ It is worth noting that copper plays a pivotal role in cancer development. Due to enhanced cell growth and proliferation of malignant cells, the need for this essential trace metal is elevated in cancer tissues.^38–42^ In addition, Fenton-like reactions have been observed for the Cu(i)/Cu(ii) redox pair generating reactive oxygen species (ROS), which can also harm malignant cells.^39–41,43^

**Chart 3:**
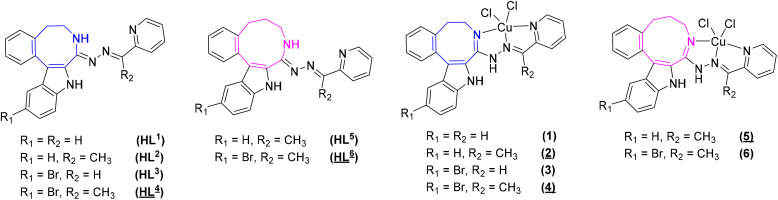
Indolo[2,3-*e*]benzazocine and indolo[2,3-*f*]benzazonine ligands and their respective copper(ii) complexes synthesized in this work; underlined numbers indicate compounds studied by SC-XRD.

Herein, we report the synthesis and characterization of six potential ligands HL^1^–HL^6^, four indolobenzazocines and two indolobenzazonines, and their respective copper(ii) complexes 1–6 (Chart 3). These new materials were comprehensively characterized by standard spectroscopic methods (^1^H NMR, 2D NMR and UV–vis), ESI mass spectrometry and X-ray crystallography. Their purity was confirmed by HPLC-HR-MS and elemental analysis. Furthermore, speciation in aqueous solution was conducted, their antiproliferative activity assessed, and the mode of action investigated.

## 2 Results and discussion

### 2.1 Synthesis and characterization of organic ligands

**Scheme 1:**
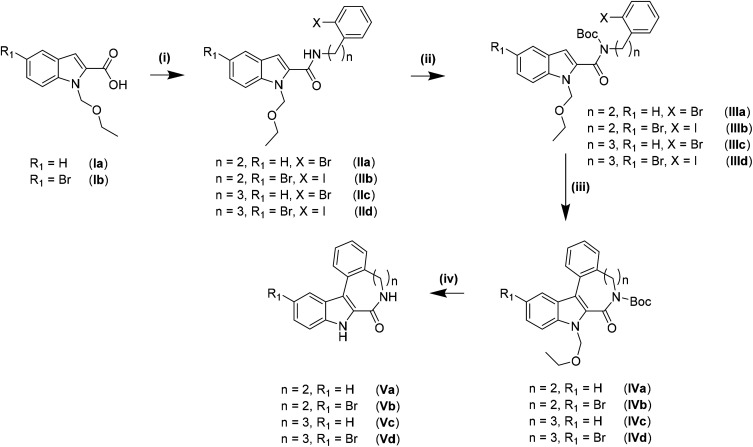
Synthesis of indolobenzazocines Va, Vb and indolobenzazonines Vc, Vd. Reagents and conditions: (i) 2-(2-bromophenyl/2-iodophenyl)ethanamine/3-(2-iodophenyl)propanamine, EDCI·HCl, DMAP, DCM_dry_, 0 °C – RT, 18 h; (ii) Boc_2_O, DMAP, MeCN_dry_, RT, 18 h; (iii) Pd(OAc)_2_, PPh_3_, Ag_2_CO_3_, DMF_dry_, 110 °C/140 °C, 2 h; (iv) HCl_conc_, dioxane, 80 °C, 2 h/EtOH : HCl_conc_ 4 : 1, 1 h.

The synthesis of the indolo[2,3-*e*]benzazocine and indolo[2,3-*f*]benzazonine cores was performed by adapting literature procedures.^9^ As can be seen from Scheme 1, different amines were used for the synthesis of indolobenzazocines Va and Vb. An amide coupling reaction starting from Ia/Ib and 2-(2-bromophenyl)ethan-1-amine or 2-(2-iodophenyl)ethan-1-amine afforded IIa/IIb, respectively, in almost quantitative yields (>90%). Boc protection of the amide nitrogen in IIa/IIb resulted in isolation of IIIa/IIIb with similar yields. The Pd-mediated cyclization of Br-substituted derivative IIIa into IVa was slightly less efficient than the cyclization of IIIa analogue with *n* = 2, R_1_ = H, X = I. However, the cyclization of the IIIb analogue with *n* = 2, R_1_ = Br, X = Br into IVb was realized in low yield and accompanied by the formation of several side products. A likely reason for the formation of side products might be intermolecular interactions due to similar reactivity of the brominated sites. In contrast, cyclization of the iodo precursor IIIb into IVb occurred cleanly without formation of any side products. Only a small amount of unreacted starting material was identified. Deprotection of IVa and IVb to give Va and Vb, respectively, was conducted in dioxane/1 M HCl at 80 °C, and pure products were isolated following recrystallization from methanol.

The amide coupling of 3-(2-iodophenyl)propanamine with carboxylic acids Ia and Ib resulted in high purity products IIc and IId with lower isolated yields compared to IIa and IIb. Boc-protection occurred smoothly and delivered high yields of IIIc and IIId as was the case for IIIa and IIIb. Cyclization of IIIc and IIId into indolobenzazonines IVc and IVd could be accomplished under harsher conditions (heating at 140 °C for at least 2 h) than for indolobenzazocines IVa and IVb. The products were isolated in moderate yields after column chromatography, while some starting materials were also recovered. Deprotection of IVc was conducted in dioxane/1 M HCl at 80 °C to give Vc in moderate yield after column chromatography, since deprotection was incomplete under these conditions. Only Boc-deprotection of IVd occurred under the same conditions with isolation of the ethoxymethyl (EOM)-protected derivative. Therefore, increasing the acidity of the deprotection conditions to EtOH : HCl_conc_ 4 : 1 at 100 °C was attempted.

**Scheme 2:**
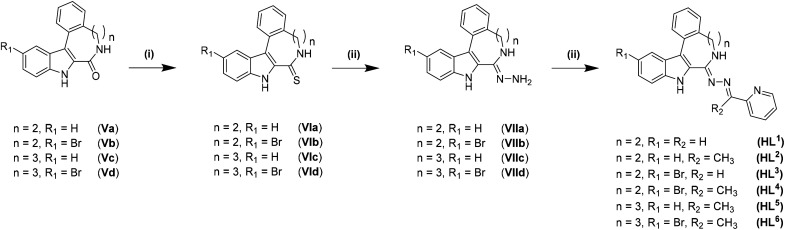
Synthesis of ligands HL^1^–HL^6^ derived from indolo[2,3-*e*]benzazocines and indolo[2,3-*f*]benzazonines. Reagents and conditions: (i) Lawesson's reagent, 1,4-dioxane_dry_, 4 h, 115 °C; (ii) H_2_N-NH_2_·H_2_O, CHCl_3_, reflux, 3 h; (iii) 2-acetylpyridine/2-formylpyridine, anoxic EtOH, 85 °C, 16 h.

Even though the literature suggested the opening of the lactam moiety by increasing the concentration of HCl,^9^ shortening the reaction time initially from 4 to 1 h resulted in a mixture of Vd, EOM-protected species and the derivative with an opened lactam. After basic workup and resuspension in acetone, Vd and its EOM-protected derivative were isolated as a mixture. Single crystals of X-ray diffraction quality were obtained from acetone and revealed the presence of both species in the asymmetric unit (Scheme 2).

For the synthesis of the ligands starting from reported species Va and Vc^9^ and novel compounds Vb and Vd established protocols^30,31,44^ had to be adapted (*vide infra*).

Thionation of the carbonyl function in Va–Vd with P_4_S_10_/Al_2_O_3_ did not result in thiolactams VIa–VId. However, by using Lawesson's reagent in dry dioxane, pure compounds were obtained in moderate to good yields (40–70%) after stirring at 115 °C under an argon atmosphere for 4 h, followed by workup and column chromatography. Moreover, the EOM-protected species could be easily separated from the deprotected product VId. Substitution of sulfur with hydrazine was achieved in chloroform when excess hydrazine monohydrate was used. Quantitative yields of clean products were obtained after reflux for up to 3 h. The use of neat hydrazine monohydrate for this transformation failed, resulting in unidentified products independently of reaction time. Schiff-base condensation, however, was performed under the same conditions as reported previously for indolobenzazepine systems.^29–31,45^

For ligands HL^1^–HL^6^, only one tautomeric form was evident in solution, according to ^1^H NMR spectra (for NMR atom numbering see Scheme S1 in the ESI†). Analysis of 2D NMR spectra indicated that the typical triplet in the region of 7.80–7.85 ppm can be attributed to protons H^7^ and H^8^. Furthermore, protons attached to aliphatic carbons C^5^ and C^6^ in indolobenzazocines show a peak splitting pattern between 3 and 4 ppm, providing further evidence for only one tautomeric species in solution. Differences between the brominated and unsubstituted species can only be elucidated from the total number of protons, since H^12^ and H^13^, respectively, are within resonances of aromatic protons overlapping with them. Distinct differences can be observed between HL^1^ and HL^2^, and HL^3^ and HL^4^, respectively. Chemical shifts of H^16^ and H^23^ are at 8.3 and 2.4 ppm, respectively. ^13^C NMR spectra of HL^2^ and HL^4^ show an additional peak at *ca.* 13 ppm. For indolobenzazonine derived ligands HL^5^ and HL^6^, only one methylene carbon from the heterocyclic ring system was present in the ^13^C NMR spectra. However, ^1^H NMR spectra indicated the identity of the protons attached to this carbon atom (shifts at ∼1.7 ppm, ∼2.5 ppm and ∼3 ppm, respectively). No correlation in the HMBC NMR spectra could be detected, even though X-ray diffraction structures (*vide infra*) confirmed the presence of these aliphatic carbons. Typical ^1^H and ^13^C shifts of H^24^ and C^24^ for HL^6^ were in the same region as for H^23^ and C^23^ resonances of HL^2^ and HL^4^, respectively.

The purity of HL^1^–HL^4^ (>95%) was confirmed by elemental analysis. This was further supported by ^1^H and ^13^C NMR spectra, which were measured after drying the samples at 50 °C *in vacuo*. A typical singlet at 5.75 ppm and resonance signal at 54.84 ppm in the ^1^H and ^13^C NMR spectra, respectively, could serve as diagnostic signals. The amounts of residual solvent estimated from ^1^H NMR spectra were in good agreement with elemental analyses of the complexes. HPLC-HR-MS and ESI-MS measurements in MeCN/H_2_O provided further evidence for the formation of ligands HL^1^–HL^6^ and their purity. Peaks at *m*/*z* 366.1732, 380.1872, 444.0827, 458.0989, 394.20 and 474.17 for HL^1^–HL^6^, respectively, were attributed to [M + H]^+^ species for each ligand.

### 2.2 Synthesis and characterization of 1–6

Copper(ii) complexes 1–6 were synthesized by reaction of CuCl_2_·2H_2_O with the corresponding ligand in alcohol on heating with yields ranging from 65% (2) to 92% (3) (see Experimental). HPLC-HR-MS characterization of complexes 1–4 in MeCN/MeOH and ESI mass spectra of 5 and 6 confirmed their formation. The peaks with *m*/*z* 427.08, 441.10, 506.99, 521.01, 491.16 and 571.01 for 1–6 were attributed [M − HCl − Cl]^+^ (1–4) and [M − Cl]^+^ (5 and 6). The isotopic distributions were in very good agreement with calculated isotopic patterns. However, complexes 1–4 were at least in part dissociated upon HPLC-HR-MS measurements, as indicated by the appearance of peaks with *m*/*z* attributable to the free ligand(s). The purity (>95%) required for biological assays was confirmed by elemental analysis. The thermodynamic stability and kinetic inertness of copper(ii) complexes will be discussed in more detail in section 2.4 (*vide infra*).

### 2.3 X-ray crystallography

**Fig. 1:**
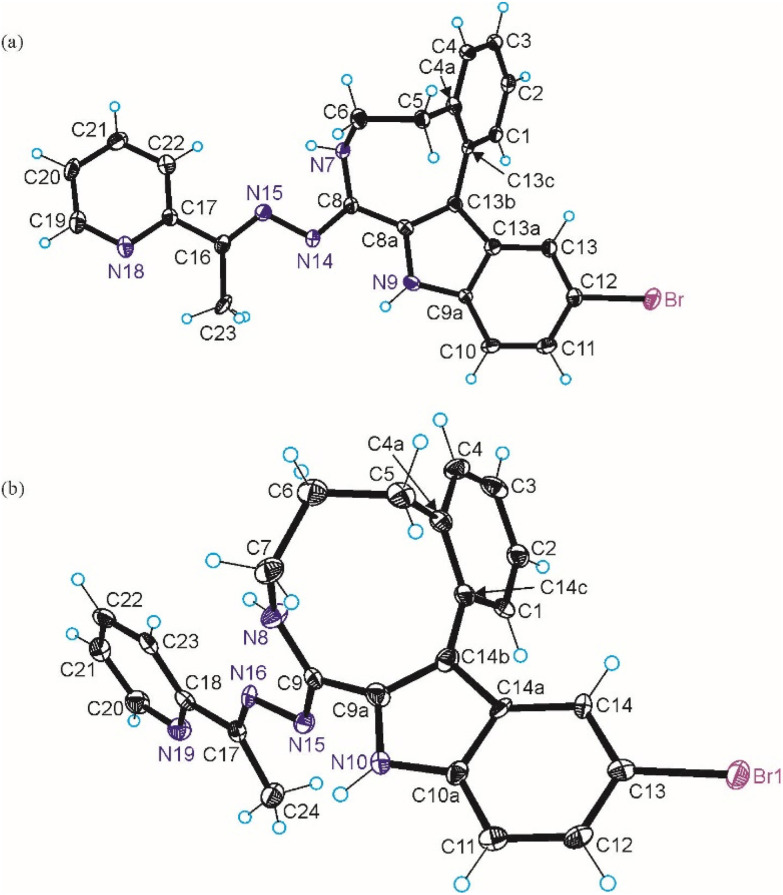
ORTEP views of (a) HL^4^ and (b) HL^6^ with thermal ellipsoids at 50% probability level.

**Fig. 2:**
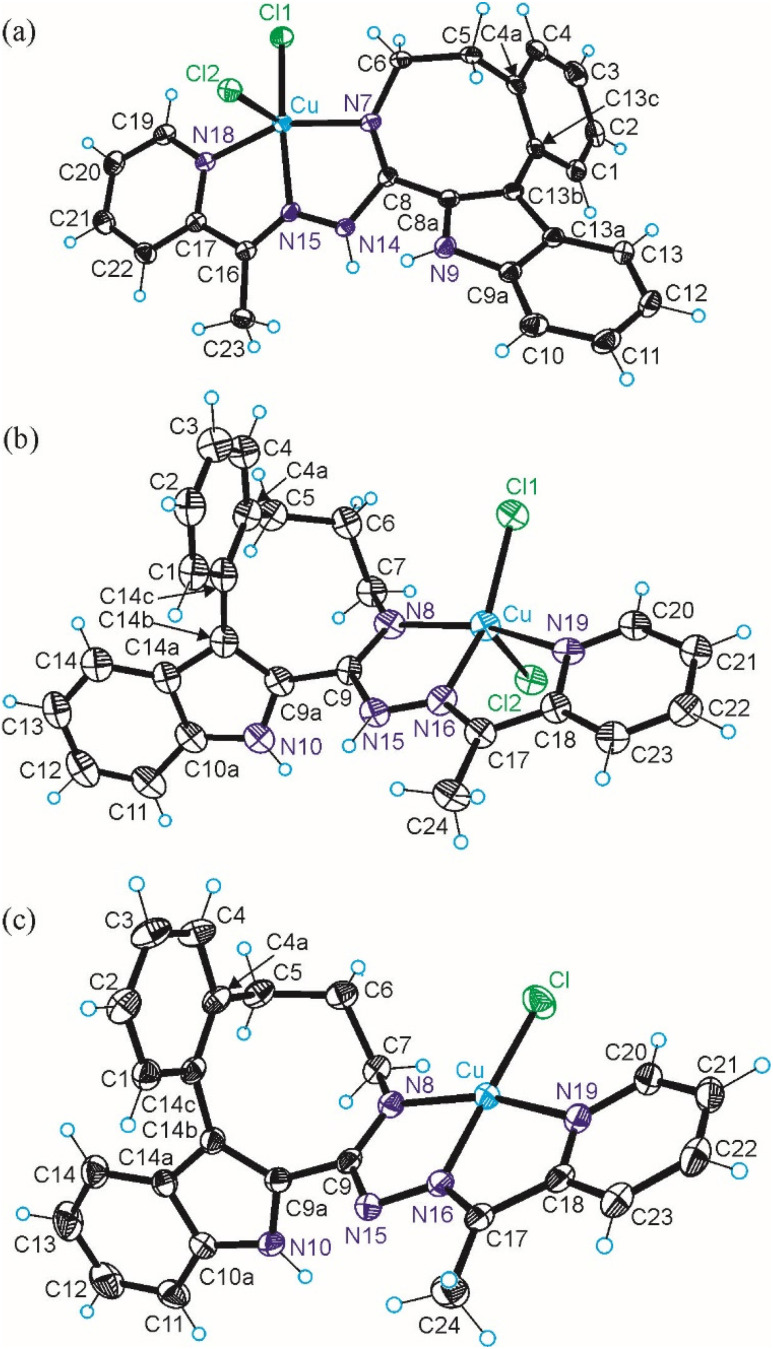
ORTEP views of the complexes: (a) 2 with thermal ellipsoids at 50% probability level, (b) 5 with thermal ellipsoids at 40% probability level and 5′ with thermal ellipsoids at 50% probability level. Selected bond distances (Å), bond angles (deg) and torsion angles (deg) (a) in 2: Cu–N7 2.0375(17), Cu–N15 1.9571(16), Cu–N18 2.0554(17), Cu–Cl1 2.4940(5), Cu–Cl2 2.2325(5), N7–C8 1.291(2), C8–N14 1.385(3), N14–N15 1.368(2), N15–C16 1.277(3), C16–C17 1.483(3), C17–N18 1.362(3); N7–Cu–N15 79.40(7), N15–Cu–N18 78.04(7), *Θ*_C4a–C5–C6–N7_ 66.3(3); *τ*_5_ = 0.13; (b) in 5: Cu–N8 1.999(5), Cu–N16 1.994(5), Cu–N19 2.044(5), Cu–Cl1 2.2684(17), Cu–Cl2 2.4002(16); N8–C9 1.308(7), C9–N15 1.395(7), N15–N16 1.331(7), N16–C17 1.300(7), C17–C18 1.458(8), C18–N19 1.363(8); N8–Cu–N16 78.8(2), N16–Cu–N19 78.47(19); *Θ*_C4a–C5–C6–C7_ −85.1(7), *Θ*_C5–C6–C7–N8_ 84.9(7); *τ*_5_ = 0.13; (c) in 5′: Cu–N8 1.9511(16), Cu–N16 1.9460(16), Cu–N19 2.0249(17), Cu–Cl 2.2006(6), N8–C9 1.319(3), C9–N15 1.357(2), N15–N16 1.365(2), N16–C17 1.297(3), C17–C18 1.475(3), C18–N19 1.359(3); N8–Cu–N16 80.23(7), N16–Cu–N19 80.39(7); *Θ*_C4a–C5–C6–C7_ −84.0(2), *Θ*_C5–C6–C7–N8_ 89.4(2).

**Fig. 3:**
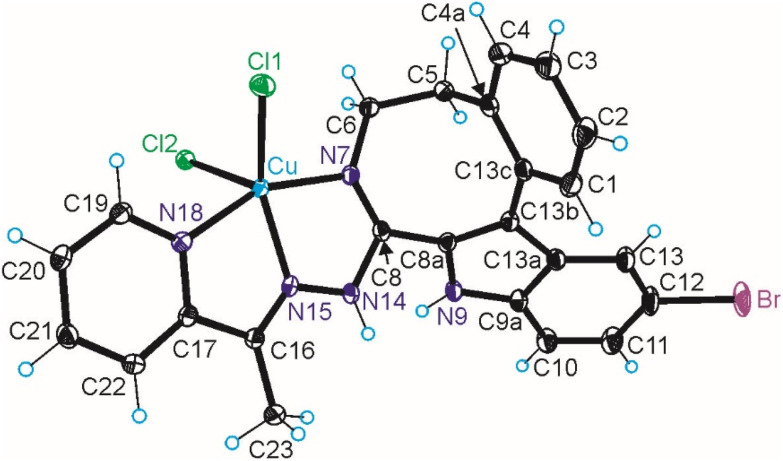
ORTEP view of the complex 3 with thermal ellipsoids at 50% probability level. Selected bond distances (Å), bond angles (deg) and torsion angles (deg): Cu–N7 2.0120(16), Cu–N15 1.9676(15), Cu–N18 2.0279(16), Cu–Cl1 2.4341(7), Cu–Cl2 2.2802(7); N7–C8 1.289(2), C8–N14 1.388(2), N14–N15 1.365(2), N15–C16 1.279(2), C16–C17 1.478(3), C17–N18 1.363(2); N7–Cu–N15 79.49(7), N15–Cu–N18 78.29(6), *Θ*_C4a–C5–C6–N7_ −48.6(2); *τ*_5_ = 0.17.

The results of SC-XRD studies of HL^4^ and HL^6^·DCM, are shown in Fig. 1, while those of 2·2DMF, 5·2DMF, 5′·^i^PrOH·MeOH and 3 in Fig. 2 and 3, respectively. The structures of precursors IIIa, Vd, VIa·0.5MeOH are presented in Fig. S1–S3 in the ESI.† Pertinent bond distances (Å), bond angles and torsion angles (deg) are quoted in the captions to the figures.

The benzazocine HL^4^ and benzazonine HL^6^·DCM crystallized in the orthorhombic space group *Pna*2_1_ and in the triclinic centrosymmetric space group *P*1̄, respectively, while the copper(ii) complexes 2·2DMF, 5·2DMF and 3·2^i^PrOH·H_2_O, as well as 5′·^i^PrOH·CH_3_OH in the triclinic space group *P*1̄, monoclinic centrosymmetric space groups *C*2/*c* and *P*2_1_/*c*, and in the triclinic space group *P*1̄, respectively.

The complexes 2, 3 and 5 are five-coordinate close to square-pyramidal^46^ with *τ*_5_ = 0.13, 0.13 and 0.17, respectively, while 5′ is square-planar. All ligands act as tridentate and either neutral (2, 3 and 5) or monoanionic (5′).

The coordination number (4 or 5) is achieved *via* binding of one or two chlorido co-ligands. Comparison of bond lengths in complex 2 with an eight-membered benzazocine ring-based ligand with those in 5 with a nine-membered benzazonine ring-based ligand revealed that three bonds in the coordination polyhedron of 2 (Cu–N7, Cu–N18 and Cu–Cl2) were markedly longer, while the other two bonds (Cu–N15 and Cu–Cl1) were significantly shorter than the corresponding bonds in 5, due to different steric flexibility of the two organic ligands. The coordination bonds in five-coordinate complex 5 are generally longer than those in square-planar complex 5′ due to stronger interatomic repulsions in the former compound in accord with VSEPR theory.

**Scheme 3:**
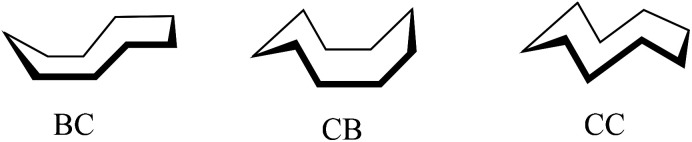
The three most stable conformers of cyclononane reported by Hendrickson.^47^

Of particular note is the conformation of the nine-membered azonine ring in HL^6^, precursor Vd and complexes 5 and 5′. The relevance of three idealized conformers of *C*_s_ local symmetry for cyclononane, namely the boat–chair (BC), the chair–boat (CB), and the chair–chair (CC) was reported (Scheme 3), while the boat–boat (BB) conformation was disregarded due to its very high energy.^47^

Calculations indicated that all three forms are located at the saddle points on the energy surface. However, when twisted (T) they reach energy minima corresponding to TBC, TCB and TCC conformers.^47,48^ Electron diffraction^49^ and low-temperature NMR data^50,51^ revealed that cyclononane exists in gas phase or in solution as a mixture of only two main conformers, namely TCB and TBC. Moreover, SC-XRD data revealed that cyclononanol-1-(dimethyl phosphonate) and 1,4-dioxaspiro[4.8]tridecane crystallized as TBC conformers,^52,53^ while the cyclononanone in the complex with HgCl_2_ adopted the TCB conformation.^54^

**Scheme 4:**
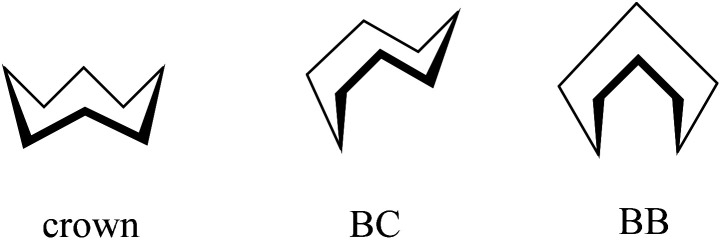
Main conformers of cyclooctane according to Hendrickson.^47^

For cyclooctane three main conformers, namely crown, BC and BB (Scheme 4) were first analyzed by Hendrickson.^47^

Cyclooctane was further studied by gas-phase electron diffraction in combination with NMR spectra and molecular mechanics calculations by various research groups to elucidate the most favored energetic conformation of this highly flexible structure. It was concluded that the BC conformer was the predominant and most stable species.^55–60^ More recent findings proved the existence of twisted forms, which show reduced strains and are energetically most favored.^47,61,62^

**Fig. 4:**
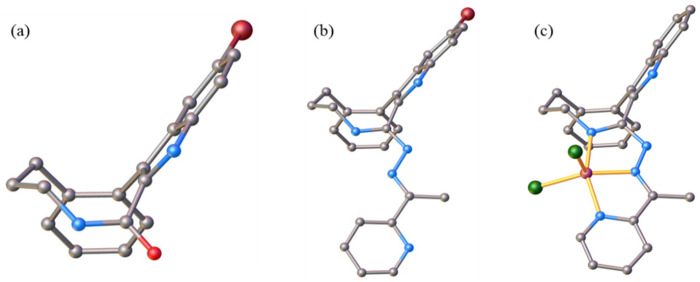
Conformation of the indolobenzazonine ring in Vd (a), HL^6^ (b) and 5 (c).

Inspection of indolobenzazonine derived structures Vd, HL^6^, 5 and 5′ indicated that the nine-membered ring in these compounds adopts the CB conformation (see Fig. 4), which is in good accordance with theoretical and experimental findings for substituted benzazonines,^63,64^ and also in agreement with the most favored CB conformation for substituted cyclononanes.^47,49^ The respective torsion angles are quoted in Table 1.

**Table 1:** Torsional angles in nine-membered azonine ring(s) in Vd·0.5MeOH, HL^6^, 5 and 5′

Torsional angles	Vd·0.5MeOH	HL^6^	5	5′
*Θ*_C7–C6–C5–C4a_	80.7(6)	80.8(6)	85.0(7)	84.0(2)
*Θ*_C6–C5–C4a–C14c_	−98.2(7)	−97.6(6)	−94.9(7)	−91.4(2)
*Θ*_C5–C4a–C14c–C14b_	9.4(9)	7.1(8)	1.9(9)	2.5(3)
*Θ*_C4a–C14c–C14b–C9a_	74.1(9)	76.2(7)	75.1(8)	82.6(3)
*Θ*_C14c–C14b–C9a–C9_	4.9(10)	4.2(9)	4.0(9)	−6.0(3)
*Θ*_C14b–C9a–C9–N8_	−77.2(8)	−72.8(8)	−73.4(8)	−68.9(3)
*Θ*_C9a–C9–N8–C7_	−15.2(9)	−22.0(8)	−15.7(9)	−12.8(3)
*Θ*_C9–N8–C7–C6_	103.4(7)	109.1(6)	103.8(7)	105.2(2)
*Θ*_N8–C7–C6–C5_	−80.2(6)	−79.8(6)	−84.9(7)	−89.4(2)

**Fig. 5:**
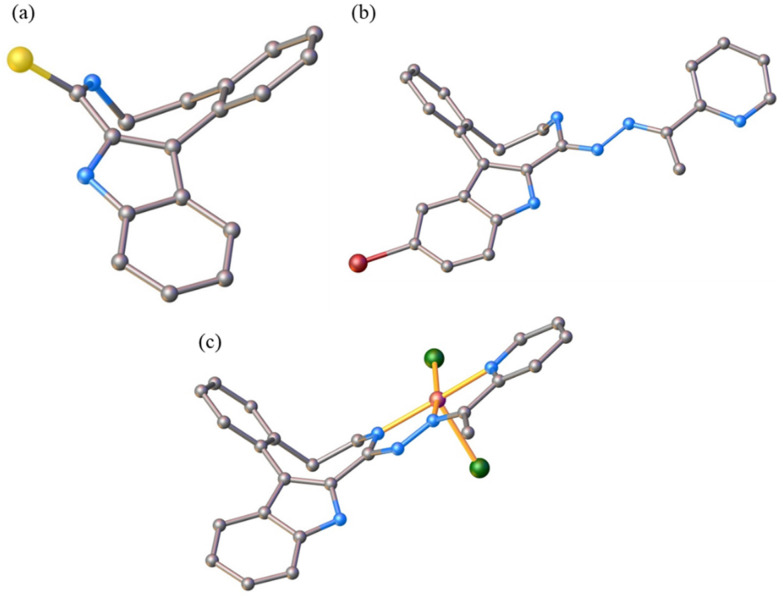
Conformation of the indolobenzazocine ring in VIa (a), HL^4^ (b) and 2 (c) with the indole moiety removed for better visualization.

In contrast to the cyclooctane with the most energetically favored BC conformation,^47,55^ the eight-membered ring in the benzazocine derivatives investigated here adopt the twisted boat conformation (see Fig. 5). The respective torsion angles are summarized in Table S1 in the ESI.†

One reason for the different least-energy conformers for indolobenzazocines (CB) and for cyclooctane (TBC) might be the distinct substitution patterns. Substituted cyclooctane derivatives have been shown to adopt conformers other than BC as energetically most favored.^65,66^

The conformations of 8- and 9-membered rings in the SC-XRD structures of Vd, VIa, HL^4^, HL^6^, 2, 4 and 5 are in agreement with those reported for other related compounds.^63,64,67,68^

### 2.4 Solution behavior: proton dissociation, stability and solubility

The thermodynamic solubility (*S*) of ligands HL^1^–HL^6^ and complexes 1–6 in aqueous solution at physiological pH was assessed by determination of the concentration of the saturated solutions at 298 K by UV–vis spectrophotometric analysis (Table 2).

**Table 2:** Thermodynamic solubility (*S*) of the ligands and their Cu(ii) complexes at pH 7.4 (20 mM HEPES) in aqueous solution {*T* = 298 K; *I* = 0.10 M (KCl)}

	*S*_7.4_/μM		*S*_7.4_/μM
HL^1^	8	1	2
HL^2^	9	2	4
HL^3^	<1	3	<1
HL^4^	<1	4	<1
HL^5^	<1	5	<1
HL^6^	<1	6	<1

The obtained *S*_7.4_ values indicated fairly limited solubility of both the ligands and complexes, in accord with their strong lipophilic character, especially for the bromo-substituted compounds. As a consequence, the proton dissociation processes of the selected ligands were monitored in 30% (v/v) DMSO/H_2_O at a low concentration (10 μM) *via* UV–vis titrations. In the case of indolobenzazocines with an eight-membered azocine ring, HL^1^ and HL^2^ were chosen, while, as an indolobenzazonine containing a nine-membered azonine ring, HL^5^ was selected for these studies.

**Fig. 6:**
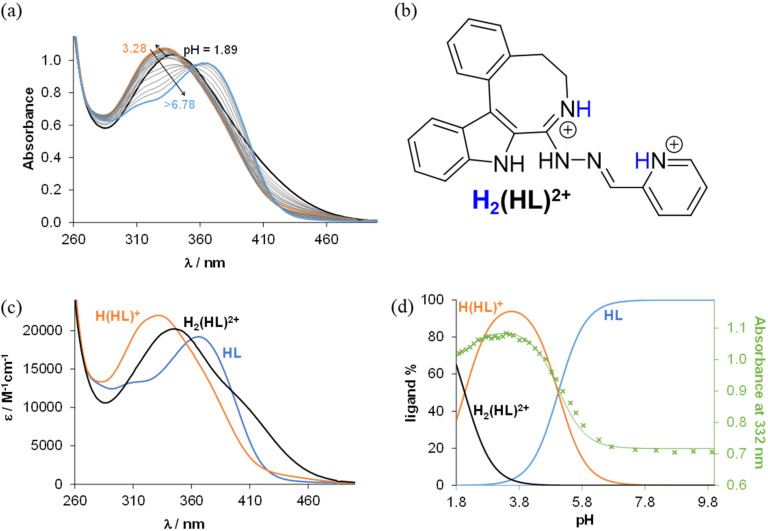
(a) UV–vis spectra of HL^1^ measured at various pH values. (b) The ligand in its diprotonated form. (c) Molar absorbance spectra computed for selected ligand species in the various protonation states. (d) Concentration distribution curves and the absorbance values measured at 332 nm (×) together with the fitted line {*c*_HL1_ = 10 μM, *T* = 298 K, *l* = 5 cm, *I* = 0.10 M (KCl), 30% (v/v) DMSO/H_2_O}.

**Scheme 5:**
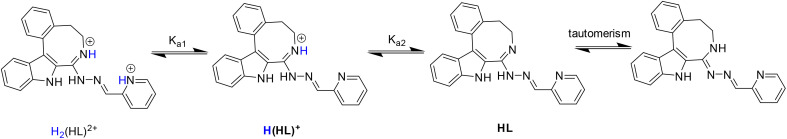
Stepwise proton dissociation processes for HL^1^ and its tautomeric forms.

From the pH-dependent spectral changes {Fig. 6a (for HL^1^) and Fig. S4a in the ESI† (for HL^5^)} two p*K*_a_ values could be determined (Table 3), as was also the case for the indolo[2,3-*d*]benzazepine (HL^1^_(7)_)^31^ and indolo[2,3-*c*]quinoline (HL^3^_(6)_)^30^ (subscript denotes the size of the central scaffold ring if not 8 or 9) reported recently. The first deprotonation step is assigned to the pyridinium nitrogen and accompanied by a blue shift (Fig. 6c and Fig. S4c in the ESI†), while the deprotonation of the benzazocinium (HL^1^) or benzazoninium (HL^5^) nitrogen results in a slight increase of the *λ*_max_ value and decrease of the absorbance (for the dissociation steps see Scheme 5). The methyl substituent increases the p*K*_a_ of the pyridinium nitrogen due to its electron donating effect (*cf.* data of HL^1^ and HL^2^) and does not affect the p*K*_a_ of the benzazepinium nitrogen. It is worth noting that the increase of the main ring size (*cf.* data of HL^2^ and HL^5^) results in somewhat lower p*K*_1_ and higher p*K*_2_ values. These p*K*_a_ values do not differ significantly from those of the structurally related indolo[2,3-*d*]benzazepine (see data for HL^1^_(7)_ in Table 3) and indolo[2,3-*c*]quinoline ligands with a six-membered pyridine-like ring.^22,25^ Based on the p*K*_a_ values, these compounds (and presumably also the bromo-derivatives) are present in their neutral form (HL) at pH 7.4. It is worth noting that the HL form can exist in two tautomeric forms as displayed in Scheme 5.

**Table 3:** Proton dissociation constants (p*K*_a_) of HL^1^, HL^2^, HL^5^ and HL^1^_(7__)_, overall stability constants (log *β*)^a^ and p*K*_a_ values of their Cu(ii) complexes determined by UV–vis titrations in 30 : 70% (v/v) DMSO/H_2_O solvent mixture {*T* = 298 K; *I* = 0.10 M (KCl)}

	HL^1^	HL^2^	HL^5^	HL^1^_(7)_ ^30^
p*K*_a1_ H_2_(HL)^2+^	2.07 ± 0.01	2.64 ± 0.01	2.41 ± 0.01	2.32 ± 0.01
p*K*_a2_ H(HL)^+^	5.04 ± 0.02	4.97 ± 0.02	5.55 ± 0.01	4.75 ± 0.01
log *β* [Cu(HL)]^2+^	8.55 ± 0.02	9.17 ± 0.02	9.57 ± 0.01	8.33 ± 0.01
log *β* [Cu(L)]^+^	4.14 ± 0.02	4.06 ± 0.02	4.31 ± 0.01	4.01 ± 0.01
p*K*_a_ [Cu(HL)]^2+^	4.41	5.11	5.26	4.32
p*K*_a_ [Cu(L)]^+^	>8	>8	>8	7.09
log *K*′ [Cu(L)]^+^ b	−2.97	−3.55	−3.65	−3.06

a The given error is originated from the fitting of the calculated and the experimental absorbance values in the wavelength range between 260 and 560 nm.

b *K*′ refers to the following equilibrium: H_2_(HL)^2+^ + Cu^2+^ ⇌ [Cu(L)]^+^ + 3H^+^.

**Fig. 7:**
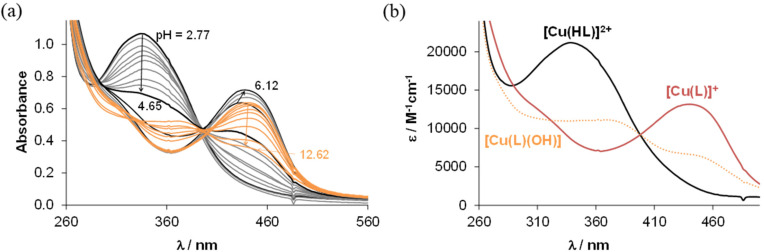
(a) UV–vis spectra for the Cu(ii)–HL^1^ system at various pH values. (b) Molar absorbance spectra computed for selected complex species in the various protonation states {*c*_HL1_ = 10 μM, *c*_Cu(II)_ = 10 μM, *T* = 298 K, *l* = 5 cm, *I* = 0.10 M (KCl), 30% (v/v) DMSO/H_2_O}.

To determine the overall stability constants (*β*) of the complexes formed in solution, UV–vis spectra for the Cu(ii) − HL^1^/HL^5^ systems at various pH values were measured (see Fig. 7 and Fig. S5 in the ESI†).

The decrease of the absorbance values due to the appearance of small amount of precipitate in the solution at pH > 7 hindered the accurate determination of the overall stability constants for the complexes formed in the basic pH range. As for indolo[2,3-*d*]benzazepine (HL^1^_(7)_),^30^ the formation of mono-ligand complexes [Cu(HL)]^2+^ and [Cu(L)]^+^ was found for indolo[2,3-*e*]benzazocine and indolo[2,3-*f*]benzazonine derivatives (Table 3). In these species the tridentate coordination *via* nitrogen atoms is suggested in solution. However, in the copper(ii) complex with the monoanionic ligand [Cu(L)]^+^, the deprotonation of the hydrazinic nitrogen not involved in coordination to copper(ii) was likely. The formation of this complex was accompanied by a significant red shift of the absorption band (Fig. 7b and Fig. S5b in the ESI†). This species was present at physiological pH together with a mixed hydroxido complex, *i.e.* [Cu(L)(OH)] (see p*K*_a_ values of the complexes in Table 3). The calculated overall stability constants indicated the formation of complexes with high thermodynamic stability in solution with absence or minor effect of the studied structural modifications on stability. Given the different basicities of the ligands, for comparison of the stability of the copper(ii) complexes with monoanionic ligands [Cu(L)]^+^, derived (*K*′) equilibrium constants were calculated (Table 3). Inspection of these values revealed that the presence of the methyl substituent somewhat decreased stability (*cf.*HL^1^ and HL^2^), whereas the size of the aza-containing ring had no impact on stability (*cf.*HL^1^ and HL^1^_(7)_, and HL^2^ and HL^5^). Comparison of the p*K*_a_ values of the complexes revealed that the methyl substituent increased the p*K*_a_ [Cu(HL)]^2+^, while the monoaza ring size had no effect on the pH range of the deprotonation of the complex.

### 2.5 *In vitro* cytotoxicity

The *in vitro* cytotoxic activity of the Cu(ii) complexes 1–6 and their respective ligands was investigated in the Colo205 and doxorubicin-resistant Colo320 human colon adenocarcinoma cell lines, MCF-7 breast cancer cells, and normal human embryonal lung fibroblast cells (MRC-5) by MTT assay, as detailed in the Experimental section. The IC_50_ values are shown in Table 4.

**Table 4:** IC_50_ values (μM) of the tested ligands and their Cu(ii) complexes^a^ on three human cancer cell lines (Colo205, Colo320, MCF-7) after a 72 h incubation, and in normal (MRC-5) cells. IC_50_ values of CuCl_2_ and doxorubicin (as positive control) are also shown for comparison

IC_50_/μM	Colo205	Colo320	MCF-7	MRC-5
HL^1^	30 ± 2	5.6 ± 0.1	1.3 ± 0.1	40 ± 1
1	2.5 ± 0.2	3.4 ± 0.1	6.2 ± 0.6	7.4 ± 0.3
HL^2^	2.2 ± 0.2	9.4 ± 0.4	1.2 ± 0.3	0.9 ± 0.1
2	0.62 ± 0.03	0.19 ± 0.02	0.94 ± 0.04	0.24 ± 0.01
HL^3^	29 ± 2	0.64 ± 0.04	0.44 ± 0.04	0.55 ± 0.04
3	4.4 ± 0.2	1.7 ± 0.2	2.5 ± 0.1	1.2 ± 0.1
HL^4^	54 ± 2	0.57 ± 0.02	0.60 ± 0.03	1.2 ± 0.1
4	1.7 ± 0.1	0.37 ± 0.02	0.73 ± 0.2	0.35 ± 0.04
HL^5^	0.06 ± 0.01	0.25 ± 0.02	0.54 ± 0.09	1.1 ± 0.1
5	0.08 ± 0.01	0.21 ± 0.02	2.2 ± 0.4	0.37 ± 0.02
HL^6^	0.11 ± 0.01	0.36 ± 0.01	0.11 ± 0.03	1.1 ± 0.2
6	0.16 ± 0.01	0.38 ± 0.01	0.69 ± 0.05	0.48 ± 0.04
CuCl_2_	33 ± 1	20 ± 2	32 ± 3	40 ± 1
Doxorubicin	0.82 ± 0.02	3.3 ± 0.1	0.21 ± 0.03	1.16 ± 0.11

a The presented results are the average of four replicates with IC_50_ and standard deviations.

Data in Table 4 show that the Cu(ii) complexes 1–4 are clearly more cytotoxic than their corresponding ligands in the Colo205 cells, while the IC_50_ values are mixed and closer for the complex–ligand pairs for the other two cancer cell lines. In general, the Colo320 and MCF-7 cells were somewhat more sensitive to the compounds than the Colo205 cells.

The data collected in Table 5 indicated remarkable selectivity of proligands HL^1^ for cancer cell lines Colo320 and MCF-7, HL^5^ for Colo205, and HL^6^ for Colo205 and MCF-7 with selectivity factors (SF) varying from 3.14 to 31.03. Among the copper(ii) complexes, the highest cell specific selectivity factors were achieved for 1, 5 and 6 against Colo205 cells with SF in the range from 3.03 to 4.78.

**Table 5:** Selectivity index (SI)^a^ is the ratio of the IC_50_ values measured on the normal cells and the cancer cells (based on the data listed in Table 2). SF (Colo205) = IC_50_ MRC-5/IC_50_ Colo205, SF (Colo320) = IC_50_ MRC-5/IC_50_ Colo320, SF (MCF-7) = IC_50_ MRC-5/IC_50_ MCF-7

	SF (Colo205)	SF (Colo320)	SF (MCF-7)
HL^1^	1.37	**7.26**	**31.03**
1	3.03	2.16	1.19
HL^2^	0.40	0.10	0.75
2	0.38	1.28	0.26
HL^3^	0.02	0.86	1.25
3	0.27	0.71	0.47
HL^4^	0.02	2.10	2.02
4	0.20	0.95	0.48
HL^5^	**17.6**	4.27	1.98
5	4.78	1.83	0.17
HL^6^	**10.1**	3.14	**10.18**
6	3.03	1.27	0.69

a Strongly selective compounds (SI > 6)^69^ are highlighted.

The bromo-substituent on the indole ring did not significantly affect the cytotoxicity of the complexes, unlike the methylation that enhanced markedly the cytotoxicity, similar to data described for related morpholine-indolo[2,3-*c*]quinoline and latonduine derivatives.^30,31^ It should be noted that the IC_50_ values of all Cu(ii) complexes in the tested human cancer cell lines fall into the low micromolar concentration range. It was reported previously that the indolo[2,3-*d*]benzazepine derivatives are generally less cytotoxic than the analogous indolo[2,3-*c*]quinoline compounds,^30^ most probably due to the stronger DNA binding ability of the complexes with the flat indoloquinoline ring system in comparison to the folded indolo[2,3-*d*]benzazepine. The compounds studied here with the eight-membered benzazocine ring were found to be even weaker DNA binders than the seven- or six membered analogs (*vide infra*), which might be responsible for their decreased cytotoxicity compared to the corresponding benzazepine and quinoline derivatives.

Submicromolar to nanomolar IC_50_ values in combination with some selectivity of several compounds studied for cancer cells prompted us to gain an insight into their mode of action. As the complexes reported previously showed ability to intercalate into DNA, we monitored their interaction with this macromolecule for comparative purposes. We also performed various tests to find out whether there was a difference in the ability to bind to DNA between metal-free ligands HL^1^–HL^6^ and Cu(ii) complexes 1–6.

### 2.6 Interaction with ct-DNA

**Fig. 8:**
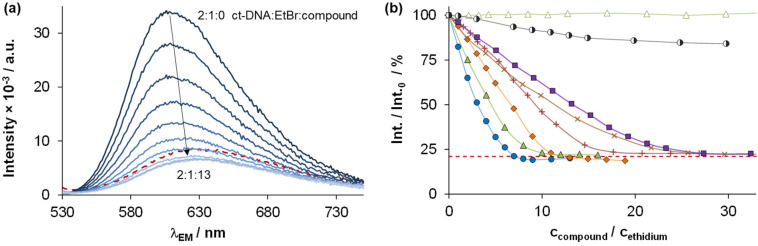
(a) Fluorescence emission spectra of the ct-DNA–ethidium system in the presence of an increasing amount of 4_(6)_, with the spectrum of free ethidium (red dashed line) plotted as well. (b) Fluorescence intensity values of the ct-DNA–ethidium in the presence of different compounds, symbols denote 4_(6)_ (●), 4_(7)_ (◆), 1 (▲), 2 (×), 4 (+), 5 (◑), 6 (■), and HL^1^ (Δ), red dashed line denotes the emission signal of free ethidium. At the indicated ratios, precipitate formation was not observed {*c*_DNA_ = 0.5 μM, *c*_ethidium_ = 0.25 μM; *λ*_EX_ = 510 nm, *λ*_EM_ = 610 nm; *T* = 298 K; 10 mM HEPES, pH = 7.40}.

The DNA binding of complexes (1, 2, 4–6) and ligands (HL^1^, HL^2^, HL^5^, HL^6^) was investigated in ethidium (cationic dye used as bromide salt) displacement studies by spectrofluorimetry. The complexes 4_(6)_ and 4_(7)_ were also studied as the indolo[2,3-*c*]quinoline and indolo[2,3-*d*]benzazepine analogues of 4, respectively, for comparison. Measurements were performed at highly diluted conditions (0.5 μM ct-DNA and 0.25 μM ethidium) to avoid precipitate formation. Fig. 8a shows the fluorescence spectrum of the ct-DNA–intercalated ethidium system. The intensity decreased gradually by the addition of complex 4_(6)_, and the final spectrum recorded at the highest excess of the complex was similar to that of free ethidium (dashed red spectrum).

**Fig. 9:**
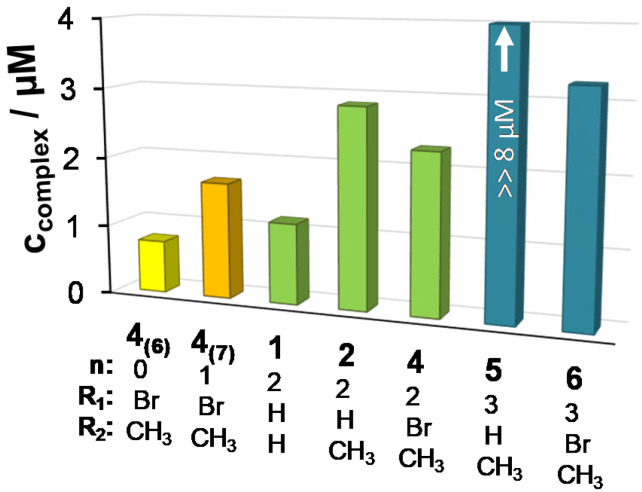
The complex concentration needed to result in 50% decrease in the fluorescence intensity of the ct-DNA–ethidium system. {*c*_DNA_ = 0.5 μM, *c*_ethidium_ = 0.25 μM; *λ*_EX_ = 510 nm, *λ*_EM_ = 610 nm; *T* = 298 K; 10 mM HEPES, pH = 7.40}.

A similar tendency was found for the Cu(ii) complexes 4_(7)_, 1, 2, 4 and 6 (Fig. 8b), the titration curves saturated to the intensity of the free ethidium ion. This finding strongly supports, that virtual displacement of intercalated ethidium took place (and not only quenching of the fluorescence of bound ethidium occurred). This means that these metal complexes displaced ethidium completely from ct-DNA. Complex 5, however, could only slightly displace ethidium, and, moreover, the ligands barely affected the fluorescence of the system (Fig. 9). The capability of replacing intercalated ethidium varied significantly among the complexes. Fig. 9 shows which concentrations of complexes were needed to reduce fluorescence in the ct-DNA–ethidium system by 50%. This efficacy followed the order 4_(6)_ > 1 > 4_(7)_ > 4 > 2 > 6 ≫ 5. Among the complexes with the eight-membered benzazocine ring, the non-substituted derivative 1 possessed the strongest ability to displace the ethidium cation. Decreasing the ring size clearly increased the binding of the complexes to ct-DNA.

**Fig. 10:**
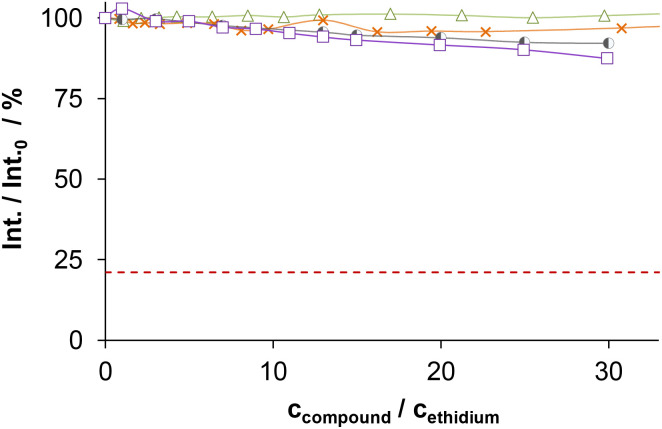
Fluorescence intensity values of the ct-DNA–ethidium of various ligand systems, symbols denote HL^1^ (Δ), HL^2^ (×), HL^5^ (◐) and HL^6^ (□), while red dashed line denotes the emission signal of free ethidium. At the indicated ratios precipitate formation was not observed. {*c*_DNA_ = 0.5 μM, *c*_ethidium_ = 0.25 μM; *λ*_EX_ = 510 nm, *λ*_EM_ = 610 nm; *T* = 298 K; 10 mM HEPES, pH = 7.40}.

On the one hand, 4_(6)_ displayed comparable affinity towards ct-DNA as its morpholine-substituted derivative HL^4^_(6)_.^30^ On the other hand, the ligands HL^1^, HL^2^, HL^5^ and HL^6^, similarly to their formerly investigated morpholine-functionalized derivatives,^30^ could not displace the intercalated ethidium cation (Fig. 10). It should also be noted that the apparent solubility of all the studied complexes was higher in the presence of ct-DNA in comparison to the determined thermodynamic solubility values (*vide supra*).

### 2.7 Interference with tubulin polymerization

Intrigued by the low micromolar to submicromolar IC_50_ values and the ability of previously reported indolo[2,3-*d*]benzazepines to act as tubulin targeting agents, we decided to find out whether either the ligands HL^1^–HL^6^ or the copper(ii) complexes 1–6 interact with the microtubule system. Research on metal complexes as inhibitors of tubulin is limited to a few Ag(i), Au(i) and Hg(ii)–carbene complexes,^27,28^ as well as to Hg(ii)-complexes that solely exploit the affinity of Hg(ii) for that protein.^26^ A single copper(ii) complex has been reported to interfere with tubulin dynamics, but the evidence for this activity was missing.^70^ All compounds were evaluated for their ability to inhibit the polymerization of purified tubulin. As a reference for comparison, combretastatin A-4 (CA-4) was used. As shown in Table 6, significant inhibition was only observed with the proligand HL^1^ and its copper(ii) complex 1 (IC_50_, 4.8 and 3.6 μM, respectively), while all the other compounds tested showed IC_50_ values ≥20 μM. This compares with an IC_50_ value of 0.9 μM for CA-4. The active proligand-complex pair was further investigated for their abilities to inhibit the binding of [^3^H]colchicine to tubulin at two different concentrations (5 and 25 μM), with tubulin and colchicine at 0.5 and 5 μM concentrations, respectively (Table 6).^71,72^ The data obtained show that these two compounds are equipotent in their ability to inhibit the binding of [^3^H]colchicine to tubulin, but are 5-fold less potent than CA-4 at the 5 μM concentration.

**Table 6:** Inhibition of tubulin polymerization and colchicine binding by compounds HL^1^, 1 and CA-4^a^

Compound	Inhibition of tubulin assembly	Inhibition of colchicine binding		
		% inhibition ± SD		
	IC_50_ ± SD (μM)	0.5 μM inhibitor	5 μM inhibitor	25 μM inhibitor
CA-4	0.91 ± 0.1	81 ± 4	98 ± 0.9	
HL^1^	4.8 ± 0.03		18 ± 5	46 ± 5
1	3.6 ± 0.5		18 ± 2	40 ± 5

a Each experiment was performed 2–3 times, and SD's are presented.

Nevertheless, comparison of the IC_50_ values for antiproliferative activity of HL^1^ and 1 in cancer cells and inhibition of pure tubulin shows that all are in the low micromolar concentration range (compare Table 4 and Table 6). This might indicate that the main mode of action of these two compounds is inhibition of tubulin assembly. Interestingly, complex formation with Cu(ii) did not result in significant differences regarding the ability to inhibit tubulin or bind to the colchicine site. These results are in good accord with a previous organometallic indolobenzazepine Ru(ii)–arene complex, which also showed potent inhibition of tubulin polymerization.^29^

The data suggest that compounds HL^1^ and 1 can be used for further optimization by fine-tuning of their electronic and steric properties by decoration of the ligand scaffold with various substituents in order to produce more potent inhibitors of the binding of [^3^H]colchicine to tubulin.

### 2.8 Molecular docking of HL^1^–HL^6^ and 1–6 into the colchicine site

Six ligands (HL^1^–HL^6^) and their copper(ii) complexes 1–6 were docked into the colchicine site of the β-subunit of tubulin (PDB ID: 1SA0, resolution 3.58 Å).^73^ The co-crystallized *N*-deacetyl-*N*-(2-mercaptoacetyl)-colchicine (DAMA-C) was removed and re-docked into the binding site to test the robustness of the scoring functions of GoldScore (GS),^74^ ChemScore (CS)^75,76^ ChemPLP (Piecewise Linear Potential)^77^ and ASP (Astex Statistical Potential)^78^ embedded in the GOLD (v2020.2.0) docking algorithm. The predicted poses were overlayed with the co-crystallized DAMA-C, and the root-mean-square deviation (RMSD) for the heavy atoms was calculated; the results are quoted in Table S2 in the ESI† with ChemPLP giving the best result of 1.1 Å, *i.e.*, a very good overlap.

The binding scores are given in Table S2 in the ESI.† All the ligands and copper(ii) complexes showed good scores, indicating reasonable binding. Only GS were run for the copper(ii) complexes as the other scoring functions are not parametrised for metals. The copper(ii) complexes and their ligands were predicted to bind with similar affinity as the DAMA-C co-crystallised ligand.

**Fig. 11:**
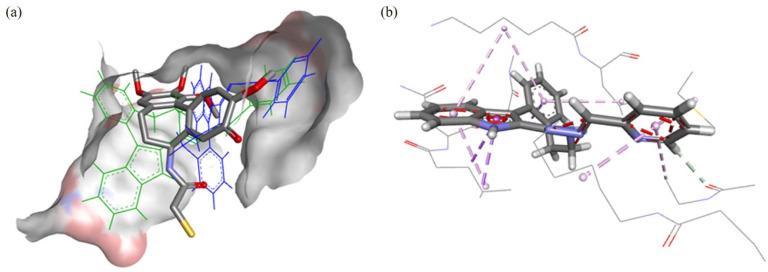
(a) The docked poses of HL^1^ in the tubulin binding site, the co-crystalised ligand DAMA-C is shown in stick format, its hydrogen atoms were omitted for clarity. The configuration of the GS prediction is shown in blue, and the ASP pose is green, both are shown as lines. The blue color depicts regions with a partial positive charge on the protein surface, while red and grey indicate regions with a partial negative charge and neutral areas, respectively. (b) The ASP predicted binding of HL^1^, the amino acids interacting with the substrate are shown as continuous lines. Dashed purple lines are used for illustration of the hydrophobic contacts, while dashed grey lines for representaton of the weak hydrogen bonding.

The modelling for the tubulin-colchicine pocket revealed that the ligands have similar predicted binding poses excluding GS. The predicted poses of HL^1^ are shown in Fig. 11a; these configurations have good overlap with DAMA-C, where the 8 membered ring and sp^2^-hybridized systems of HL^1^ occupy the same space as the 7-membered rings in DAMA-C. The configuration of HL^1^ is shown in Fig. 11b, where numerous interactions are predicted with side chain residues of the tubulin-colchicine pocket.

**Fig. 12:**
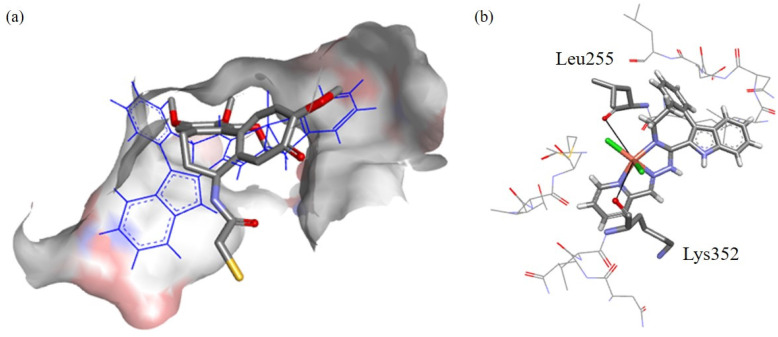
(a) The docked pose of 1 in the tubulin binding site, the co-crystalised ligand DAMA-C is shown in stick format, its hydrogen atoms were omitted for clarity. The configuration of the GS prediction is shown as blue lines. The blue color depicts regions with a partial positive charge on the protein surface, while red and grey colors indicate regions with a partial negative charge and neutral areas, respectively. (b) The predicted binding interactions of complex 1 with amino acids within 3.5 Å radius are illustrated in line format, while the potentially chelating amino acid residues βLeu255 and βLys352 are depicted in stick format. The distance between the oxygen atoms of βLeu255 and βLys352 to the copper(ii) ion (black solid line) are of 5.8 and 5.1 Å, respectively.

The predicted pose of 1 is shown in Fig. 12a. Good overlap with DAMA-C was observed, where the copper(ii) ion and chlorido co-ligands are pointing into the active site, suggesting good binding. In Fig. 12b, it can be seen that two potential groups can replace the chlorido co-ligands in the complex: the backbone carbonyls in βLeu255 and βLys352 are at 5.8 Å and 5.1 Å distance, respectively, from the bound copper(ii). The backbone carbonyls tend to be fixed in place, making them good candidates for copper(ii) binding.

The calculated molecular descriptors MW (molecular weight), log *P* (water-octanol partition coefficient), HD (hydrogen bond donors), HA (hydrogen bond acceptors), PSA (polar surface area) and RB (rotatable bonds) by using the QikProp^93^ software are summarized in Table S3 in the ESI.† As QikProp is not parameterised for metal complexes, Scigress^95^ was exploited instead with the available MW, log* P*, HD, and HA descriptors (Table S4 in the ESI†).

The values for the HL^1^–HL^6^ ligands are mainly within drug-like chemical space. HD is in lead-like chemical space, while the log *P* values are quite high in Drug-like chemical space and beyond in KDS. As for the complexes, their MWs are obviously higher than those of their counterpart ligands, with all the values in KDS. The HD are all in lead-like space and HA in drug-like space. Finally, the log *P* values are hovering around the 5 mark both in drug-like space and KDS (for the definition of lead-like, drug-like and KDS regions see ref. 79 and Table S5 in the ESI†).

The Known Drug Indexes (KDIs) for the ligands were calculated to estimate the balance of the molecular descriptors (MW, log *P*, HD, HA, PSA and RB). Both the summation of the indexes (KDI_2a_) and multiplication (KDI_2b_) methods were used^80^ as shown for KDI_2a_ in eqn (1) and for KDI_2b_ in eqn (2); the numerical results are quoted in Table S3 in the ESI.†

1 KDI_2a_ = *I*_MW_ + *I*_log *P*_ + *I*_HD_ + *I*_HA_ + *I*_RB_ + *I*_PSA_

2 KDI_2b_ = *I*_MW_ × *I*_log *P*_ × *I*_HD_ × *I*_HA_ × *I*_RB_ × *I*_PSA_

The KDI_2a_ values for the ligands are from 4.60 to 5.41 with a theoretical maximum of 6 and the average of 4.08 (±1.27) for known drugs. The KDI_2b_ values are from 0.14 to 0.52, with a theoretical maximum of 1 and with KDS average of 0.18 (±0.20). Overall these values indicate good biocompatibility, with excellent one for HL^1^ with KDI_2a_ of 5.41 and KDI_2b_ of 0.52.

## 3 Conclusion

A new series of medium-size ring systems as potential ligands for transition metals has been prepared *via* multistep synthesis. High purity of indolo[2,3-*e*]benzazocine ligands HL^1^–HL^4^, indolo[2,3-*f*]benzazonine ligands HL^5^ and HL^6^, and of copper(ii) complexes 1–6 was confirmed by HPLC-HR-MS, ^1^H and ^13^C NMR, and elemental analysis.

Crystallization of several precursors, metal-free ligands and complexes allowed for the elucidation of the solid-state preferred conformers. For indolobenzazonines the CB conformation was the most preferred as was also the case for cyclononane and derivatives, while for indolobenzazocines the twisted boat (TB) conformation was adopted in the solid state, which is different from that for cyclooctane, but similar to substituted heterocyclic cyclooctane derivatives.

The tridentate ligands HL^1^–HL^6^ with both an 8-membered benzazocine central ring and a 9-membered benzazonine ring efficiently bound Cu(ii). HL^1^, HL^5^, HL^6^, 1, 5 and 6 showed remarkable selectivity towards several cancer cell lines, with IC_50_ values in the low micromolar to sub-micromolar concentration range. The cell lines examined were Colo205, doxorubicin resistant Colo320 and MCF-7. The copper(ii) complexes 1–6 were more effective than their metal-free ligands in Colo205 cells, while in MCF-7 breast cancer cells and doxorubicin resistant Colo320 cells, the ligands exceeded in most cases the antiproliferative activity of the copper(ii) complexes. Insight into the mechanism of action provided evidence that DNA was not a primary target for the Cu(ii) complexes. However, inhibition of tubulin assembly might be the main mode of action for some particular motifs, *i.e.*, HL^1^ and 1, with little effect on activity occurring with complex formation. This was also supported by molecular docking calculations. Complex 1 is the first reported transition metal complex that binds to tubulin in the colchicine site.

## 4 Experimental section

### 4.1 Chemicals

*N*-(2-Hydroxyethyl)piperazine-*N*′-(2-ethanesulfonic acid) (HEPES), calf thymus DNA (ct-DNA, type I, fibers) and ethidium bromide (referred to as ethidium hereafter) were purchased from Sigma-Aldrich. DMSO, KCl, KOH, HCl, EDTA and potassium hydrogen phthalate were obtained from VWR International (Hungary) and used without further purification. CuCl_2_ stock solution was prepared by the dissolution of anhydrous CuCl_2_ in water, and its concentration was determined by complexometric titration with EDTA. Milli-Q water was used for the preparation of all solutions.

2-Iodophenylacetonitrile and 5-bromo-ethyl-1*H*-indole-carboxylate were purchased from ABCR. Borane solution (1 M in THF), di-*tert*-butyl-dicarbonate (Boc_2_O), dry DMF, dimethylaminopyridine (DMAP), dry acetonitrile, sodium bicarbonate, palladium(ii) acetate, Lawesson's reagent, 2-acetylpyridine and 2-formylpyridine were obtained from Fisher/Acros Organics. Ethoxy-methyl chloride was obtained from TCI. Lithium hydroxide monohydrate and triphenylphosphine were purchased from Alfa Aesar, while sodium hydride, Celite, hydrazine monohydrate and methyl iodide were from Sigma Aldrich. 1-Ethyl-3-(3-dimethylaminopropyl)carbodiimide-hydrochloride was from IRIS Biotech. Silver(i) carbonate was purchased from Merck. 2-Iodophenylethylamine and 2-bromophenylethylamine were prepared by a published method.^9^ Synthesis of 3-(2-iodophenyl)propan-1-amine (X4) is described in the ESI, see also Scheme S2.†

### 4.2 Synthesis of ligands HL^1^–HL^6^

The isolated yields and analytical data for ligands HL^1^–HL^6^ are collected in Tables S6 and S7 in the ESI.† Experimental CHN contents providing evidence for >95% purity and are within ± 0.4% of those calculated.

#### 4.2.1 HL^1^·0.2C_2_H_6_O·0.2H_2_O

To a solution of VIIa (156 mg, 0.56 mmol) in anoxic ethanol (1.5 mL), 2-formylpyridine (53.4 μL, 0.62 mmol) was added, and the resulting solution was stirred at 85 °C overnight. To the cooled solution, water (10 mL) was added, and the suspension was redissolved with ethanol (7 mL). The organic solvent was slowly removed, the resulting precipitate was isolated by filtration and washed with EtOH/H_2_O 1/1 (2 mL). The dried product was obtained as a yellow solid. Yield: 173 mg, 84%. ^1^H NMR (600 MHz, DMSO-*d*_6_) *δ* 11.74 (s, 1H, H^9^), 8.58 (d, *J* = 4.7 Hz, 1H, H^19^), 8.35 (s, 1H, H^16^), 8.32 (d, *J* = 8.0 Hz, 1H, H^22^), 7.84 (td, *J* = 7.9, 1.4 Hz, 1H, H^21^), 7.52 (d, *J* = 8.2 Hz, 1H, H^13^), 7.48 (d, *J* = 8.0 Hz, 1H, H^10^), 7.42 (t, *J* = 8.1 Hz, 2H, H^4^, H^1^), 7.40–7.29 (m, 4H, H^2^, H^3^, H^7^, H^20^), 7.24 (t, *J* = 7.6 Hz, 1H, H^12^), 7.08 (t, *J* = 7.5 Hz, 1H, H^11^), 3.61 (d, *J* = 30.0 Hz, 2H, H^6^), 2.97 (d, *J* = 85.0 Hz, 2H, H^5^). ^13^C NMR (151 MHz, DMSO-*d*_6_) *δ* 157.82 (Cq, C^8^), 154.12 (CH, C^16^), 154.11 (Cq, C^17^), 149.31 (CH, C^19^), 137.88 (Cq, C^4a^), 136.49 (Cq, C^13a^), 136.35 (CH, C^21^), 134.10 (Cq, C^13c^), 129.97 (CH, C^1^), 129.76 (CH, C^4^), 128.67 (Cq, C^9a^), 126.95 (CH, C^3^), 126.43 (Cq, C^8a^), 126.15 (CH, C^2^), 124.14 (CH, C^20^), 123.30 (CH, C^12^), 120.99 (CH, C^22^), 119.92 (CH, C^11^), 119.39 (CH, C^10^), 116.54 (Cq, C^13b^), 112.16 (CH, C^13^), 46.14 (CH_2_, C^6^), 33.44 (CH_2_, C^5^). ESI-MS (acetonitrile/methanol + 1% water), positive: *m*/*z* 366.17 [M + H]^+^ (calcd *m*/*z* for [C_23_H_20_N_5_]^+^ 366.17).

#### 4.2.2 HL^2^·0.3C_2_H_6_O

To a solution of VIIa (152 mg, 0.55 mmol) in anoxic ethanol (1.5 mL), 2-acetylpyridine (67.8 μL, 0.60 mmol) was added, and the resulting solution was stirred at 85 °C overnight. To the cooled solution, water (10 mL) was added, and the suspension was redissolved with ethanol (7 mL). The organic solvent was slowly removed, the resulting precipitate was filtered and washed with EtOH/H_2_O 1/1 (2 mL). The dried product was obtained as a yellow solid. Yield: 170 mg, 81%. ^1^H NMR (600 MHz, DMSO-*d*_6_) *δ* 11.62 (s, 1H, H^9^), 8.58 (ddd, *J* = 4.8, 1.8, 0.9 Hz, 1H, H^19^), 8.43–8.39 (m, 1H, H^22^), 7.82–7.76 (m, 1H, H^21^), 7.53 (d, *J* = 8.2 Hz, 1H, H^13^), 7.49 (t, *J* = 6.3 Hz, 1H, H^10^), 7.42 (d, *J* = 7.5 Hz, 2H, H^1^, H^4^), 7.38–7.29 (m, 3H, H^2^, H^3^, H^20^), 7.24 (ddd, *J* = 8.1, 7.0, 1.1 Hz, 1H, H^12^), 7.08 (ddd, *J* = 7.0, 5.0, 0.9 Hz, 2H, H^7^, H^11^), 3.60 (s, 2H, H^6^), 2.97 (d, *J* = 83.7 Hz, 2H, H^5^), 2.45 (s, 3H, H^23^). ^13^C NMR (151 MHz, DMSO-*d*_6_) *δ* 160.04 (Cq, C^16^), 156.14 (Cq, C^17^), 155.58 (Cq, C^8^), 148.46 (CH, C^19^), 137.95 (Cq, C^4a^), 136.38 (Cq, C^9a^), 136.01 (CH, C^21^), 134.20 (Cq, C^13c^), 129.93 (CH, C^1^), 129.74 (CH, C^4^), 129.34 (Cq, C^8a^), 126.87 (CH, C^3^), 126.57 (Cq, C^13a^), 126.10 (CH, C^2^), 123.78 (CH, C^20^), 123.16 (CH, C^12^), 120.95 (CH, C^22^), 119.86 (CH, C^11^), 119.34 (CH, C^10^), 116.06 (Cq, C^13b^), 112.14 (CH, C^13^), 46.11 (CH_2_, C^6^), 33.49 (CH_2_, C^5^), 13.37 (CH_3_, C^23^). ESI-MS (acetonitrile/methanol + 1% water), positive: *m*/*z* 380.19 [M + H]^+^ (calcd *m*/*z* for [C_24_H_22_N_5_]^+^ 380.19).

#### 4.2.3 HL^3^·0.2C_2_H_6_O

To a solution of VIIb (199 mg, 0.56 mmol) in anoxic ethanol (3 mL), 2-formylpyridine (53.4 μL, 0.62 mmol) was added, and the resulting solution was stirred at 85 °C overnight. The cooled suspension was filtered, and the solid washed with EtOH/H_2_O 1/1 (2 mL). The dried product was obtained as a yellow solid. Yield: 178 mg, 71%. ^1^H NMR (600 MHz, DMSO-*d*_6_) *δ* 11.98 (s, 1H, H^9^), 8.58 (ddd, *J* = 4.8, 1.6, 1.0 Hz, 1H, H^19^), 8.35 (s, 1H, H^16^), 8.32 (d, *J* = 8.0 Hz, 1H, H^22^), 7.84 (td, *J* = 7.5, 1.2 Hz, 1H, H^21^), 7.56 (d, *J* = 1.8 Hz, 1H, H^13^), 7.48 (d, *J* = 8.6 Hz, 1H, H^10^), 7.44 (dd, *J* = 7.3, 1.4 Hz, 1H, H^4^), 7.43–7.36 (m, 4H, H^1^, H^7^, H^11^, H^20^), 7.36–7.32 (m, 2H, H^2^, H^3^), 3.59 (d, *J* = 36.2 Hz, 2H, H^6^), 2.96 (d, *J* = 66.5 Hz, 2H, H^5^). ^13^C NMR (151 MHz, DMSO-*d*_6_) *δ* 157.32 (Cq, C^8^), 154.49 (CH, C^16^), 154.02 (Cq, C^17^), 149.34 (CH, C^19^), 137.93 (Cq, C^4a^), 136.38 (CH, C^21^), 135.10 (Cq, C^9a^), 133.34 (Cq, C^13c^), 130.14 (Cq, C^8a^), 129.86 (CH, C^1^; CH, C^4^), 128.11 (Cq, C^13a^), 127.28 (CH, C^3^), 126.37 (CH, C^2^), 125.85 (CH, C^11^), 124.23 (CH, C^20^), 121.37 (CH, C^13^), 121.06 (CH, C^22^), 115.93 (Cq, C^13b^), 114.28 (CH, C^10^), 112.47 (Cq, C^12^), 46.06 (CH_2_, C^6^), 33.32 (CH_2_, C^5^). ESI-MS (acetonitrile/methanol + 1% water), positive: *m*/*z* 444.08 [M + H]^+^ (calcd *m*/*z* for [C_23_H_19_BrN_5_]^+^ 444.08).

#### 4.2.4 HL^4^·0.4C_2_H_6_O

To a solution of VIIb (199 mg, 0.56 mmol) in anoxic ethanol (3.5 mL), 2-acetylpyridine (69.1 μL, 0.62 mmol) was added, and the resulting solution was stirred at 85 °C overnight. To the cooled solution, water (10 mL) was added, and the suspension was redissolved with ethanol (7 mL). The organic solvent was slowly removed, the resulting precipitate was isolated by filtration and washed with EtOH/H_2_O 1/1 (2 mL). The dried product was obtained as a dark-yellow solid. Yield: 150 mg, 58%. ^1^H NMR (600 MHz, DMSO-*d*_6_) *δ* 11.86 (s, 1H, H^9^), 8.58 (ddd, *J* = 4.8, 1.7, 0.9 Hz, 1H, H^19^), 8.43–8.40 (m, 1H, H^22^), 7.82–7.77 (m, 1H, H^21^), 7.56 (d, *J* = 1.9 Hz, 1H, H^13^), 7.50 (d, *J* = 8.7 Hz, 1H, H^10^), 7.45–7.40 (m, 2H, H^1^, H^4^), 7.39–7.31 (m, 4H, H^2^, H^3^, H^11^, H^20^), 7.10 (s, 1H, H^7^), 3.58 (d, *J* = 22.4 Hz, 2H, H^6^), 2.95 (d, *J* = 64.2 Hz, 2H, H^5^), 2.44 (s, 3H, H^23^). ^13^C NMR (151 MHz, DMSO-*d*_6_) *δ* 160.40 (Cq, C^16^), 156.03 (Cq, C^17^), 155.05 (Cq, C^8^), 148.47 (H, C^19^), 137.98 (Cq, C^4a^), 136.03 (CH, C^21^), 135.00 (Cq, C^9a^), 133.41 (Cq, C^13a^), 130.78 (Cq, C^8a^), 129.84 (CH, C^4^), 129.82 (CH, C^1^), 128.26 (Cq, C^13a^), 127.20 (CH, C^3^), 126.31 (CH, C^2^), 125.69 (CH, C^11^), 123.86 (CH, C^20^), 121.30 (CH, C^13^), 121.01 (CH, C^22^), 115.46 (Cq, C^13b^), 114.25 (CH, C^10^), 112.38 (Cq, C^12^), 46.00 (CH_2_, C6), 33.36 (CH_2_, C^5^), 13.40 (CH_3_, C^23^). ESI-MS (acetonitrile/methanol + 1% water), positive: *m*/*z* 458.09 [M + H]^+^ (calcd *m*/*z* for [C_24_H_21_BrN_5_]^+^ 458.09).

#### 4.2.5 HL^5^·0.75CH_2_Cl_2_

Under argon atmosphere, to a suspension of VIIc (138 mg, 0.47 mmol) in anoxic ethanol (1.8 mL), 2-acetylpyridine (59 μL, 0.53 mmol) was added, and the resulting mixture was stirred at 85 °C for 18 h. The solvent was removed under reduced pressure, and the crude solid was purified on silica using hexane : THF 3 : 7 as eluent. After evaporation of the solvent, the crude oil was dissolved in DCM (10 mL), and hexane (5 mL) was added. DCM was slowly evaporated, and the resulting solid was isolated by filtration, washed with hexane (2 mL) and dried at 50 °C *in vacuo*. Yield: 86 mg, 46%. ^1^H NMR (600 MHz, DMSO-*d*_6_) *δ* 11.74 (s, 1H, H^10^), 8.55 (d, *J* = 4.2 Hz, 1H, H^20^), 8.37 (d, *J* = 8.1 Hz, 1H, H^23^), 7.75 (td, *J* = 7.9, 1.7 Hz, 1H, H^22^), 7.47 (d, *J* = 8.2 Hz, 1H, H^14^), 7.35–7.31 (m, 2H, H^21^, H^13^), 7.29 (td, *J* = 7.5, 1.2 Hz, 1H, H^3^), 7.25 (t, *J* = 7.1 Hz, 1H, H^8^), 7.23–7.19 (m, 1H, H^4^), 7.17 (dd, *J* = 11.6, 4.5 Hz, 2H, H^2^, H^11^), 7.06 (d, *J* = 6.9 Hz, 1H, H^1^), 7.03 (t, *J* = 7.7 Hz, 1H, H^12^), 3.60–2.90 (s, broad, 2H, H^7^, overlapped with water), 2.80–2.20 (d, broad, 2H, H^5^, overlapped with DMSO), 2.27 (d, *J* = 6.9 Hz, 3H, H^24^), 1.76 (s, 2H, H^6^). ^13^C NMR (151 MHz, DMSO-*d*_*6*_) *δ* 158.98 (Cq, C^17^), 156.16 (Cq, C^18^), 155.58 (Cq, C^9^), 148.36 (CH, C^20^), 143.61 (Cq, C^4a^), 135.85 (CH, C^22^), 135.19 (Cq, C^14c^), 133.33 (Cq, C^10a^), 131.07 (CH, C^1^), 130.70 (Cq, C^9a^), 129.23 (CH, C^13^), 127.69 (Cq, C^14a^), 127.67 (CH, C^3^), 125.11 (CH, C^11^), 123.62 (CH, C^21^), 122.34 (CH, C^4^), 120.86 (CH, C^23^), 119.56 (CH, C^12^), 119.03 (CH, C^2^), 116.33 (Cq, C^14b^), 111.70 (CH, C^14^), 33.32 (CH_2_, C^6^), 13.04 (CH_3_, C^24^). Carbon resonances for C^5^ and C^7^ could not be seen. ESI-MS (acetonitrile/methanol + 1% water), positive: *m*/*z* 394.22 [M + H]^+^ (calcd *m*/*z* for [C_25_H_24_N_5_]^+^ 394.20).

#### 4.2.6 HL^6^·CH_2_Cl_2_

Under argon atmosphere, to a suspension of VIId (352 mg, 0.95 mmol) in anoxic ethanol (4 mL), 2-acetylpyridine (118 μL, 1.05 mmol) was added, and the resulting mixture was stirred at 85 °C for 18 h. The solvent was removed under reduced pressure, and the crude solid was purified on silica using hexane : THF 3 : 7 as eluent. After evaporation of the solvent, the crude oil was dissolved in DCM (10 mL), and hexane (5 mL) was added. DCM was slowly evaporated, and the resulting crystals were isolated by filtration, washed with hexane (2 mL) and dried at 50 °C *in vacuo*. Yield: 86 mg. 41%. ^1^H NMR (600 MHz, DMSO-*d*_6_) *δ* 12.00 (s, 1H, H^10^), 8.55 (ddd, *J* = 4.8, 1.7, 0.9 Hz, 1H, H^20^), 8.36 (dd, *J* = 5.0, 4.1 Hz, 1H, H^23^), 7.75 (ddd, *J* = 8.0, 7.5, 1.8 Hz, 1H, H^22^), 7.45 (t, *J* = 7.0 Hz, 1H, H^14^), 7.36–7.29 (m, 4H, H^21^, H^12^, H^4^, H^3^), 7.27 (t, *J* = 7.2 Hz, 1H, H^8^), 7.23 (d, *J* = 1.9 Hz, 1H, H^11^), 7.18 (td, *J* = 7.3, 1.9 Hz, 1H, H^2^), 7.08–7.05 (m, 1H, H^1^), 3.40–2.80 (d, broad, 2H, H^7^, overlapped with water), 2.75–2.10 (d, broad, 2H, H^5^, overlapped with DMSO), 2.30–2.25 (m, 3H, H^24^), 1.77 (d, *J* = 13.8 Hz, 2H, H^6^). ^13^C NMR (151 MHz, DMSO-*d*_6_) *δ* 159.80 (Cq, C^17^), 156.57 (Cq, C^18^), 155.43 (Cq, C^9^), 148.87 (CH, C^20^), 144.18 (Cq, C^4a^), 136.37 (CH, C^22^), 134.37 (Cq, C^10a^), 132.92 (Cq, C^14c^), 132.77 (Cq, C^9a^), 131.52 (CH, C^1^), 129.85 (Cq, C^14a^), 129.83 (CH, C^4^), 128.49 (CH, C^3^), 125.77 (CH, C^2^), 125.39 (CH, C^12^), 124.19 (CH, C^21^), 121.48 (CH, C^11^), 121.40 (CH, C^23^), 116.36 (Cq, C^14b^), 114.39 (CH, C^14^), 112.50 (Cq, C^13^), 33.73 (CH_2_, C^6^), 13.55 (CH_3_, C^24^). Carbon resonances for C^5^ and C^7^ could not be seen. ESI-MS (acetonitrile/methanol + 1% water), positive: *m*/*z* 474.17 [M + H]^+^ (calcd *m*/*z* for [C_25_H_23_BrN_5_]^+^ 477.11).

Single crystals of X-ray diffraction quality of HL^4^ and HL^6^ were generated by slow diffusion of the solvent of a diluted solution of ligands in DCM into methylcyclohexane.

### 4.3 Synthesis of copper(ii) complexes 1–6

The isolated yields and analytical data for Cu(ii) complexes 1–6 are collected in Tables S7 and S8 in the ESI.† Experimental CHN contents provide evidence for >95% purity and are within ± 0.4% of those calculated.

#### 4.3.1 Complex 1

To a solution of HL^1^ (67 mg, 0.18 mmol) in isopropanol (10 mL) at 70 °C a solution of CuCl_2_·2H_2_O (31 mg, 0.18 mmol) in methanol (100 μL) was added. The mixture was refluxed for 30 min and cooled to 4 °C. After 2 days the precipitate was isolated by filtration, washed with isopropanol (1 mL) and dried *in vacuo* to give a green-brownish solid. Yield: 55.7 mg, 70%. ESI-MS (acetonitrile/methanol + 1% water), positive: *m*/*z* 427.08 [M − HCl − Cl]^+^ (calcd *m*/*z* for [C_23_H_18_CuN_5_]^+^ 427.08).

#### 4.3.2 Complex 2

To a solution of HL^2^ (62 mg, 0.16 mmol) in isopropanol (20 mL) at 70 °C a solution of CuCl_2_·2H_2_O (28 mg, 0.16 mmol) in methanol (100 μL) was added. The dark-red solution was refluxed for 30 min. The solution was concentrated by one half, and the product was precipitated with diethyl ether (40 mL). The product was isolated by filtration, washed with diethyl ether (2 mL) and dried *in vacuo* to give a bright green solid. Yield: 47 mg, 56%. ESI-MS (acetonitrile/methanol + 1% water), positive: *m*/*z* 441.10 [M − HCl − Cl]^+^ (calcd *m*/*z* for [C_24_H_20_CuN_5_]^+^ 441.10).

#### 4.3.3 Complex 3·0.2C_2_H_6_O·0.3H_2_O

To a solution of HL^3^ (60 mg, 0.14 mmol) in isopropanol (60 mL) at 70 °C a solution of CuCl_2_·2H_2_O (23 mg, 0.14 mmol) in methanol (100 μL) was added. The mixture was refluxed for 30 min. The solution was concentrated to ∼15 mL and cooled to 4 °C overnight. The product was isolated by filtration, washed with diethyl ether (2 mL) and dried *in vacuo* to give a green solid. Yield: 72 mg, 92%. ESI-MS (acetonitrile/methanol + 1% water), positive: *m*/*z* 506.99 [M − HCl − Cl]^+^ (calcd *m*/*z* for [C_23_H_17_BrCuN_5_]^+^ 507.00).

#### 4.3.4 Complex 4·0.9C_3_H_8_O

To a solution of HL^4^ (61 mg, 0.13 mmol) in isopropanol (10 mL) at 70 °C a solution of CuCl_2_·2H_2_O (23 mg, 0.13 mmol) in methanol (100 μL) was added. The mixture was refluxed for 30 min and cooled to 4 °C overnight. The dark-green precipitate was isolated by filtration, washed with diethyl ether (2 mL) and dried *in vacuo* to give a green solid. Yield: 65 mg, 81%. ESI-MS (acetonitrile/methanol + 1% water), positive: *m*/*z* 521.01 [M − HCl − Cl]^+^ (calcd *m*/*z* for [C_24_H_19_BrCuN_5_]^+^ 521.01).

#### 4.3.5 Complex 5·1.75H_2_O

To a solution of HL^5^ (85 mg, 0.22 mmol) in isopropanol (15 mL) at 60 °C a solution of CuCl_2_·2H_2_O (37 mg, 0.22 mmol) in methanol (100 μL) was added. The mixture was refluxed for 30 min and cooled to 4 °C overnight. The bright-green precipitate was isolated by filtration, washed with methanol (2 mL) and dried *in vacuo* to give a bright-green solid. Yield: 66 mg, 58%. ESI-MS (acetonitrile/methanol + 1% water), positive: *m*/*z* 491.16 [M − Cl]^+^ (calcd *m*/*z* for [C_25_H_23_ClCuN_5_]^+^ 491.10).

#### 4.3.6 Complex 6·C_3_H_8_O·0.75H_2_O

To a solution of HL^6^ (38 mg, 0.08 mmol) in isopropanol (7 mL) at 60 °C a solution of CuCl_2_·2H_2_O (13.5 mg, 0.08 mmol) in methanol (100 μL) was added. The mixture was refluxed for 30 min and cooled to 4 °C overnight. The bright-green precipitate was isolated by filtration, washed with methanol (2 mL) and dried *in vacuo* to give a green-brownish solid. Yield: 35 mg, 73%. ESI-MS (acetonitrile/methanol + 1% water), positive: *m*/*z* 571.01 [M − Cl]^+^ (calcd *m*/*z* for [C_25_H_22_BrClCuN_5_]^+^ 571.01).

UV–vis kinetic measurements for 1–6 over 72 h are shown in Fig. S6–S11 in the ESI.†

Single crystals of X-ray diffraction quality of 2, 4 and 5 were grown by slow vapor diffusion of diethyl ether into a concentrated solution of complexes in DMF. Single crystals of X-ray diffraction quality of 5′ were grown by slow evaporation of the solvent of a diluted solution of 5 in a mixture of isopropanol and methanol.

#### 4.3.7 Crystallographic structure determination

The measurements were performed on Bruker X8 APEXII CCD (VIa·0.5MeOH, 5′·2DMF), Bruker D8 Venture (HL^4^, 2·2DMF) and STOE STADIVARI (IIIa, Vd·Vd^EOM^, HL^6^·DCM, 4·2DMF·H_2_O, 5·2DMF) diffractometers. Crystal data, data collection parameters, and structure refinement details are given in Tables S9 and S10 in the ESI.† The structures were solved by direct methods and refined by full matrix least-squares techniques. Non-H atoms were refined with anisotropic displacement parameters. H atoms were inserted in calculated positions and refined with a riding model. The following computer programs and hardware were used: structure solution, SHELXS-2014 and refinement, SHELXL-2014;^81^ molecular diagrams, ORTEP;^82^ computer, IntelCoreDuo. CCDC 2261958–2261966.†

### 4.4 Spectrophotometric titrations and solubility tests

The UV–vis spectra were recorded on an Agilent Cary 8454 diode array spectrophotometer between 200 and 800 nm. Spectrophotometric titrations were carried out on samples containing the ligand (HL^1^, HL^2^, HL^5^ or HL^1^_(7)_) or the ligand and Cu(ii) ions together at different ligand-to-metal ratios (1 : 1, 1.5 : 1, 2 : 1) by a KOH solution in a DMSO : water 30 : 70 (v/v) mixture as solvent at 25.0 ± 0.1 °C in the pH range from 1.7 to 12.7 using 0.1 M KCl ionic strength. The concentration of ligands was 10 μM. The path length was 5 cm. For pH measurements and titrations, an Orion 710A pH-meter equipped with a Metrohm combined electrode (type 6.0234.100) and a Metrohm 665 Dosimat burette was used. Blank titrations (HCl *vs.* KOH) were performed similarly to the method proposed by Irving *et al.* for pure aqueous solutions to calibrate the electrode system to the pH = −log[H+] scale in the DMSO–water solvent mixture.^83^ The water ionization constant (p*K*_w_) was determined to be 14.52 ± 0.05 on average, which is in line with the literature data.^84^ During the titrations argon was passed over the solutions. The computer program PSEQUAD was used to calculate the proton dissociation constants (p*K*_a_) of the ligands, the overall stability constants (log *β*) of the Cu(ii) complexes and the individual spectra of the various species present in solution.^85^ In the general equilibrium *p*M + *q*L + *r*H ⇌ M_*p*_L_*q*_H_*r*_ the stability constants is defined as *β*(M_*p*_L_*q*_H_*r*_) = [M_*p*_L_*q*_H_*r*_]/[M]^*p*^[L]^*q*^[H]^*r*^, where M denotes the Cu(ii) ion and L the completely deprotonated ligand. The calculations were always performed from experimental data where no precipitation was observed during titration.

The thermodynamic solubility (*S*) of the studied compounds was determined in saturated solutions at pH 7.40 (20 mM HEPES buffer) in the absence of DMSO at 25.0 ± 0.1 °C. UV–vis spectrophotometry was applied to determine the concentration of the compounds using calibrating samples of the compounds of known concentrations dissolved in 100% DMSO and 50% (v/v) DMSO/buffered aqueous solutions.

### 4.5 ct-DNA binding studies

Fluorescence measurements were carried out on a Fluoromax (Horiba Jobin Yvon) fluorimeter. Stock solutions of the complexes and ligands were prepared in pure DMSO (*c* = 1 or 0.5 mM). Stock solution of ct-DNA was prepared as described recently.^86^ The sample volume was 20.0 mL and contained 0.5 μM ct-DNA expressed in base pairs, 0.25 μM ethidium and different concentrations of complexes 1, 2, 4, 5, 6, 4_(6)_, 4_(7)_ or ligands (HL^1^, HL^2^, HL^5^, HL^6^) in 10 mM HEPES buffer (pH 7.40). DMSO content of the samples varied between 0 and 1% (v/v). The excitation wavelength was 510 nm, and the fluorescence emission was measured in the range of 530–750 nm. Corrections for self-absorbance and inner filter effect were performed as described previously.^87^

### 4.6 Cell lines

Four cell lines were used in this study: the doxorubicin-sensitive Colo205 (CCL-222, ATCC, Manassas, VA, USA) human colon adenocarcinoma cell line; the doxorubicin-resistant Colo320/MDR-LRP expressing P-glycoprotein (P-gp, ABCB1) (MDR1)-LRP (CCL-220.1, ATCC) human colon adenocarcinoma cell line; the hormone-responsive MCF-7 breast cancer cell line and the normal MRC-5 human embryonal lung fibroblast cell line (CCL-171, ATCC). The human cancer cell lines were purchased from LGC Promochem (Teddington, UK), and the MRC-5 cell line is a Sigma-Aldrich (Merck KGaA, Darmstadt, Germany) product. The Colo205 and Colo320 cell lines were cultured in RPMI 1640 medium supplemented with 10% fetal bovine serum (FBS), 2 mM l-glutamine, 1 mM Na-pyruvate and 10 mM HEPES. The MCF-7 and MRC-5 cells were cultured in Eagle's Minimal Essential Medium (EMEM) containing 4.5 g L^−1^ of glucose and supplemented with a non-essential amino acid mixture, a selection of vitamins and 10% FBS. All cell lines were incubated at 37 °C, 5% CO_2_, 95% air atmosphere. The cells were detached with Trypsin-Versene (EDTA) solution for 5 min at 37 °C.

### 4.7 *In vitro* assay for cytotoxic effect

Prior to the cytotoxicity assays 10 mM stock solutions of the tested compounds were prepared in DMSO. These stock solutions were diluted with the culture medium (EMEM or RPMI 1640), and twofold serial dilutions of the compounds were prepared in 100 μL of the appropriate medium. The final DMSO concentration did not exceed 1% (v/v) in the medium and no inhibition of cell viability was observed at this DMSO concentration. The positive control was doxorubicin (Merck), and CuCl_2_ was also tested. All cancer cells were treated with Trypsin-Versene (EDTA) solution. A cell density of 1 × 10^4^ cells was adjusted for the colon adenocarcinoma cells in 100 μL of RPMI 1640 medium and were added to each well, except for the medium control wells. The final volume was 200 μL in the wells containing compounds and cells. The MCF-7 and MRC-5 cells were seeded overnight prior to the assay. The following day, the serial dilution of the compounds were made in separate plates and added to the cells. The culture plates were incubated at 37 °C for 72 h; and at the end of the incubation period, 20 μL of 5 mg mL^−1^ 3-(4,5-dimethylthiazol-2-yl)-2,5-diphenyltetrazolium bromide (MTT) solution was added to each well and incubated for 4 h at 37 °C. The plates were further incubated at 37 °C overnight following the addition of 100 μL of sodium dodecyl sulfate (SDS) solution (10% in 0.01 M HCl). The optical density (OD) was measured at 450 nm (ref. 620 nm) with a Multiscan EX ELISA reader to follow the cell growth. Inhibition of cell growth (expressed as IC_50_: inhibitory concentration that reduces by 50% the growth of the cells exposed to the tested compounds) was determined from the sigmoid curve, where 100 − ((OD_sample_ − OD_medium control_)/(OD_cell control_ − OD_medium control_)) × 100 values were plotted against the logarithm of compound concentrations. The sigmoidal dose–response model (comparing variable and fixed slopes) of the GraphPad Prism software^88^ was used to fit the curves. The IC_50_ values are averages from at least 3 independent experiments.

### 4.8 Molecular modelling

The ligands and copper(ii) complexes were docked against the crystal structure of tubulin-2-mercapto-*N*-[1,2,3,10-tetramethoxy-9-oxo-5,6,7,9-tetrahydro-benzo[A]heptalene-7-yl]acetamide (DAMA-C) (PDB ID: 1SA0, resolution 3.58 Å),^73^ which was obtained from the Protein Data Bank (PDB).^89,90^ The GOLD (v2020.2.0) software suite was used to prepare the crystal structures for docking, as reported recently.^30^ The docking centre for the binding pocket was defined as the position of the co-crystallised compounds with a 10 Å radius. The implemented in GoldScore(GS)^74^ and ChemScore(CS)^75,76^ ChemPLP(Piecewise Linear Potential)^77^ and ASP(Astex Statistical Potential)^78^ scoring functions were used to predict the binding modes and relative energies of the compounds using the GOLD (v2020.2.0) software suite. The GOLD docking algorithm was already shown to be an excellent modelling tool.^91,92^ The crystal structures of HL^2^, HL^4^, HL^6^, 2, 4, and 5 were used for docking and the structures of HL^1^, HL^3^, HL^5^, 1, 3, and 6 were modified in Discovery Studio directly from them.

The QikProp v6.2^93^ software package was used to calculate the molecular descriptors of the molecules. The reliability of QikProp was established for the calculated descriptors.^94^ Furthermore, the Scigress version FJ 2.6 program^95^ was used to calculate the molecular descriptors for the complexes. The Known Drug Indexes (KDI) were calculated from the molecular descriptors as described by Eurtivong and Reynisson.^80^ For application in Excel, columns for each property were created and the following equations used do derive the KDI numbers for each descriptor: KDI MW = EXP(−((MW − 371.76)^2^)/(2*(112.76^2^))), KDI log *P* = EXP(–((log *P* − 2.82)^2^)/(2*(2.21^2^))), KDI HD = EXP(–((HD − 1.88)^2^)/(2*(1.7^2^))), KDI HA = EXP(–((HA − 5.72)^2^)/(2*(2.86^2^))), KDI RB = EXP(–((RB − 4.44)^2^)/(2*(3.55^2^))), and KDI PSA = EXP(–((PSA − 79.4)^2^)/(2*(54.16^2^))). These equations could simply be copied into Excel and the descriptor name (*e.g.*, MW) substituted with the value in the relevant column. To derive KDI_2A_, this equation was used = (KDI MW + KDI log P + KDI HD + KDI HA + KDI RB + KDI PSA) and for KDI_2B_ = (KDI MW × KDI log *P* × KDI HD × KDI HA × KDI RB × KDI PSA).

### 4.9 Tubulin assays

The methods used to determine the IC_50_ values for tubulin assembly and inhibition of [^3^H]colchicine binding were recently described in detail.^96^ In brief, for the assembly assay, 0.25 mL reaction mixtures contained 1.0 mg mL^−1^ (10 μM) tubulin, 0.8 M monosodium glutamate (pH 6.6 with HCl in 2.0 M stock solution), 4% (v/v) DMSO (compound solvent), varying compound concentrations, and 100 μM GTP. All components except GTP were preincubated at 30 °C for 15 min in a 0.24 mL volume. Reaction mixtures were then placed on ice, and the GTP was added in a 10 μL volume. Assembly was monitored in Beckman DU7400/7500 spectrophotometers equipped with electronic temperature controllers and driven by a custom program. The samples were transferred to cuvettes held at 0 °C, and the temperature was jumped to 30 °C over about 30 s. Change in turbidity was measured for 20 min at 350 nm, and the IC_50_ was defined as the compound concentration that reduced the extent of reaction by 50%. For the colchicine binding assay, reaction mixtures contained 0.05 mg mL^−1^ (0.5 μM) tubulin, 5.0 μM [^3^H]colchicine, 5% (v/v) DMSO (compound solvent), compound as indicated, and components found to stabilize the colchicine binding activity of tubulin. Incubation was for 10 min at 37 °C, at which time the amount of colchicine bound to the tubulin is 40–60% of maximum. Each reaction mixture was diluted with 2.0 mL of ice cold water and poured onto a single Whatman DEAE-cellulose filter (tubulin binds tightly to the filter, and unbound colchicine passes through). Gravity filtration was used for the samples, and the filters were then washed with three 2.0 mL aliquots of ice cold water (vacuum aspiration). The filters were counted in a Beckman scintillation counter, with correction for the amount of radiolabel bound to filters in the absence of tubulin.

## Conflicts of interest

The authors declare no competing interest.

## Supplementary Material

DT-052-D3DT01632C-s001

DT-052-D3DT01632C-s002

## References

[cit1] Greenwell M., Rahman P. K. S. M. (2015). Int. J. Pharma Sci. Res..

[cit2] Linington R. G., Williams D. E., Tahir A., Van Soest R., Andersen R. J. (2003). Org. Lett..

[cit3] Akunuri R., Vadakattu M., Bujji S., Veerareddy V., Madhavi Y. V., Nanduri S. (2021). Eur. J. Med. Chem..

[cit4] Martino E., Casamassima G., Castiglione S., Cellupica E., Pantalone S., Papagni F., Rui M., Siciliano A. M., Collina S. (2018). Bioorg. Med. Chem. Lett..

[cit5] Brancale A., Silvestri R. (2007). Med. Res. Rev..

[cit6] Singh A. K., Raj V., Saha S. (2017). Eur. J. Med. Chem..

[cit7] Wan Y., Li Y., Yan C., Yan M., Tang Z. (2019). Eur. J. Med. Chem..

[cit8] Putey A., Joucla L., Picot L., Besson T., Joseph B. (2007). Tetrahedron.

[cit9] Putey A., Popowycz F., Do Q.-T., Bernard P., Talapatra S. K., Kozielski F., Galmarini C. M., Joseph B. (2009). J. Med. Chem..

[cit10] Wang N., Switalska M., Wang L., Shaban E., Hossain M. I., El Sayed I. E. T., Wietrzyk J., Inokuchi T. (2019). Molecules.

[cit11] Lavrado J., Moreira R., Paulo A. (2010). Curr. Med. Chem..

[cit12] Parvatkar P. T., Parameswaran P. S., Tilve S. G. (2011). Curr. Org. Chem..

[cit13] Vianney Y. M., Preckwinkel P., Mohr S., Weisz K. (2020). Chem. – Eur. J..

[cit14] Chen Q., Cui W., Cheng Y., Zhang F., Ji M. (2011). J. Mol. Model..

[cit15] Denis J. G., Franci G., Altucci L., Aurrecoechea J. M., De Lera A. R., Álvarez R. (2015). Org. Biomol. Chem..

[cit16] Knockaert M., Wieking K., Schmitt S., Leost M., Grant K. M., Mottram J. C., Kunick C., Meijer L. (2002). J. Biol. Chem..

[cit17] Zaharevitz D. W., Gussio R., Leost M., Senderowicz A. M., Lahusen T., Kunick C., Meijer L., Sausville E. A. (1999). Cancer Res..

[cit18] Schultz C., Link A., Leost M., Zaharevitz D. W., Gussio R., Sausville E. A., Meijer L., Kunick C. (1999). J. Med. Chem..

[cit19] Keller L., Beaumont S., Liu J. M., Thoret S., Bignon J. S., Wdzieczak-Bakala J., Dauban P., Dodd R. H. (2008). J. Med. Chem..

[cit20] La Regina G., Coluccia A., Naccarato V., Silvestri R. (2019). Eur. J. Pharm. Sci..

[cit21] Roll-Mecak A. (2020). Dev. Cell.

[cit22] Čermák V., Dostál V., Jelínek M., Libusová L., Kovář J., Rösel D., Brábek J. (2020). Eur. J. Cell Biol..

[cit23] Brouhard G. J., Rice L. M. (2018). Nat. Rev. Mol. Cell Biol..

[cit24] Akhmanova A., Steinmetz M. O. (2015). Nat. Rev. Mol. Cell Biol..

[cit25] Risinger A. L., Giles F. J., Mooberry S. L. (2008). Cancer Treat. Rev..

[cit26] Duhr E. F., Pendergrass J. C., Slevin J. T., Haley B. E. (1993). Toxicol. Appl. Pharmacol..

[cit27] Iacopetta D., Rosano C., Sirignano M., Mariconda A., Ceramella J., Ponassi M., Saturnino C., Sinicropi M. S., Longo P. (2020). Pharmaceuticals.

[cit28] Khanna S., Jana B., Saha A., Kurkute P., Ghosh S., Verma S. (2014). Dalton Trans..

[cit29] Wittmann C., Sivchenko A. S., Bacher F., Tong K. K. H., Guru N., Wilson T., Gonzales J., Rauch H., Kossatz S., Reiner T., Babak M. V., Arion V. B. (2022). Inorg. Chem..

[cit30] Wittmann C., Bacher F., Enyedy E. A., Dömötör O., Spengler G., Madejski C., Reynisson J., Arion V. B. (2022). J. Med. Chem..

[cit31] Bacher F., Wittmann C., Nové M., Spengler G., Marć M., Enyedy E. A., Darvasiová D., Rapta P., Reiner T., Arion V. B. (2019). Dalton Trans..

[cit32] Primik M. F. , Filak L. K. and Arion V. B., in Advances in Organometallic Chemistry and Catalysis: The Silver/Gold Jubilee International Conference on Organometallic Chemistry Celebratory Book, ed. A. J. L. Pombeiro, John Wiley & Sons, Inc., 1st edn, 2014, pp. 605–617

[cit33] Filak L. K., Mühlgassner G., Jakupec M. A., Heffeter P., Berger W., Arion V. B., Keppler B. K. (2010). J. Biol. Inorg. Chem..

[cit34] Primik M. F., Mühlgassner G., Jakupec M. A., Zava O., Dyson P. J., Arion V. B., Keppler B. K. (2010). Inorg. Chem..

[cit35] Dobrov A., Arion V. B., Kandler N., Ginzinger W., Jakupec M. A., Rufińska A., von Keyserlingk N. G., Galanski M., Kowol C., Keppler B. K. (2006). Inorg. Chem..

[cit36] Schmid W. F., John R. O., Mühlgassner G., Heffeter P., Jakupec M. A., Galanski M., Berger W., Arion V. B., Keppler B. K. (2007). J. Med. Chem..

[cit37] Primik M. F., Göschl S., Jakupec M. A., Roller A., Keppler B. K., Arion V. B. (2010). Inorg. Chem..

[cit38] Ge E. J., Bush A. I., Casini A., Cobine P. A., Cross J. R., DeNicola G. M., Dou Q. P., Franz K. J., Gohil V. M., Gupta S., Kaler S. G., Lutsenko S., Mittal V., Petris M. J., Polishchuk R., Ralle M., Schilsky M. L., Tonks N. K., Vahdat L. T., Van Aelst L., Xi D., Yuan P., Brady D. C., Chang C. J. (2022). Nat. Rev. Cancer.

[cit39] Babak M. V., Ahn D. (2021). Biomedicines.

[cit40] Denoyer D., Masaldan S., La Fontaine S., Cater M. A. (2015). Metallomics.

[cit41] da Silva D. A., De Luca A., Squitti R., Rongioletti M., Rossi L., Machado C. M. L., Cerchiaro G. (2022). J. Inorg. Biochem..

[cit42] Shanbhag V. C., Gudekar N., Jasmer K., Papageorgiou C., Singh K., Petris M. J. (2021). Biochim. Biophys. Acta, Mol. Cell Res..

[cit43] Santini C., Pellei M., Gandin V., Porchia M., Tisato F., Marzano C. (2014). Chem. Rev..

[cit44] Kuznetcova I., Bacher F., Alfadul S. M., Tham M. J. R., Ang W. H., Babak M. V., Rapta P., Arion V. B. (2022). Inorg. Chem..

[cit45] Wittmann C., Gruene T., Prado-Roller A., Aranđelović S., Reynisson J., Arion V. B. (2022). Inorganics.

[cit46] Addison A. W., Rao T. N., Reedijk J., Van Rijn J., Verschoor G. C. (1984). Dalton Trans..

[cit47] Hendrickson J. B. (1964). J. Am. Chem. Soc..

[cit48] Dale J. (1973). Acta Chem. Scand..

[cit49] Dorofeeva O., Masturyukov V., Allinger N., Almenningen A. (1990). J. Phys. Chem..

[cit50] Anet F. A. L., Wagner J. J. (1971). J. Am. Chem. Soc..

[cit51] Anet F. A. L., Krane J. (1980). Isr. J. Chem..

[cit52] Samuel G., Weiss R. (1969). Tetrahedron Lett..

[cit53] Fürstner A., Radkowski K., Peters H., Seidel G., Wirtz C., Mynott R., Lehmann C. W. (2007). Chem. – Eur. J..

[cit54] Dahl S., Groth P., Sood M. S., Nielsen B. E., Ljunggren H., Ehrenberg L. (1971). Acta Chem. Scand..

[cit55] Dorofeeva O. V., Mastryukov V. S., Allinger N. L., Almenningen A. (1985). J. Phys. Chem..

[cit56] Pakes P. W., Rounds T. C., Strauss H. L. (1981). J. Phys. Chem..

[cit57] Zou W., Tao Y., Kraka E. (2020). J. Chem. Phys..

[cit58] Allen F. H., Howard J. A. K., Pitchford N. A. (1996). Acta Crystallogr..

[cit59] Bharadwaj R. K. (2000). Mol. Phys..

[cit60] Huang Y., Gilson D. F. R., Butler I. S. (1991). J. Phys. Chem..

[cit61] Dragojlovic V. (2015). ChemTexts.

[cit62] Kolossvary I., Guida W. C. (1993). J. Am. Chem. Soc..

[cit63] Maryanoff B., Almond H. (1986). J. Org. Chem..

[cit64] Maryanoff B., Parvez M., Olofson R. A. (1990). J. Org. Chem..

[cit65] Pakes P. W., Rounds T. C., Strauss H. L. (1981). J. Phys. Chem..

[cit66] Miller R. W., McPhail A. T. (1979). J. Chem. Soc., Perkin Trans. 2.

[cit67] Witosińska A., Musielak B., Serda P., Owińska M., Rys B. (2012). J. Org. Chem..

[cit68] Yepes A. F., Jaimes E., Bahsas A., Palma A., Hursthouse M. B., Cobo J., Glidewell C. (2010). Acta Cryst..

[cit69] Sancha S. A. R., Szemerédi N., Spengler G., Ferreira M.-J. U. (2023). Int. J. Mol. Sci..

[cit70] Becco L., Rodríguez A., Bravo M. E., Prieto M. J., Ruiz-Azuara L., Garat B., Moreno V., Gambino D. (2012). J. Inorg. Biochem..

[cit71] Hamel E. (2003). Cell Biochem. Biophys..

[cit72] Verdier-Pinard P., Lai J.-Y., Yoo H.-D., Yu J., Marquez B., Nagle D. G., Nambu M., White J. D., Falck J. R., Gerwick W. H., Day B. W., Hamel E. (1998). Mol. Pharmacol..

[cit73] Ravelli R. B. G., Gigant B., Curmi P. A., Jourdain I., Lachkar S., Sobel A., Knossow M. (2004). Nature.

[cit74] Jones G., Willett P., Glen R. C., Leach A. R., Taylor R. (1997). J. Mol. Biol..

[cit75] Eldridge M. D., Murray C. W., Auton T. R., Paolini G. V., Mee R. P. (1997). J. Comput. Aided Mol. Des..

[cit76] Verdonk M. L., Cole J. C., Hartshorn M. J., Murray C. W., Taylor R. D. (2003). Proteins: Struct., Funct., Bioinf..

[cit77] Korb O., Stützle T., Exner T. E. (2009). J. Chem. Inf. Model..

[cit78] Mooij W. T. M., Verdonk M. L. (2005). Proteins: Struct., Funct., Bioinf..

[cit79] Zhu F., Logan G., Reynisson J. (2012). Mol. Inf..

[cit80] Eurtivong C., Reynisson J. (2019). Mol. Inf..

[cit81] Sheldrick G. M. (2008). Acta Crystallogr., Sect. A: Found. Crystallogr..

[cit82] Burnett M. N. and Johnson C. K., ORTEP-III: Oak Ridge Thermal Ellipsoid Plot Program for Crystal Structure Illustrations, Oak Ridge National Laboratory, Oak Ridge, TN, 1996

[cit83] Irving H. M., Miles M. G., Pettit L. D. (1967). Anal. Chim. Acta.

[cit84] Enyedy É. A., Nagy N. V., Zsigó É., Kowol C. R., Arion V. B., Keppler B. K., Kiss T. (2010). Eur. J. Inorg. Chem..

[cit85] Zekany L. and Nagypal I., PSEQUAD A Comprehensive Program for the Evaluation of Potentiometric and/or Spectrophotometric Equilibrium Data Using Analytical Derivatives, Plenum Press, New York, 1985

[cit86] Dömötör O., de Almeida R. F. M., Côrte-Real L., Matos C. P., Marques F., Matos A., Real C., Kiss T., Enyedy É. A., Helena M., Tomaz A. I. (2017). J. Inorg. Biochem..

[cit87] Dömötör O., Enyedy É. A. (2019). J. Biol. Inorg. Chem..

[cit88] GraphPad Software, Inc., 2007

[cit89] Berman H. M. (2000). Nucleic Acids Res..

[cit90] Berman H., Henrick K., Nakamura H. (2003). Nat. Struct. Mol. Biol..

[cit91] Wang Z., Sun H., Yao X., Li D., Xu L., Li Y., Tian S., Hou T. (2016). Phys. Chem. Chem. Phys..

[cit92] Bissantz C., Folkers G., Rognan D. (2000). J. Med. Chem..

[cit93] QikProp, version 6.2; Schrödinger: New York, NY, USA, 2021

[cit94] Ioakimidis L., Thoukydidis L., Mirza A., Naeem S., Reynisson J. (2008). QSAR Comb. Sci..

[cit95] Scigress Ultra V. F.J 2.6, (EU 3.1.7); Fujitsu Limited: Minato City, Tokyo, 2016

[cit96] Saito Y., Taniguchi Y., Hirazawa S., Miura Y., Tsurimoto H., Nakayoshi T., Oda A., Hamel E., Yamashita K., Goto M., Nakagawa-Goto K. (2021). Eur. J. Med. Chem..

