# Supplementary material for: Indolo[2,3-e]benzazocines and indolo[2,3-f]benzazonines and their copper(ii) complexes as microtubule destabilizing agents
Source: Dalton Trans. 2023 Jul 4;52(29):9964–82. doi: 10.1039/d3dt01632c (PMC10368209; doi:10.1039/d3dt01632c)
Supplement: DT-052-D3DT01632C-s001 [file DT-052-D3DT01632C-s001.pdf]

Electronic Supplementary Material (ESI) for Dalton Transactions.
This journal is © The Royal Society of Chemistry 2023

Electronic Supporting Information

for

Indolo[2,3-*e*]benzazocines and indolo[2,3-*f*]benzazonines and their copper(II) complexes as microtubule destabilizing agents

Christopher Wittmann,[a] Orsolya Dömötör,[b] Irina Kuznetcova,[a] Gabriella Spengler,[b,c] Jóhannes Reynisson,[d] Lauren Holder,[d] Gavin J. Miller,[d] Eva A. Enyedy,[b] Ruoli Bai,[e] Ernest Hamel[e] and Vladimir B. Arion[a]*

[a] Institute of Inorganic Chemistry, University of Vienna, Währinger Strasse 42, A-1090 Vienna, Austria

[b] MTA-SZTE Lendület Functional Metal Complexes Research Group, Department of Inorganic and Analytical Chemistry, University of Szeged, Dóm tér 7, H-6720 Szeged, Hungary

[c] Department of Medical Microbiology, Albert Szent-Györgyi Health Center and Albert Szent-Györgyi Medical School, University of Szeged, Semmelweis u. 6, H-6725 Szeged, Hungary

[d] School of Chemical Sciences and School of Pharmacy and Bioengineering, Keele University, Staffordshire, ST5 5BG, United Kingdom

[e] Molecular Pharmacology Branch, Developmental Therapeutics Program, Division of Cancer Diagnosis and Treatment, National Cancer Institute, Frederick National Laboratory for Cancer Research, National Institutes of Health, Frederick, Maryland 21702, United States

Contents

- NMR atom numbering schemes S4
- Synthesis of 3-(2-iodophenyl)propanamine S4
  - 2-(2-iodophenyl)ethanol (**X1**) S4
  - 2-(2-iodophenyl)ethyl methanesulfonate (**X2**) S4
  - 3-(2-iodophenyl)propionitrile (**X3**) S5
  - 3-(2-iodophenyl)propanamine (**X4**) S5
- Synthesis of Building Blocks VIIa, VIIb, VIIc and VIId S5
  - *N*-(2-iodophenethyl)-5-bromo-1-(ethoxymethyl)-1*H*-indole-2-carboxamide (**IIb** in Scheme 1) S5
  - *N*-(2-iodophenylpropyl)-5-bromo-1-(ethoxymethyl)-1*H*-indole-2-carboxamide (**IId** in Scheme 1) S6
  - *tert*-Butyl (2-iodophenethyl)(1-(ethoxymethyl)-1*H*-indole-2-carbonyl)carbamate (**IIIb** in Scheme 1) S6
  - *tert*-Butyl (2-iodophenylpropyl)(1-(ethoxymethyl)-1*H*-indole-2-carbonyl)carbamate (**IIId** in Scheme 1) S7
  - *tert*-Butyl 12-bromo-9-(ethoxymethyl)-5,6-dihydroindolo[2,3-*e*][3]benzazocin-8-one (**IVb** in Scheme 1) S7
  - *tert*-Butyl 13-bromo-10-(ethoxymethyl)-5,6-dihydroindolo[2,3-*e*][4]benzazonin-9-one (**IVd** in Scheme 1) S8
  - 12-Bromo-5,6,7,9-tetrahydroindolo[2,3-*e*][3]benzazocin-8(7*H*)-one (**Vb** in Scheme 1) S8
  - 13-Bromo-5,6,7,10-tetrahydroindolo[2,3-*f*][4]benzazonin-9(8*H*)-one (**Vd** in Scheme 1) S9
  - 5,6,7,9-tetrahydroindolo[2,3-*e*]benzazocin-8-thione (**VIa** in Scheme 2) S9
  - 12-Bromo-5,6,7,9-tetrahydroindolo[2,3-*e*]benzazocin-8-thione (**VIb** in Scheme 2) S9
  - 5,6,7,10-tetrahydroindolo[2,3-*f*]benzazonin-9-thione (**VIc** in Scheme 2) S10
  - 13-Bromo-5,6,7,10-tetrahydroindolo[2,3-*f*]benzazonin-9-thione (**VId** in Scheme 2) S10
  - 8-Hydrazin-yl-5,6,7,9-tetrahydroindolo[2,3-*e*]benzazocine (**VIIa** in Scheme 2) S10
  - 12-Bromo-8-hydrazin-yl-5,6,7,9-tetrahydroindolo[2,3-*e*]benzazocine (**VIIb** in Scheme 2) S11
  - 9-Hydrazin-yl-5,6,7,10-tetrahydroindolo[2,3-*f*]benzazonine (**VIIc** in Scheme 2) S11
  - 13-Bromo-9-hydrazin-yl-5,6,7,10-tetrahydroindolo[2,3-*f*]benzazonine (**VIId** in Scheme 2) S11

- Structural Conformation Details S12
- Molecular Docking Calculations S13
- Yields, Elemental Analysis and ESI MS data S17
- Additional X-ray Diffraction Data S20
- Crystallographic Data Collection S24
- UV–vis data S26
- NMR spectra S29
- ESI mass spectra S45
- References S71

- NMR atom numbering schemes

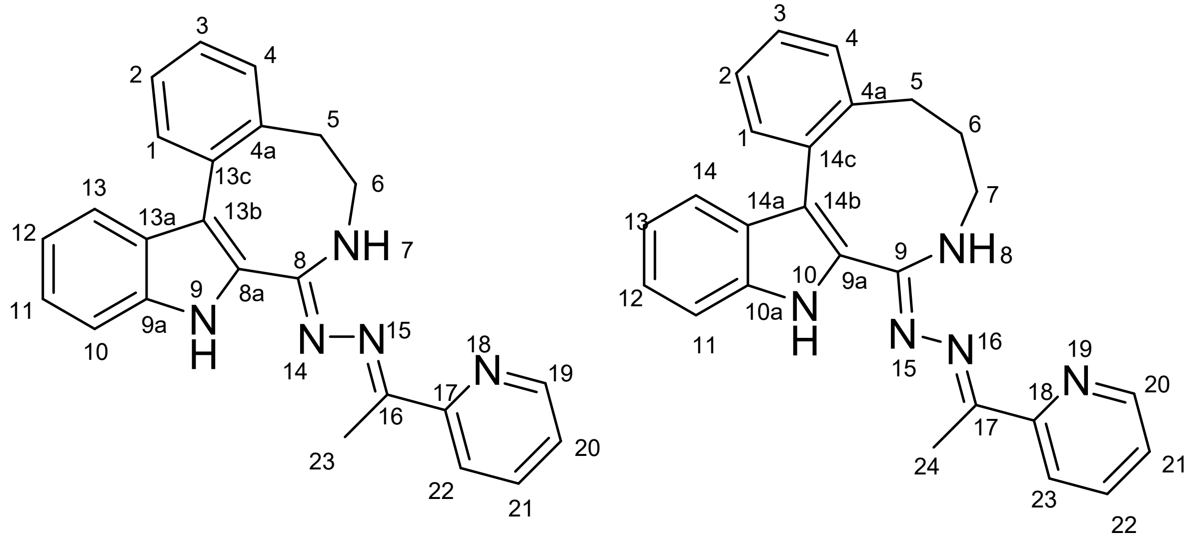

Scheme S1. Atom numbering schemes for ligands based on indolo[2,3-*e*][3]benzazocin-8(7*H*)-ones (left) and indolo[2,3-*f*][4]benzazonin-9(8*H*)ones (right).

- Synthesis of 3-(2-iodophenyl)propanamine

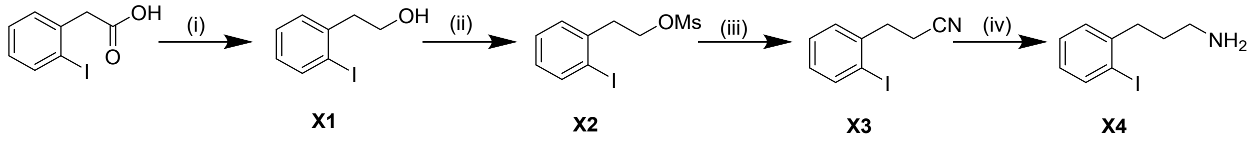

Scheme S2. Synthesis of 3-(2-iodophenyl)propan-1-amine **X4**. Reagents and conditions: (i): DIBAL-H, THF_{dry}, −80 °C - rt, 1 h; (ii): MsCl, NEt_3, DCM_{dry}, 0 °C - rt, 2 h; (iii): KCN, DMSO, 60 °C, 18 h; (iv): $AlCl_3$, $LiAlH_4$, Et_2O_{abs}, −21 °C - 0 °C, 1h.

- *2-(2-iodophenyl)ethanol (**X1**)*

  Under argon atmosphere, 2-iodophenyl acetic acid (5 g, 19.08 mmol) was dissolved in dry THF (240 mL). The solution was cooled to −80 °C and DIBAL-H (52.5 mL, 1.2 M in toluene) was added dropwise. The reaction mixture was stirred at room temperature for 1 h. After cooling to −21 °C 1 M NaOH (25 mL) was added. The THF fraction was dried over magnesium sulfate and concentrated *in vacuo*. The crude oil was purified on silica by using hexane : ethyl acetate 7:3 to give a colourless oil. Yield: 4.48 g. 95%. ^{1}H NMR (500 MHz, DMSO-d_6) δ 7.81 (d, J = 7.8 Hz, 1H), 7.34 – 7.30 (m, 2H), 6.98 – 6.92 (m, 1H), 4.75 (t, J = 5.2 Hz, 1H), 3.56 (td, J = 7.2, 5.3 Hz, 2H), 2.83 (t, J = 7.2 Hz, 2H).

- *2-(2-iodophenyl)ethyl methanesulfonate (**X2**)*

  Under argon atmosphere, to a solution of **X1** (4.48 g, 18.05 mmol) in dry DCM (60 mL) cooled to 0 °C, triethylamine (3.7 mL, 27.07 mmol) was added. After 20 min

mesyl chloride (1.67 mL, 21.66 mmol) was added carefully, the resulting suspension stirred at room temperature for 2 h and then poured onto ice (80 g). The organic phase was separated and washed with saturated sodium hydrogencarbonate solution (100 mL). The DCM phase was dried over magnesium sulfate and concentrated *in vacuo* to give a colourless oil. Yield. 5.88 g. 99%. ^{1}H NMR (500 MHz, DMSO-d_6) δ 7.87 (d, J = 7.8 Hz, 1H), 7.38 (dd, J = 7.8, 5.4 Hz, 2H), 7.05 – 6.99 (m, 1H), 4.38 (t, J = 6.9 Hz, 2H), 3.14 (s, 3H), 3.11 (t, J = 6.9 Hz, 2H).

- *3-(2-iodophenyl)propionitrile (**X3**)*
  To a solution of **X2** (5.07g, 15.5 mmol) in DMSO (60 mL) at 60 °C KCN (2.00 g, 31 mmol) was added, and the resulting suspension was stirred at 60 °C overnight. The suspension was poured into saturated $NaHCO_3$ solution (100 mL) and extracted with DCM (2 × 100 mL). The combined organic phases were washed with water (150 mL) and dried over magnesium sulfate. The crude oil was purified on silica by using hexane : ethyl acetate 9:1 to give a colourless oil. Yield: 3.41 g. 85%. ^{1}H NMR (500 MHz, DMSO-d_6) δ 7.87 (d, J = 7.8 Hz, 1H), 7.42 – 7.36 (m, 2H), 7.06 – 6.99 (m, 1H), 2.98 (t, J = 7.3 Hz, 2H), 2.80 (t, J = 7.3 Hz, 2H).

- *3-(2-iodophenyl)propanamine (**X4**)*
  Under argon atmosphere, at −21 °C $AlCl_3$ (1.85 g, 13.84 mmol) and $LiAlH_4$ (18 mL, 1 M in THF, 1.3 eq) were carefully suspended in freshly distilled Et_2O (60 mL). A solution of **X3** (3.56 g, 13.8 mmol) in freshly distilled Et_2O (30 mL) was added dropwise to the suspension. The mixture was stirred at 0 °C for 1 h and quenched carefully with 4 M NaOH (16 mL). The resulting suspension was dried over magnesium sulfate and concentrated *in vacuo* to give a colourless oil. Yield: 2.91 g, 80 %.^{1}H NMR (500 MHz, DMSO-d_6) δ 7.85 – 7.76 (m, 1H), 7.37 – 7.25 (m, 2H), 6.92 (tt, J = 26.0, 13.0 Hz, 1H), 2.66 (dd, J = 17.0, 9.1 Hz, 2H), 2.57 (t, J = 6.9 Hz, 2H), 1.63 – 1.54 (m, 2H), two proton signals are likely overlapping with solvent molecules. ESI-MS (acetonitrile/methanol + 1% water), positive: m/z 261.98 $[M + H]^+$ (calcd m/z for $[C_9H_{12}NI + H]^+$ 262.01).

- Synthesis of Building Blocks VIIa, VIIb, VIIc and VIId

Va and carboxylic acids **Ia** and **Ib** were prepared according to literature protocols.[1] Herein 2-(2-bromophenyl)ethyl-1-amine was used instead of 2-(2-iodophenyl)ethyl-1-amine, and this resulted in no changes.

- *N-(2-iodophenethyl)-5-bromo-1-(ethoxymethyl)-1H-indole-2-carboxamide (**IIb** in Scheme 1)*

Under argon atmosphere, to a solution of 2-(2-iodophenyl)ethan-1-amine (3.6 g, 14.6 mmol) in DCM (150 mL) cooled to 0 °C **Ib** (4.32 g, 13.2 mmol) was added, followed by EDCI·HCl (2.79 g, 14.6 mmol) and DMAP (1.62 g, 13.2 mmol). The reaction mixture was stirred for at 0 °C for 4

h and at room temperature overnight. Then water (40 mL) was added, and the solution was acidified with 6 M HCl to pH 1. After separation of the phases, the product was extracted with DCM (2 × 40 mL). The combined organic phases were dried over magnesium sulfate and concentrated *in vacuo*. The remainder was washed with ice cold ether (40 mL) to give a white solid. Yield: 6.54 g, 94%. ^{1}H NMR (500 MHz, DMSO-d_6) δ 8.79 (t, J = 5.7 Hz, 1H), 7.90 (d, J = 1.9 Hz, 1H), 7.85 (d, J = 7.6 Hz, 1H), 7.61 (d, J = 8.8 Hz, 1H), 7.41 (dd, J = 8.8, 2.0 Hz, 1H), 7.36 – 7.30 (m, 2H), 7.06 (s, 1H), 7.01 – 6.95 (m, 1H), 5.95 (s, 2H), 3.50 (dd, J = 13.0, 6.9 Hz, 2H), 3.31 (d, J = 7.0 Hz, 2H), 2.97 (dd, J = 13.3, 6.2 Hz, 2H), 0.98 (t, J = 7.0 Hz, 3H).

- *N-(2-iodophenylpropyl)-5-bromo-1-(ethoxymethyl)-1H-indole-2-carboxamide* ***(IId** in Scheme 1)*

Under argon atmosphere, to a solution of **X4** (2.9 g, 11.1 mmol) in DCM (75 mL) cooled to 0 °C **Ib** (2.52 g, 10.1 mmol) was added, followed by EDCI·HCl (2.14 g, 11.1 mmol) and DMAP (1.24 g, 10.1 mmol). The reaction mixture was stirred at 0 °C for 4 h and at room temperature overnight. Then water (45 mL) was added, and the solution was acidified with 6 M HCl to pH 1. After the separation of the phases, the product was extracted with DCM (2 × 50 mL). The combined organic phases were dried over magnesium sulfate and concentrated *in vacuo*. The remainder was washed with ice cold diethyl ether (40 mL) to give a white solid. Yield: 4.39 g, 80%. ^{1}H NMR (500 MHz, DMSO-d_6) δ 8.76 (t, J = 5.5 Hz, 1H), 7.90 (d, J = 1.8 Hz, 1H), 7.83 (d, J = 7.8 Hz, 1H), 7.61 (d, J = 8.8 Hz, 1H), 7.40 (dd, J = 8.8, 1.9 Hz, 1H), 7.37 – 7.32 (m, 2H), 7.09 (s, 1H), 6.98 – 6.92 (m, 1H), 5.96 (s, 2H), 2.76 – 2.70 (m, 2H), 1.83 – 1.75 (m, 2H), 0.98 (t, J = 7.0 Hz, 3H), Four proton signals are likely overlapping with solvent molecules. ESI-MS (acetonitrile/methanol + 1% water), negative: m/z 541.00 [M−H]$^{-}$ (calcd m/z for $[C_{21}H_{21}BrIN_2O_2]^{-}$ 540.96).

- *tert-Butyl (2-iodophenethyl)(1-(ethoxymethyl)-1H-indole-2-carbonyl)carbamate* ***(IIIb** in Scheme 1)*

Under argon atmosphere, to a solution of **IIb** (6.54 g, 12.4 mmol) in dry acetonitrile (120 mL) Boc_2O (4.33 g, 19.8 mmol) and a catalytic amount of DMAP were added. The orange solution was stirred at room temperature for 18 h. The solvent was evaporated and the residue partitioned between water and DCM 1:1 (80 mL). The aqueous layer was further extracted with DCM (2 × 80 mL). The combined organic layers were dried over magnesium sulfate. The product was purified on silica by using hexane/ethyl acetate 85:15 as eluent to give a viscous oil. Yield: 7.54 g, 97%.

^1H NMR (500 MHz, DMSO-d_6) δ 7.87 (d, J = 1.8 Hz, 1H), 7.83 (dd, J = 7.8, 0.7 Hz, 1H), 7.65 (d, J = 8.9 Hz, 1H), 7.43 (dd, J = 8.8, 1.9 Hz, 1H), 7.37 – 7.31 (m, 1H), 7.29 (dd, J = 7.6, 1.5 Hz, 1H), 6.98 (td, J = 7.7, 1.7 Hz, 1H), 6.74 (s, 1H), 5.67 (s, 2H), 3.99 (t, J = 7.1 Hz, 2H), 3.43 – 3.38 (m, 2H), 3.09 (t, J = 6.9 Hz, 2H), 1.08 – 1.02 (m, 12H). ESI-MS (acetonitrile/methanol + 1% water), positive: *m*/*z* 651.07 $[M+Na]^+$ (calcd *m*/*z* for $[C_{25}H_{28}BrIN_2NaO_4]^+$ 651.02).

- *tert-Butyl (2-iodophenylpropyl)(1-(ethoxymethyl)-1H-indole-2-carbonyl)carbamate (**IIId** in Scheme 1)*

Under argon atmosphere, to a solution of **IId** (1.68 g, 3.1 mmol) in dry acetonitrile (30 mL) Boc_2O (1.08 g, 5 mmol) and a catalytic amount of DMAP were added. The orange solution was stirred at room temperature for 18 h. The solvent was evaporated and the remainder partitioned between water and DCM (100 mL each). The aqueous layer was further extracted with DCM (2 × 50 mL). The combined organic layers were dried over magnesium sulfate. The product was purified on silica by using hexane/ethyl acetate 88:12 as eluent to give a viscous oil. Yield: 1.84 g, 92%. ^1H NMR (500 MHz, DMSO-d_6) δ 7.90 (d, J = 1.9 Hz, 1H), 7.83 (d, J = 7.7 Hz, 1H), 7.66 (d, J = 8.9 Hz, 1H), 7.44 (dd, J = 8.8, 2.0 Hz, 1H), 7.37 – 7.34 (m, 2H), 6.97 (ddd, J = 8.0, 5.6, 3.5 Hz, 1H), 6.87 (s, 1H), 5.70 (s, 2H), 3.80 (t, J = 7.1 Hz, 2H), 3.42 (q, J = 7.0 Hz, 2H), 2.78 – 2.71 (m, 2H), 1.87 (dt, J = 15.1, 7.7 Hz, 2H), 1.16 (dd, J = 14.5, 7.4 Hz, 3H), 1.14 (s, 9H). ESI-MS (acetonitrile/methanol + 1% water), positive: *m*/*z* 665.08 $[M+Na]^+$ (calcd *m*/*z* for $[C_{26}H_{30}BrIN_2NaO_4]^+$ 665.03).

- *tert-Butyl 12-bromo-9-(ethoxymethyl)-5,6-dihydroindolo[2,3-e][3]benzazocin-8-one (**IVb** in Scheme 1)*

Under argon atmosphere, to a solution of **IIIb** (7.55 g, 12.05 mmol) in dry DMF (175 mL) palladium(II) acetate (898 mg, 4 mmol), triphenylphosphine (2.1 g, 0.8 mmol) and silver(I) carbonate (8.3 g, 30.13 mmol) were added, and the suspension was stirred at 110 °C for 2 h. The solvent was removed *in vacuo*, and the black residue was taken up in DCM (50 mL). The suspension was filtered over celite and rinsed with DCM (50 mL). After evaporation of the solvent, the crude product was purified on silica by using hexane/ethyl acetate 85:15 as eluent to give a white solid. Yield: 4.1 g, 68%. ^1H NMR (500 MHz, DMSO-d_6) δ 7.83 – 7.78 (m, 1H), 7.60 – 7.56 (m, 2H), 7.42 – 7.35 (m, 4H), 5.89 (d, J = 10.9 Hz, 1H), 5.80 (d, J = 10.9 Hz, 1H), 4.10 (ddd, J = 14.6, 8.0, 6.6 Hz, 1H), 3.62 – 3.54 (m, 1H), 3.50 – 3.42 (m, 1H), 3.33 (1H, H_2O overlapped), 2.97

(ddd, J = 14.0, 8.1, 5.7 Hz, 1H), 2.79 (ddd, J = 14.4, 8.2, 6.6 Hz, 1H), 1.20 (s, 9H), 1.05 (t, J = 7.0 Hz, 3H). ESI-MS (acetonitrile/methanol + 1% water), positive: *m/z* 523.13 $[M+Na]^+$ (calcd *m/z* for $[C_{25}H_{27}BrN_2NaO_4]^+$ 523.10).

- *tert-Butyl 13-bromo-10-(ethoxymethyl)-5,6-dihydroindolo[2,3-e][4]benzazonin-9-one (**IVd** in Scheme 1)*

Under argon atmosphere, to a solution of **IIId** (5.22 g, 8.1 mmol) in dry DMF (280 mL) palladium(II) acetate (270 mg, 1.2 mmol), triphenylphosphine (640 mg, 2,44 mmol) and silver(I) carbonate (4.5 g, 16.28 mmol) were added, and the suspension was stirred at 140 °C for 2.5 h. The solvent was removed *in vacuo*, and the black residue was taken up in DCM (100 mL). The suspension was filtered over celite and rinsed with DCM (50 mL). After evaporation of the solvent, the crude product was purified on silica by using hexane/ethyl acetate 88:12 as eluent to give a white solid. Yield: 4.1 g, 68%. ^1H NMR (500 MHz, DMSO-d_6) δ 7.83 – 7.78 (m, 1H), 7.60 – 7.56 (m, 2H), 7.42 – 7.35 (m, 4H), 5.89 (d, J = 10.9 Hz, 1H), 5.80 (d, J = 10.9 Hz, 1H), 4.10 (ddd, J = 14.6, 8.0, 6.6 Hz, 1H), 3.62 – 3.54 (m, 1H), 3.50 – 3.42 (m, 1H), 3.33, (1H, H_2O overlapped), 2.97 (ddd, J = 14.0, 8.1, 5.7 Hz, 1H), 2.79 (ddd, J = 14.4, 8.2, 6.6 Hz, 1H), 1.20 (s, 9H), 1.05 (t, J = 7.0 Hz, 3H), two proton signals are likely overlapping with DMSO. ESI-MS (acetonitrile/methanol + 1% water), positive: *m/z* 537.17 $[M+Na]^+$ (calcd *m/z* for $[C_{26}H_{29}BrN_2NaO_4]^+$ 537.12).

- *12-Bromo-5,6,7,9-tetrahydroindolo[2,3-e][3]benzazocin-8(7H)-one (**Vb** in Scheme 1)*

To a solution of **IVb** (4.08 g, 8.1 mmol) in dioxane (160 mL) 1 M HCl (80 mL) was added, and the reaction mixture was stirred at 80 °C for 2 h. After cooling to room temperature, the solution was neutralized with solid $NaHCO_3$. The product was extracted with DCM (3 × 100 mL). The organic phase was afterwards dried over magnesium sulfate. The solution was evaporated *in vacuo*, and the residue was taken up in methanol (7 mL) and left stirring at 40 °C and 320 mbar for 20 min. The white precipitate was isolated by filtration and washed with diethylether. Yield: 2.13 g, 78%. ^1H NMR (500 MHz, DMSO-d_6) δ 12.01 (s, 1H), 7.74 (t, J = 3.7 Hz, 1H), 7.58 (d, J = 1.8 Hz, 1H), 7.44 – 7.39 (m, 3H), 7.39 – 7.32 (m, 3H), 3.63 (s, 1H), 3.33 (1H, water overlapped) 2.95 (d, J = 9.3 Hz, 2H). ESI-MS (acetonitrile/methanol + 1% water), positive: *m/z* 341.14 $[M+H]^+$ (calcd *m/z* for $[C_{17}H_{14}BrN_2O]^+$ 341.03).

- *13-Bromo-5,6,7,10-tetrahydroindolo[2,3-f][4]benzazonin-9(8H)-one (**Vd** in Scheme 1)*

To a solution of **IVd** (1.98 g, 4.57 mmol) in ethanol (140 mL) 12 M HCl (35 mL) was added. The solution was stirred at 100 °C for 1 h, then cooled to room temperature and neutralized with a saturated aqueous solution of $NaHCO_3$. Ethanol was removed at reduced pressure, and the aqueous suspension extracted with ethyl acetate (3 × 150 mL). The combined organic phases were washed with brine (200 mL) and dried over magnesium sulfate. After evaporation of the solvent, the crude solid was suspended in acetone (5 mL) and filtered. The filtrate was stored at 4 °C overnight generating crystals of X-ray diffraction quality.

- *5,6,7,9-tetrahydroindolo[2,3-e]benzazocin-8-thione (**VIa** in Scheme 2)*

Under argon atmosphere, to a solution of **Va** (500 mg, 1.91 mmol) in dry dioxane (50 mL) Lawesson's reagent (280 mg, 0.69 mmol) was added. The mixture was stirred at 110 °C for 4 h. The cooled solution was concentrated and the crude product purified on silica using hexane/ethyl acetate 4:1 as eluent to give a yellow solid. Yield: 375 mg, 71%. ^{1}H NMR (500 MHz, DMSO-d_6) δ 11.64 (s, 1H), 10.38 (t, J = 5.3 Hz, 1H), 7.47 (t, J = 8.2 Hz, 2H), 7.36 (d, J = 6.3 Hz, 1H), 7.33 – 7.28 (m, 3H), 7.23 (t, J = 7.6 Hz, 1H), 7.07 (t, J = 7.5 Hz, 1H), 3.88 (d, J = 5.8 Hz, 1H), 3.49 (dd, J = 13.1, 6.2 Hz, 1H), 3.19 – 3.10 (m, 1H), 3.01 (dd, J = 15.9, 7.8 Hz, 1H). ESI-MS (acetonitrile/methanol + 1% water), negative: *m/z* 277.02 $[M-H]^-$ (calcd *m/z* for $[C_{17}H_{13}N_2S]^-$ 277.08).

- *12-Bromo-5,6,7,9-tetrahydroindolo[2,3-e]benzazocin-8-thione (**VIb** in Scheme 2)*

Under argon atmosphere, to a solution of **Vb** (1.57 g, 4.6 mmol) in dry dioxane (160 mL) Lawesson's reagent (744 mg, 1.84 mmol) was added, and the resulting solution was stirred at 110 °C for 4 h. The cooled mixture was concentrated and subjected to column chromatography with hexane/ethyl acetate 4:1 as eluent to give a yellow solid. Yield: 693 mg, 42%. ^{1}H NMR (500 MHz, DMSO-d_6) δ 11.96 (s, 1H), 10.55 (s, 1H), 7.61 (s, 1H), 7.52 (d, J = 8.7 Hz, 1H), 7.45 – 7.36 (m, 5H), 3.93 (d, J = 5.5 Hz, 1H), 3.56 (dd, J = 13.5, 6.5 Hz, 1H), 3.24 – 3.17 (m, 1H), 3.11 – 3.03 (m, 1H). ESI-MS (acetonitrile/methanol + 1% water), negative: *m/z* 356.87 $[M-H]^-$ (calcd *m/z* for $[C_{17}H_{12}N_2SBr]^-$ 356.99).

- *5,6,7,10-tetrahydroindolo[2,3-f]benzazonin-9-thione (**VIc** in Scheme 2)*

Under argon atmosphere, to a solution of **Vc** (189 mg, 0.68 mmol) in dry dioxane (15 mL) Lawesson's reagent (83 mg, 0.21 mmol) was added. The mixture was stirred at 110 °C for 4 h. The cooled solution was concentrated and the crude product purified on silica using hexane/ethyl acetate 3 : 2 as eluent to give a yellowish solid. Yield: 153 mg, 77%. ^{1}H NMR (500 MHz, DMSO-d_6) δ 11.66 (s, 1H), 10.27 – 10.19 (m, 1H), 7.42 (d, J = 8.2 Hz, 1H), 7.34 – 7.29 (m, 2H), 7.18 (td, J = 7.2, 1.8 Hz, 2H), 7.11 (d, J = 7.9 Hz, 1H), 7.00 (dd, J = 12.6, 7.2 Hz, 2H), 3.45 (d, J = 13.1 Hz, 1H), 3.13 (dd, J = 21.8, 12.8 Hz, 1H), 2.78 (dd, J = 13.1, 7.0 Hz, 1H), 2.21 (t, J = 12.5 Hz, 1H), 1.95 – 1.87 (m, 1H), 1.63 (dd, J = 25.6, 12.6 Hz, 1H).

- *13-Bromo-5,6,7,10-tetrahydroindolo[2,3-f]benzazonin-9-thione (**VId** in Scheme 2)*

Under argon atmosphere, to a solution of **Vd** (945 mg, 2.66 mmol) in dry dioxane (75 mL) Lawesson's reagent (355 mg, 0.88 mmol) was added. The mixture was stirred at 110 °C for 4 h. The cooled solution was concentrated, and the crude product purified on silica using hexane/ethyl acetate 4:1 as eluent to give a yellowish solid. Yield: 379 mg, 22% calculated from **IVd**. ^{1}H NMR (500 MHz, DMSO-d_6) δ 11.93 (s, 1H), 10.34 – 10.27 (m, 1H), 7.40 (d, J = 8.6 Hz, 1H), 7.33 (d, J = 4.1 Hz, 2H), 7.30 (dd, J = 8.6, 1.8 Hz, 1H), 7.23 – 7.17 (m, 2H), 7.00 (d, J = 7.5 Hz, 1H), 3.50 – 3.40 (m, 1H), 3.11 (dd, J = 22.6, 11.6 Hz, 1H), 2.78 (dd, J = 13.0, 6.8 Hz, 1H), 2.19 (t, J = 12.7 Hz, 1H), 1.96 – 1.87 (m, 1H), 1.62 (dd, J = 25.9, 12.3 Hz, 1H). ESI-MS (acetonitrile/methanol + 1% water), negative: m/z 370.85 $[M-H]^-$ (calcd m/z for $[C_{18}H_{14}N_2S]^-$ 371.00).

- *8-Hydrazin-yl-5,6,7,9-tetrahydroindolo[2,3-e]benzazocine (**VIIa** in Scheme 2)*

To a solution of **VIa** (100 mg, 0.36 mmol) in chloroform (17 mL) hydrazine monohydrate (2.2 mL) was added, and the resulting solution was refluxed for 3 h. The cooled solution was washed thoroughly with water (2 × 15 mL), and the organic phase was dried over magnesium sulfate. The resulting yellow oil was precipitated with hexane, and the solvent was removed *in vacuo*. The product was obtained as a light-yellow solid. Yield: 81.4 mg, 82%. ^{1}H NMR (500 MHz, DMSO-d_6) δ 11.16 (s, 1H), 7.56 – 7.52 (m, 1H), 7.44 (d, J = 8.0 Hz, 1H), 7.40 (d, J = 8.1 Hz, 1H), 7.38 – 7.34 (m, 2H), 7.31 – 7.27 (m, 1H), 7.13 – 7.08 (m, 1H), 6.99 – 6.94 (m, 1H), 6.02 (s, 2H), 4.04 (t, J = 5.0 Hz, 1H), 2.95 (s, 1H), 2.70 (s, 2H), one proton signal is likely overlapping with those of solvent molecules. ESI-MS (acetonitrile/methanol + 1% water), positive: m/z 277.14 $[M+H]^+$ (calcd m/z for $[C_{17}H_{17}N_4]^+$ 277.14).

- *12-Bromo-8-hydrazin-yl-5,6,7,9-tetrahydroindolo[2,3-e]benzazocine (**VIIb** in Scheme 2)*

To a solution of **VIb** (201 mg, 0.56 mmol) in chloroform (40 mL) hydrazine monohydrate (3.5 mL) was added, and the resulting solution was refluxed for 2 h. The cooled solution was washed thoroughly with water (2 × 20 mL), and the organic phase was dried over magnesium sulfate. The resulting yellow oil was precipitated with hexane, and the solvent was removed *in vacuo*. The product was obtained as a light-yellow solid. Yield: 199 mg, 99%. ^{1}H NMR (500 MHz, DMSO-d_6) δ 11.39 (s, 1H), 8.32 (s, 1H), 7.50 (dd, *J* = 4.9, 1.5 Hz, 2H), 7.40 – 7.34 (m, 3H), 7.33 – 7.28 (m, 1H), 7.22 (dd, *J* = 8.6, 1.9 Hz, 1H), 6.13 (s, 2H), 3.37 (1H, water overlapped), 2.95 (d, *J* = 10.2 Hz, 1H), 2.74 – 2.64 (m, 2H). ESI-MS (acetonitrile/methanol + 1% water), positive: *m*/*z* 357.07 $[M+H]^+$ (calcd *m*/*z* for $[C_{17}H_{16}N_4Br]^+$ 357.06).

- *9-Hydrazin-yl-5,6,7,10-tetrahydroindolo[2,3-f]benzazonine (**VIIc** in Scheme 2)*

To a solution of **VIc** (144 mg, 0.49 mmol) in chloroform (25 mL) hydrazine monohydrate (3 mL) was added. The mixture was stirred at 85 °C for 3 h. The cooled solution was washed with water (2 × 25 mL) and dried over magnesium sulfate. The organic phase was concentrated, and the crude oil was taken up in DCM (10 mL),then hexane (5 mL) was added. The solvents were removed to give a yellowish solid. Yield: 138 mg, 97%. ^{1}H NMR (500 MHz, DMSO-d_6) δ 11.34 (s, 1H), 7.38 (d, *J* = 8.0 Hz, 1H), 7.33 (d, *J* = 6.8 Hz, 1H), 7.29 (t, *J* = 8.0 Hz, 1H), 7.23 – 7.09 (m, 4H), 6.96 (t, *J* = 7.0 Hz, 1H), 5.17 (s, 1H), 4.76 (s, 1H), 3.03 (s, 2H), 1.73 (s, 2H), three proton signals are likely overlapping with solvent resonances. ESI-MS (acetonitrile/methanol+1% water), positive: *m*/*z* 291.18 $[M+H]^+$ (calcd *m*/*z* for $[C_{18}H_{19}N_4]^+$ 291.16).

- *13-Bromo-9-hydrazin-yl-5,6,7,10-tetrahydroindolo[2,3-f]benzazonine (**VIId** in Scheme 2)*

To a solution of **VId** (379 mg, 1.02 mmol) in chloroform (64 mL) hydrazine monohydrate (5.6 mL) was added. The mixture was stirred at 85 °C for 3 h. The cooled solution was washed with water (2 × 100 mL) and dried over magnesium sulfate. The organic phase was concentrated and the crude oil was taken up in DCM (30 mL), then hexane (10 mL) was added. The solvents were removed to give a yellowish solid. Yield: 352 mg, 95%. ESI-MS (acetonitrile/methanol + 1% water), positive: *m*/*z* 369.12 $[M+H]^+$ (calcd *m*/*z* for $[C_{18}H_{18}BrN_4]^+$ 369.07).

- Structural Conformation Details

Table S1. Torsional angles in eight-membered azocine ring(s) in **VIa·HL[4], 2** and **4.**

Compound	**VIa**	**HL[4]**	**2**	**4**
$\Theta_{N7–C6–C5–C4a}$	64.6(1)	50.2(4)	66.3(2)	–48.61(18)
$\Theta_{C6–C5–C4a–C13c}$	–88.97(9)	–88.3(4)	–93.76(18)	93.15(17)
$\Theta_{C5–C4a–C13c–C13b}$	–3.55(9)	–1.5(4)	1.1(2)	–4.0(2)
$\Theta_{C4a–C13c–C13b–C8a}$	51.76(10)	53.8(4)	46.1(2)	–51.1(2)
$\Theta_{C13c–C13b–C8a–C8}$	15.12(12)	3.9(5)	14.5(3)	–12.7(2)
$\Theta_{C13b–C8a–C8–N7}$	–47.89(13)	–19.3(5)	–50.1(3)	58.1(2)
$\Theta_{C8a–C8–N7–C6}$	–19.79(14)	–61.6(4)	–12.4(3)	15.1(3)
$\Theta_{C8–N7–C6–C5}$	24.98(14)	60.7(4)	17.7(3)	–37.3(2)

- Molecular Docking Calculations

Table S2. The binding affinities as predicted by the scoring functions for the tubulin-colchicine complex.[2] CN2 is the co-crystallised ligand. Root-mean-square deviation – RMSD from the co-crystallised ligand (heavy atoms) in Å.

Complexes	GS	Ligands	GS	CS	ChemPLP	ASP
**1**	61.9	**HL[1]**	57.0	30.2	60.4	33.6
**2**	53.2	**HL[2]**	62.2	30.7	65.9	27.0
**3**	57.9	**HL[3]**	56.7	30.0	58.5	29.7
**4**	51.3	**HL[4]**	61.3	30.4	58.1	25.0
**5**	53.7	**HL[5]**	61.3	34.0	62.1	31.8
**6**	50.0	**HL[6]**	59.2	35.1	62.2	26.5
		CN2	61.9	21.6	60.1	17.4
		RMSD:	7.5155	2.8032	1.0908	7.1644

Table S3. The molecular descriptors as calculated by QikProp and their corresponding Known Drug Indexes 2a and 2b ($KDI_{2a/2b}$).

	RB	MW(g/mol)	HD	HA	Log P	PSA (Å^2)	KDI_{2A}	KDI_{2B}
**HL^1**	3	365.4	2	4	4.7	63.5	5.41	0.52
**HL^2**	3	379.5	2	3.5	5.3	59.9	5.14	0.34
**HL^3**	3	444.3	2	4	5.2	63.5	5.07	0.33
**HL^4**	3	458.4	2	3.5	5.8	59.9	4.73	0.19
**HL^5**	3	393.5	2	3.5	5.5	60.9	5.05	0.30
**HL^6**	3	472.4	2	3.5	6.1	60.9	4.60	0.14

Table S4. The molecular descriptors as calculated by Scigress.

	MW(g/mol)	HD	HA	Log *P*
**HL^1**	365.4	2	5	4.4
**HL^2**	379.5	2	5	4.1
**HL^3**	444.3	2	5	5.2
**HL^4**	458.4	2	5	4.9
**HL^5**	393.5	2	5	4.5
**HL^6**	472.4	2	5	5.2
**1**	499.9	2	5	5.4
**2**	513.9	2	5	4.7
**3**	578.8	2	5	6.2
**4**	592.8	2	5	5.5
**5**	527.9	2	5	5.1
**6**	606.8	2	5	5.9

Table S5. Definition of lead-like, drug-like and Known Drug Space (KDS) in terms of molecular descriptors. The values given are the maxima for each descriptor for the volumes of chemical space used.

	Lead-like Space	Drug-like Space	Known Drug Space
Molecular weight (g mol^{-1})	300	500	800
Lipophilicity (Log *P*)	3	5	6.5
Hydrogen bond donors (HD)	3	5	7
Hydrogen bond acceptors (HA)	3	10	15
Polar surface area ($Å^2$) (PSA)	60	140	180
Rotatable bonds (RB)	3	10	17

- Yields, Elemental Analysis and ESI MS data

Table S6. Yields, elemental analysis and ESI mass spectrometric data for ligands **HL[1]–HL[4]**.

		**HL[1]**	**HL[2]**	**HL[3]**	**HL[4]**
Yield (%)		84	81	71	58
empirical formula		$C_{23}H_{19}N_5·0.2H_2O·0.2C_2H_6O$	$C_{24}H_{21}N_5·0.3C_2H_6O$	$C_{23}H_{18}N_5Br·0.2C_2H_6O$	$C_{24}H_{20}N_5Br·0.4C_2H_6O$
M_r		378.24	393.28	453.54	476.368
C (%)	calcd	74.30	75.12	61.96	62.47
	found	74.06	75.32	61.98	62.13
H (%)	calcd	5.48	5.84	4.26	4.73
	found	5.12	5.53	4.00	4.33
N (%)	calcd	18.51	17.80	15.44	14.68
	found	18.15	17.55	15.18	14.61
O (%)	calcd				
	found				
ESI-MS	$[M + Na]^+$				
	$[M + H]^+$	366.17	380.19	444.08	458.09
X-ray		no	no	no	yes

Table S7. Yields, elemental analysis and ESI MS data for ligands **HL[5]** and **HL[6]**, and complexes **1** and **2**.

		**HL[5]**	**HL[6]**	**1**	**2**
Yield (%)		46	41	70	56
empirical formula		$C_{25}H_{23}N_5·0.75CH_2Cl_2$	$C_{25}H_{22}N_5Br·CH_2Cl_2$	$C_{23}H_{19}N_5CuCl_2$	$C_{24}H_{21}N_5CuCl_2·0.8H_2O·0.2C_3H_8O$
M_r		454.63	540.24	499.88	513.91
C (%)	calcd	67.95	56.03	55.26	54.68
	found	67.89	55.915	55.39	54.87
H (%)	calcd	5.41	4.34	3.83	4.51
	found	5.445	4.3	3.44	4.16
N (%)	calcd	15.4	12.56	14.01	12.96
	found	15.055	12.455	13.71	12.57
O (%)	calcd				
	found				
ESI-MS	$[M+H]^+$	394.22	474.17		
	$[M–HCl–Cl]^+$			427.08	441.1
X-ray		no	yes	no	yes

Table S8. Yields, elemental analysis and ESI MS data for complexes **3–6**.

		**3**	**4**	**5**	**6**
Yield (%)		92	81	58	73
empirical formula		$C_{23}H_{18}N_5BrCuCl_2\cdot 0.2C_3H_8O\cdot 0.3H_2O$	$C_{24}H_{20}N_5BrCuCl_2\cdot 0.9C_3H_8O$	$C_{25}H_{23}N_5CuCl_2\cdot 1.75H_2O$	$C_{25}H_{22}N_5BrCuCl_2\cdot C_3H_8O\cdot 0.75H_2O$
M_r		596.2	646.89	557.58	677.54
C (%)	calcd	47.54	49.57	53.67	49.42
	found	47.65	49.21	53.61	49.48
H (%)	calcd	3.41	4.23	4.77	4.66
	found	3.085	3.95	4.625	4.585
N (%)	calcd	11.74	10.82	12.51	10.29
	found	11.435	10.465	12.46	10.03
O (%)	calcd				
	found				
ESI-MS	$[M–Cl]^+$			491.16	571.01
	$[M–HCl–Cl]^+$	506.99	521.01		
X-ray		no	yes	yes	no

- Additional X-ray Diffraction Data

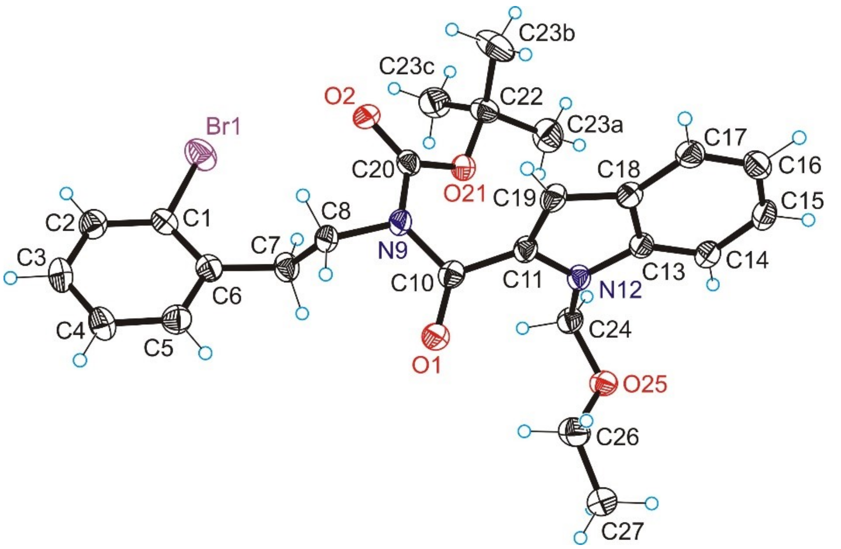

Figure S1. ORTEP view of **IIIa.**

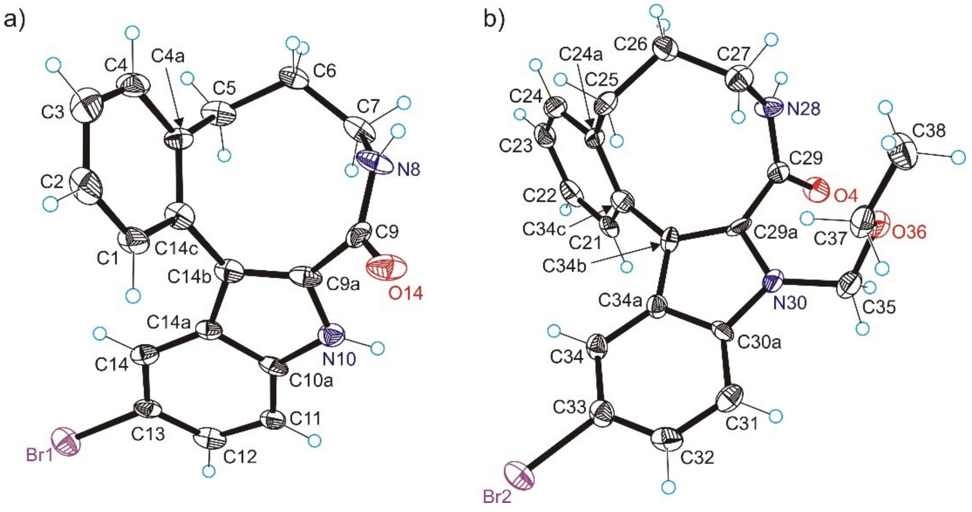

Figure S2. ORTEP view of a) **Vd** and b) **Vd^{EOM}**

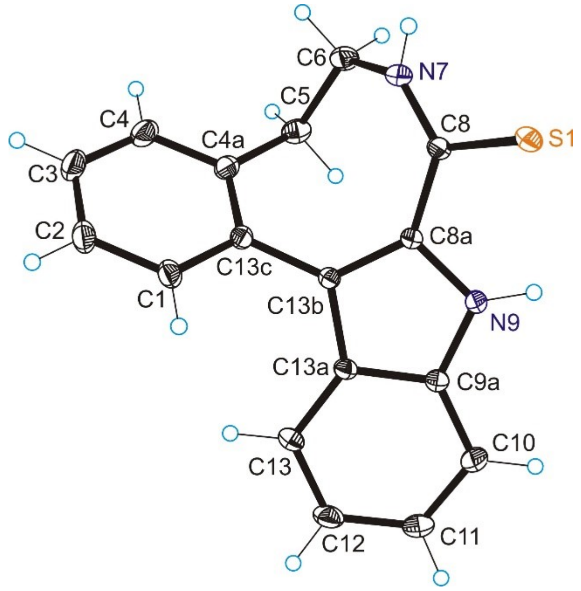

Figure S3. ORTEP view of **VIa**.

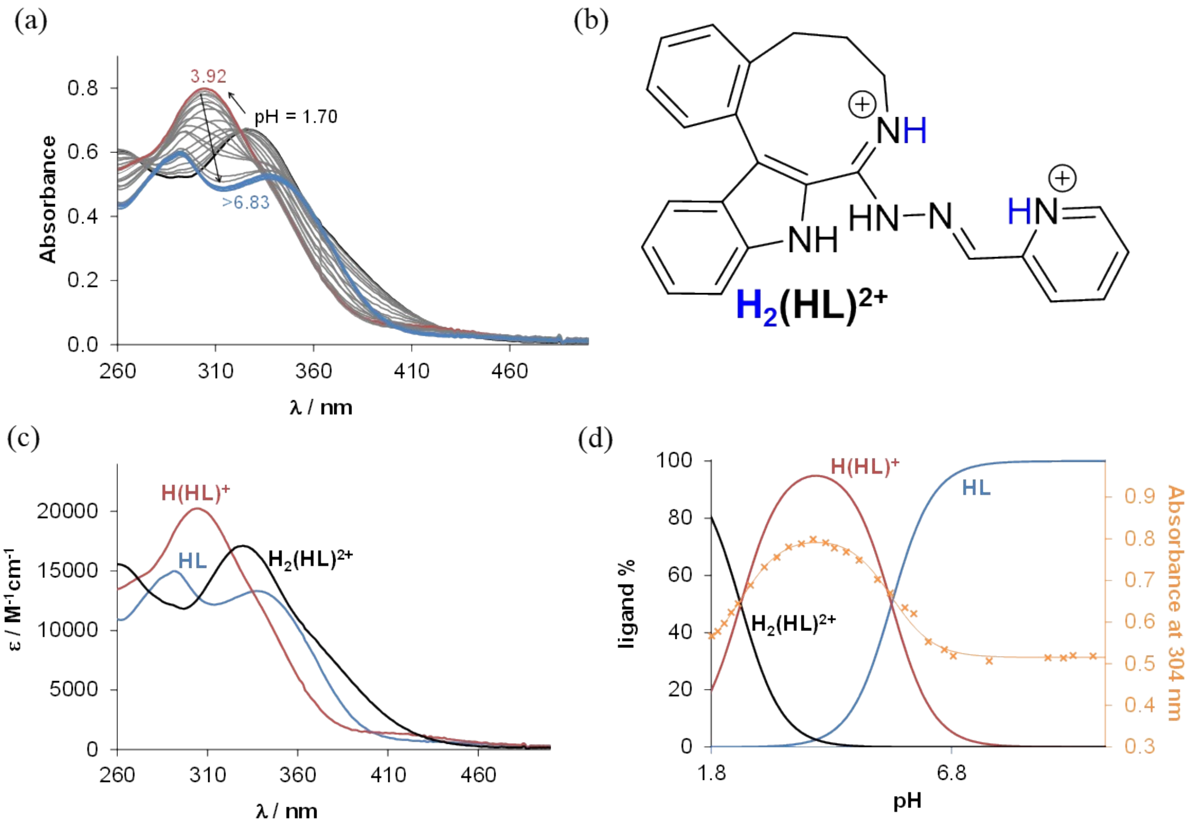

Figure S4. (a) UV–vis spectra of $\mathbf{HL^5}$ measured at various pH values; (b) The ligand in its doubly protonated form; (c) Molar absorbance spectra computed for selected ligand species in the various protonation states; (d) Concentration distribution curves and the absorbance values measured at 304 nm (×) together with the fitted line {c_{HL5} = 10 μM, T = 298 K, l = 4 cm, I = 0.10 M (KCl), 30% (v/v) DMSO/H_2O}.

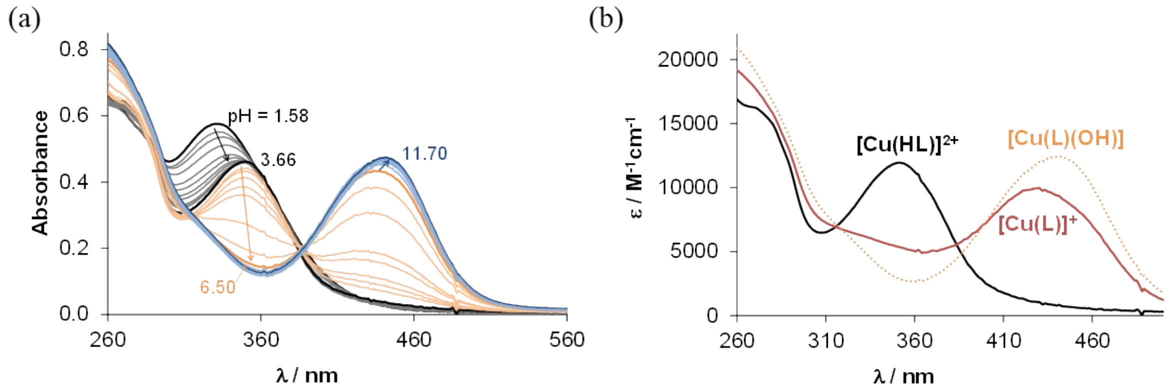

Figure S5. (a) UV–vis spectra measured for the Cu(II) – **HL**^{5} system at various pH values; (b) Molar absorbance spectra computed for selected complex species in the various protonation states {$c_{\mathbf{HL5}}$ = 10 μM, $c_{\mathrm{Cu(II)}}$ = 10 μM, T = 298 K, l = 4 cm, I = 0.10 M (KCl), 30% (v/v) DMSO/H_2O}.

- Crystallographic Data Collection

Table S9. Crystal data and details of data collection and refinement for **IIIa**, **Vd-VdMeOH**, **VIa·0.5MeOH**, **HL[4]** and **HL[6]·DCM.**

Compound	**IIIa**	**Vd-Vd^{EOM}**	**VIa·0.5MeOH**	**HL[4]**	**HL[6]·DCM**
empirical formula	$C_{25}H_{29}BrN_2O_4$	$C_{39}H_{36}Br_2N_4O_3$	$C_{17.5}H_{16}N2O_{0.5}S$	$C_{24}H_{20}BrN_5$	$C_{26}H_{24}BrCl_2N_5$
fw	501.41	768.54	294.38	458.36	557.31
space group	triclinic, $P\bar{1}$	monoclinic, $P2_1/c$	P-1	orthorhombic, $Pna2_1$	triclinic, $P\bar{1}$
a, Å	9.0922(4)	11.5688(12)	9.1130(2)	8.0799(3)	11.5481(7)
b, Å	10.7884(5)	10.8490(10)	10.6532(2)	15.5637(5)	11.5707(7)
c, Å	13.2927(7)	27.689(5)	16.7093(5)	15.6684(6)	20.2679(13)
α,°	72.853(4)	76.6520(12)	108.2046(11)		79.717(5)
β, °	81.357(4)		100.2398(10)		89.452(5)
γ, °	75.773(4)		92.5769(7)		64.211(4)
V [Å^3]	1203.45(11)	3431.1(7)	1507.65(6)	1970.35(12)	2392.2(3)
Z	2	4	4	4	4
λ [Å]	0.71073	0.71073	0.71073	0.71073	1.54178
ρ_{calcd}, g cm^{-3}	1.384	1.488	1.297	1.545	1.547
cryst size, mm^3	0.80 × 0.63 × 0.50	1.0 × 0.6 × 0.3	0.358 × 0.309 × 0.28	0.370 × 0.028 × 0.019	0.09 × 0.07 × 0.06
T [K]	100(2)	100(2)	100(2)	125(2)	100(2)
μ, mm^{-1}	1.741	2.407	0.212	2.109	1.968
R_1^a	0.0532	0.0570	0.0546	0.0480	0.0579
wR_2^b	0.1577	0.1510	0.0945	0.1227	0.1795
GOF^c	1.081	0.907	1.043	1.014	1.058

^{a} $R_1 = \Sigma||F_o| - |F_c||/\Sigma|F_o|$. ^{b} $wR_2 = \{\Sigma[w(F_o^2 - F_c^2)^2]/\Sigma[w(F_o^2)^2]\}^{1/2}$. ^{c} GOF $= \{\Sigma[w(F_o^2 - F_c^2)^2]/(n - p)\}^{1/2}$, where n is the number of reflections and p is the total number of parameters refined.

Table S10. Crystal data and details of data collection and refinement for **2·2DMF**, **4·2DMF·H$_2$O**, **5·2DMF** and **5'·i-PrOH·MeOH**.

Compound	**2·2DMF**	**4·2DMF·H$_2$O**	**5·2DMF**	**5'·i-PrOH·MeOH**
empirical formula	$C_{30}H_{35}Cl_2CuN_7O_2$	$C_{30}H_{36}BrCl_2CuN_7O_3$	$C_{31}H_{37}Cl_2CuN_7O_2$	$C_{29}H_{34}ClCuN_5O_2$
fw	660.09	757.01	674.11	583.60
space group	triclinic, $P\bar{1}$	monoclinic, $P2_1/c$	monoclinic, $C2/c$	triclinic, $P\bar{1}$
a, Å	9.8729(2)	17.514(4)	8.8925(5)	7.6320(8)
b, Å	9.9230(2)	10.420(2)	21.1262(10)	11.6124(12)
c, Å	16.4367(4)	18.168(4)	34.302(2)	16.5842(17)
α,°	77.4544(10)			90.668(3)
β, °	76.6520(12)	90.55(3)	94.643(5)	93.199(3)
γ, °	85.2866(10)			105.631(3)
V [Å^3]	1528.50(6)	3315.6(11)	6422.9(6)	1412.7(3)
Z	2	4	8	2
λ [Å]	0.71073	1.54178	1.54178	0.71073
ρ_{calcd}, g cm^{-3}	1.434	1.517	1.394	1.372
cryst size, mm^3	0.41 × 0.12 × 0.03	0.14 × 0.12 × 0.07	0.50 × 0.03 × 0.02	1.0 × 0.10 × 0.05
T [K]	140(2)	100(2)	100(2)	200(2)
μ, mm^{-1}	1.741	4.173	2.812	0.903
$R_1{}^a$	0.0402	0.0256	0.0751	0.0379
$wR_2{}^b$	0.0945	0.0671	0.2103	0.0966
GOF^c	1.021	1.044	1.024	1.039

[a] $R_1 = \Sigma||F_o| - |F_c||/\Sigma|F_o|$. [b] $wR_2 = \{\Sigma[w(F_o^2 - F_c^2)^2]/\Sigma[w(F_o^2)^2]\}^{1/2}$. [c] GOF $= \{\Sigma[w(F_o^2 - F_c^2)^2]/(n - p)\}^{1/2}$, where n is the number of reflections and p is the total number of parameters refined.

- UV−vis data

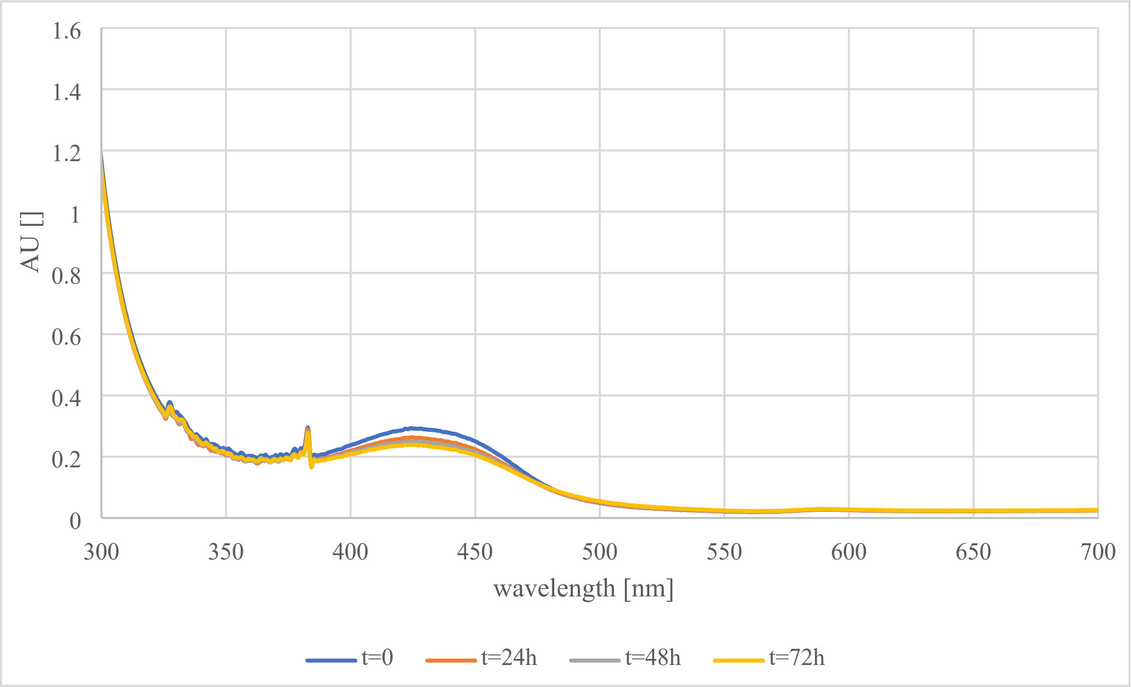

Figure S6. UV−vis stability measurement of **1** over 72 h, c = 30 µM in DMSO/H_2O 1/99.

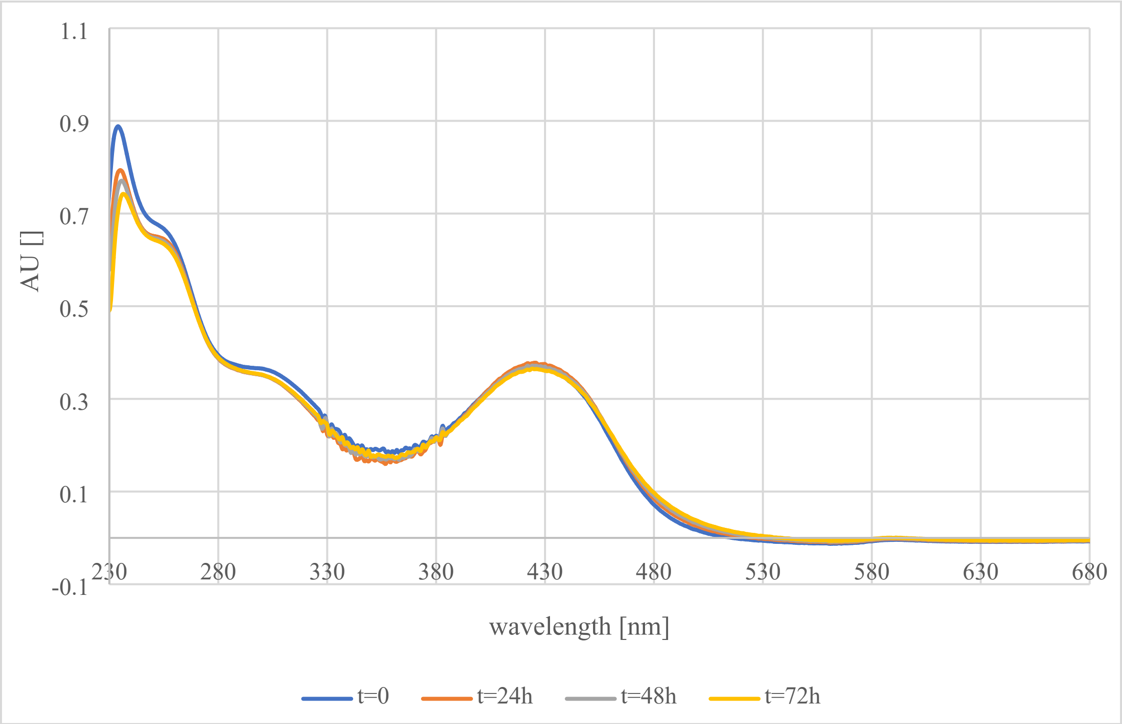

Figure S7. UV−vis stability measurement of **2** over 72 h, c = 30 µM in DMSO/H_2O 1/99.

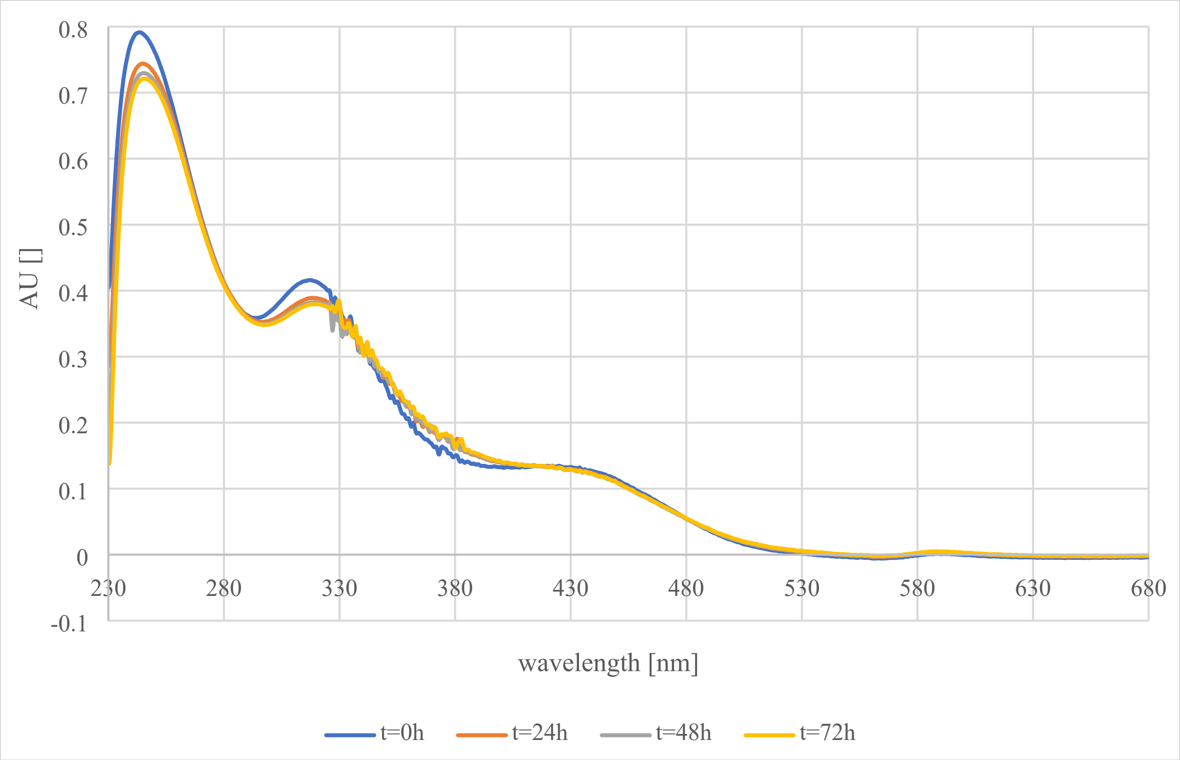

Figure S8. UV−vis stability measurement of **3** over 72 h, c = 30 μM in DMSO/H_2O 1/99.

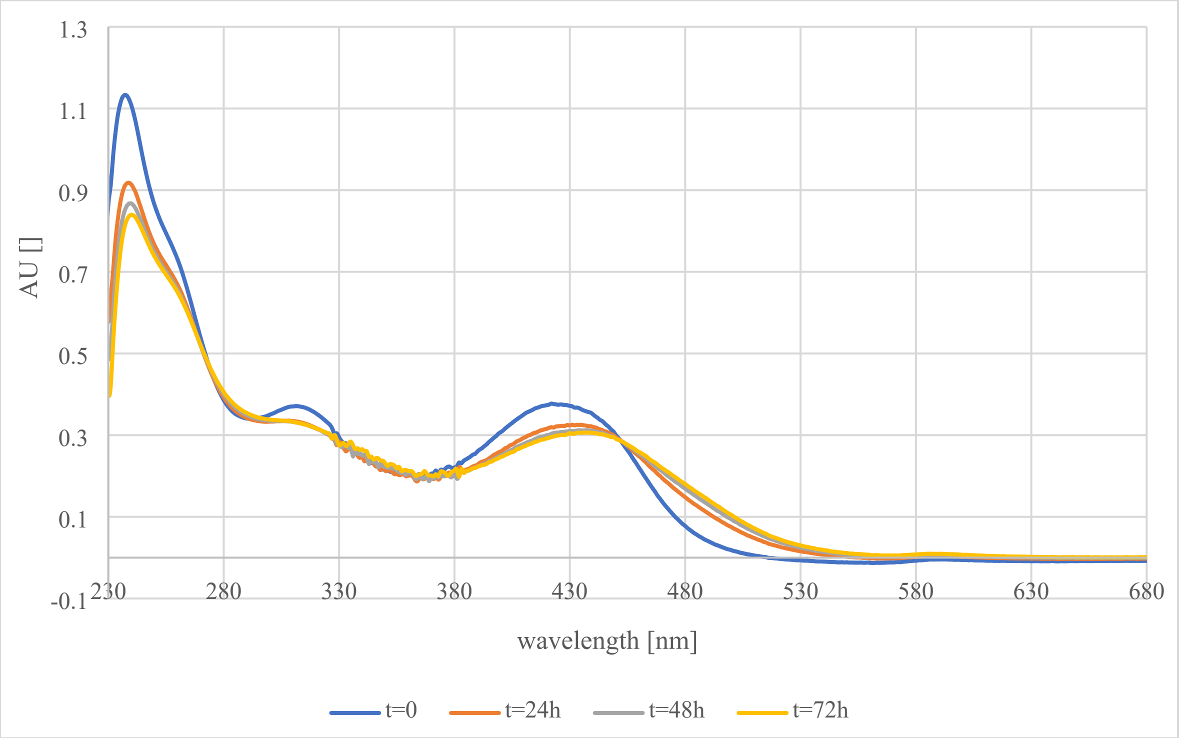

Figure S9. UV−vis stability measurement of **4** over 72 h, c = 30 μM in DMSO/H_2O 1/99.

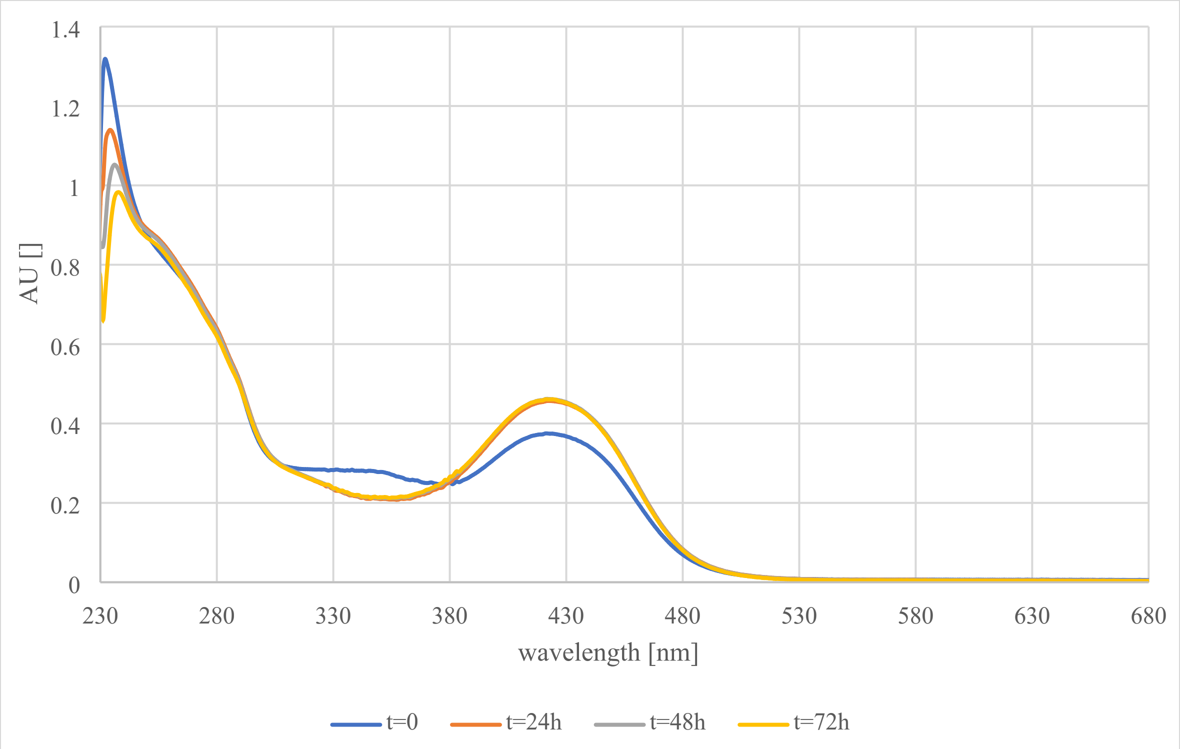

Figure S10. UV−vis stability measurement of **5** over 72 h, c = 30 µM in DMSO/H_2O 1/99.

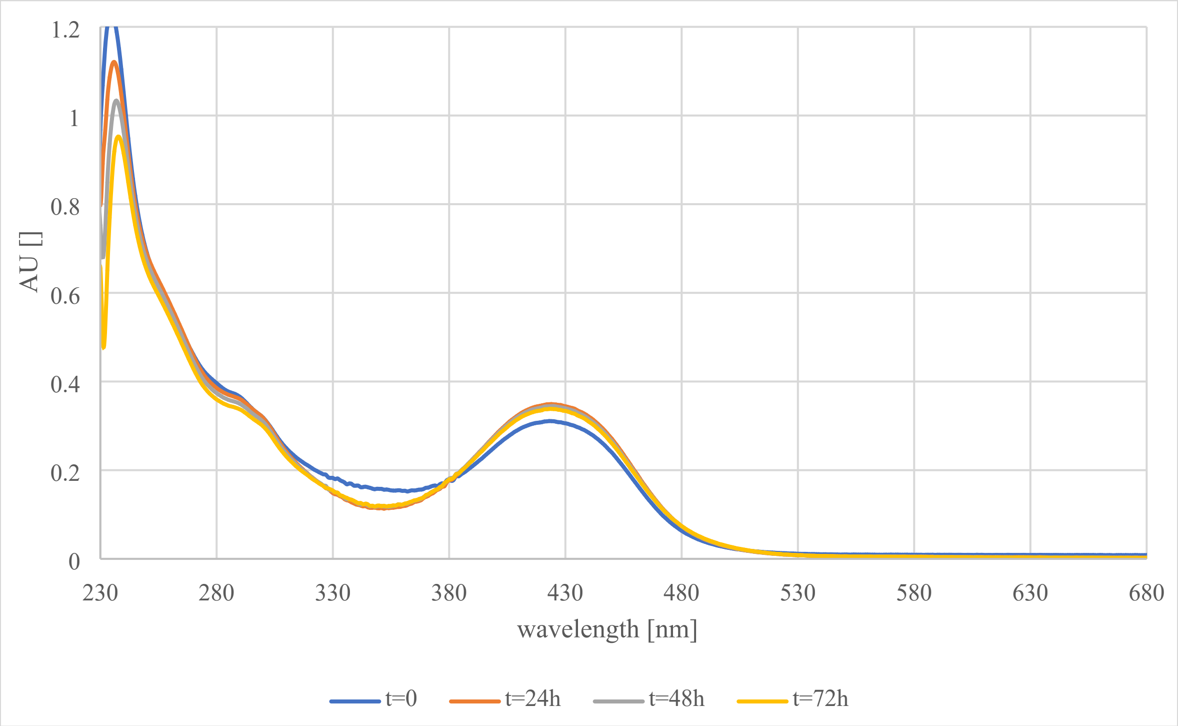

Figure S11. UV−vis stability measurement of **6** over 72 h, c = 30 µM in DMSO/H_2O 1/99.

- NMR spectra

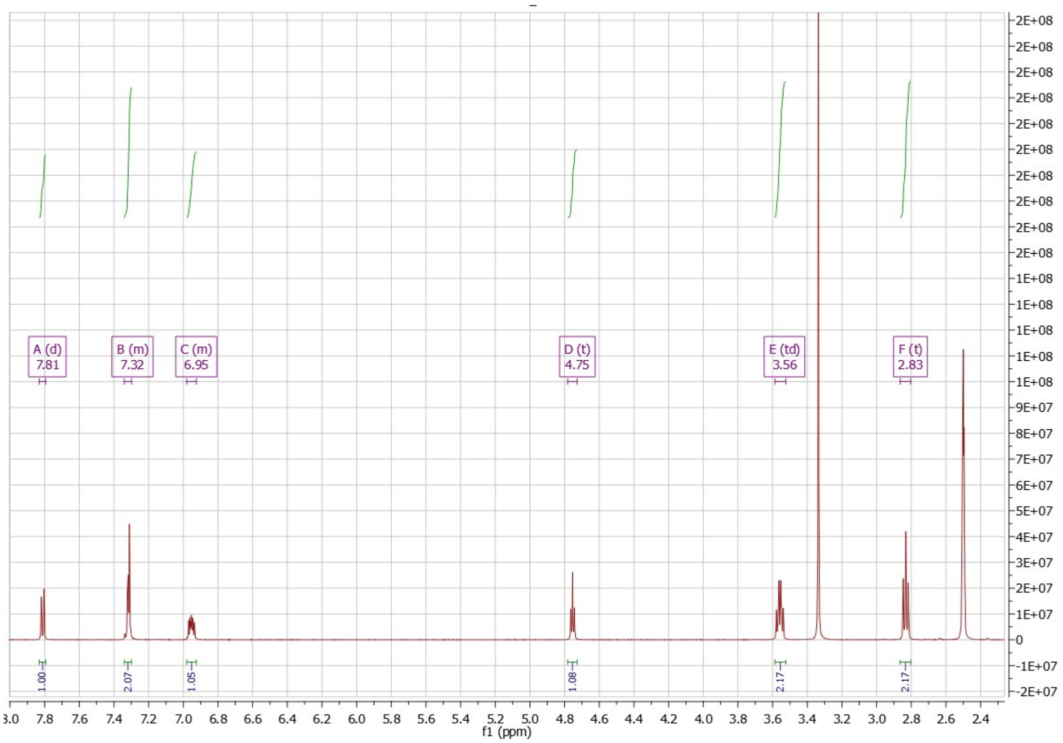

Figure S12. ^{1}H NMR spectrum of **X1** in DMSO-d_6.

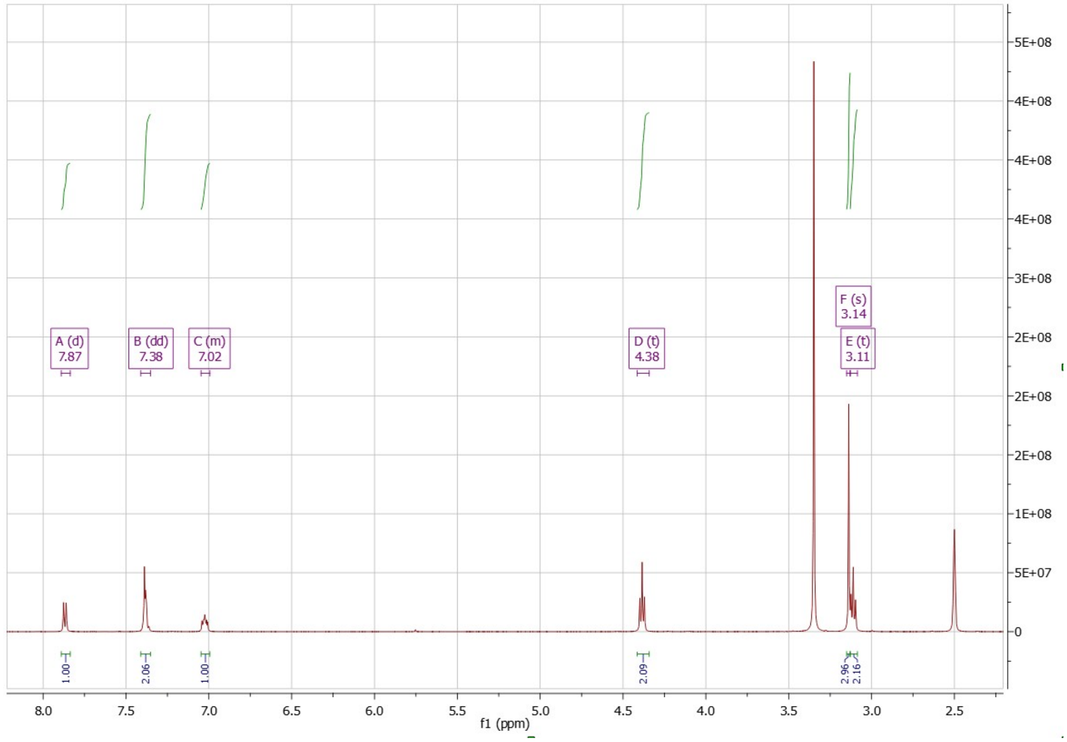

Figure S13. ^{1}H NMR spectrum of **X2** in DMSO-d_6.

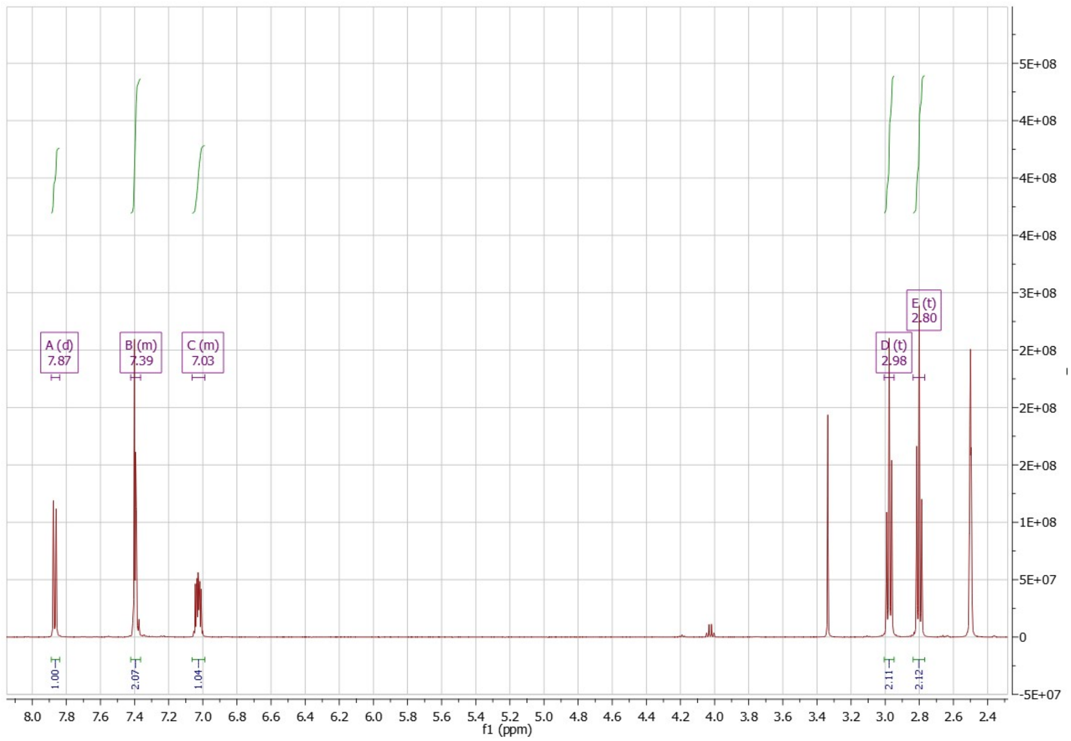

Figure S14. ^{1}H NMR spectrum of **X3** in DMSO-d_6.

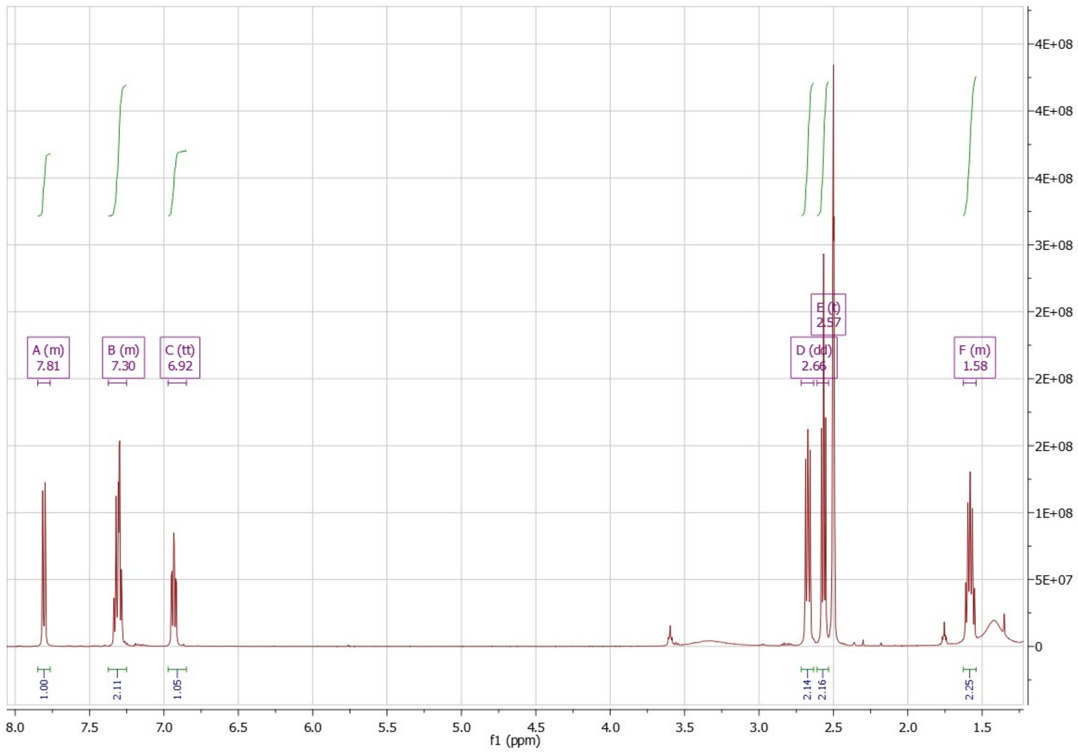

Figure S15. ^{1}H NMR spectrum of **X4** in DMSO-d_6.

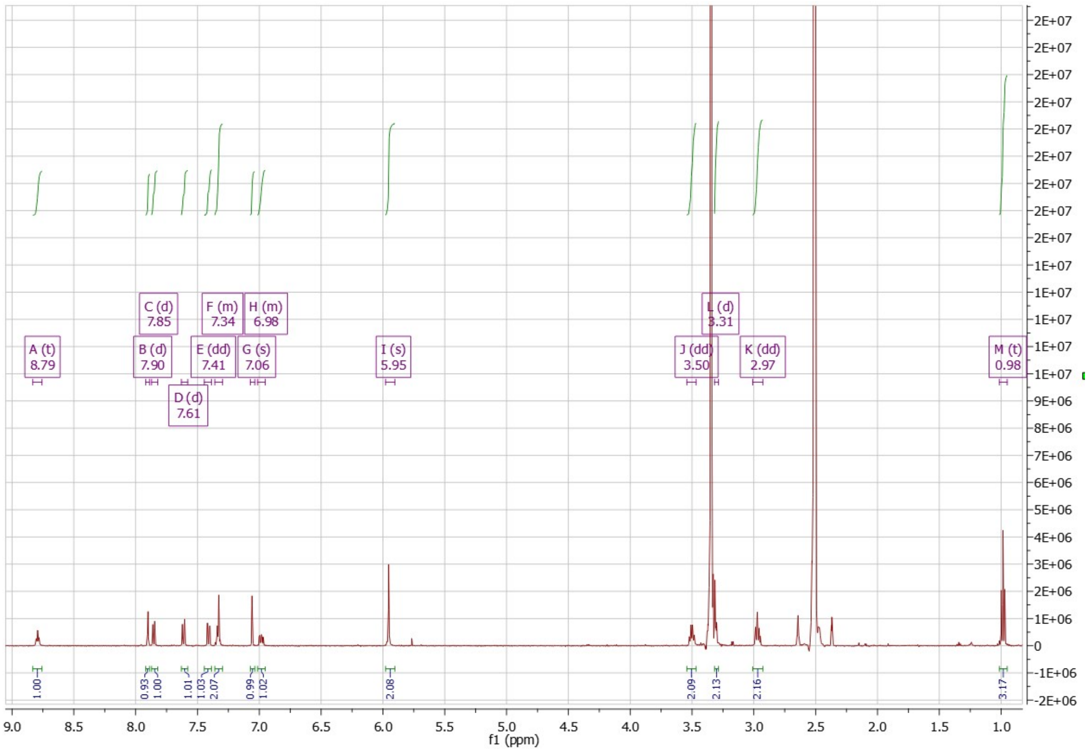

Figure S16. ^{1}H NMR spectrum of **IIb** in DMSO-d_6.

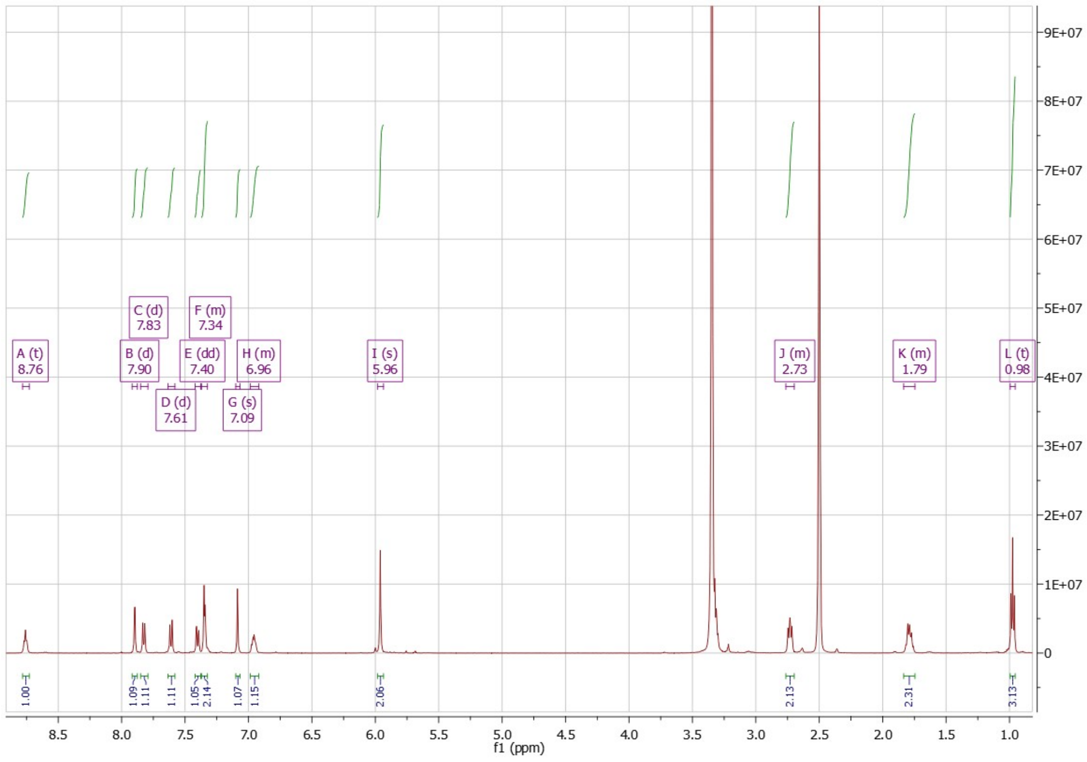

Figure S17. ^{1}H NMR spectrum of **IId** in DMSO-d_6.

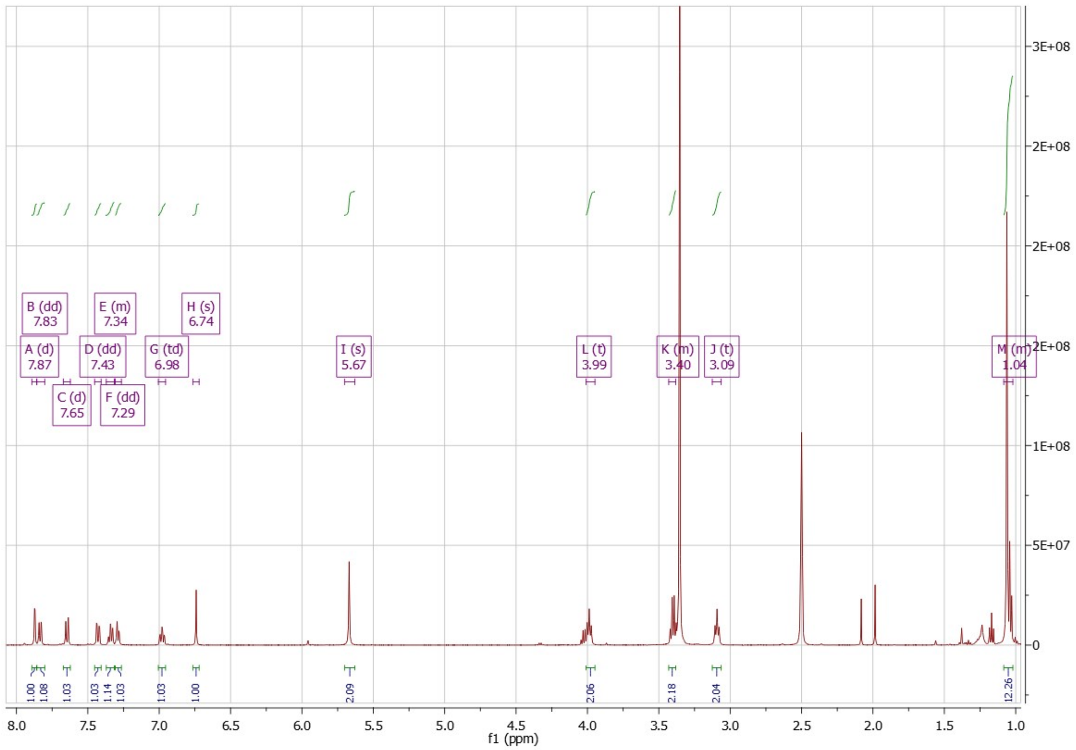

Figure S18. ^{1}H NMR spectrum of **IIIb** in DMSO-d_6.

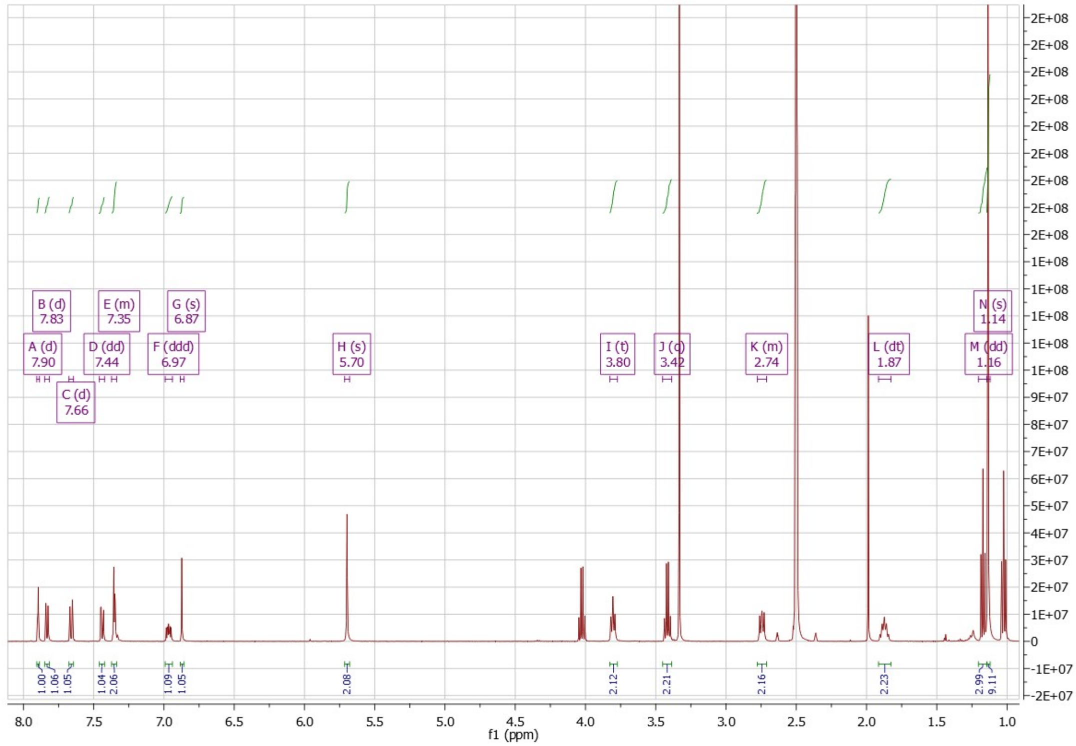

Figure S19. ^{1}H NMR spectrum of **IIId** in DMSO-d_6.

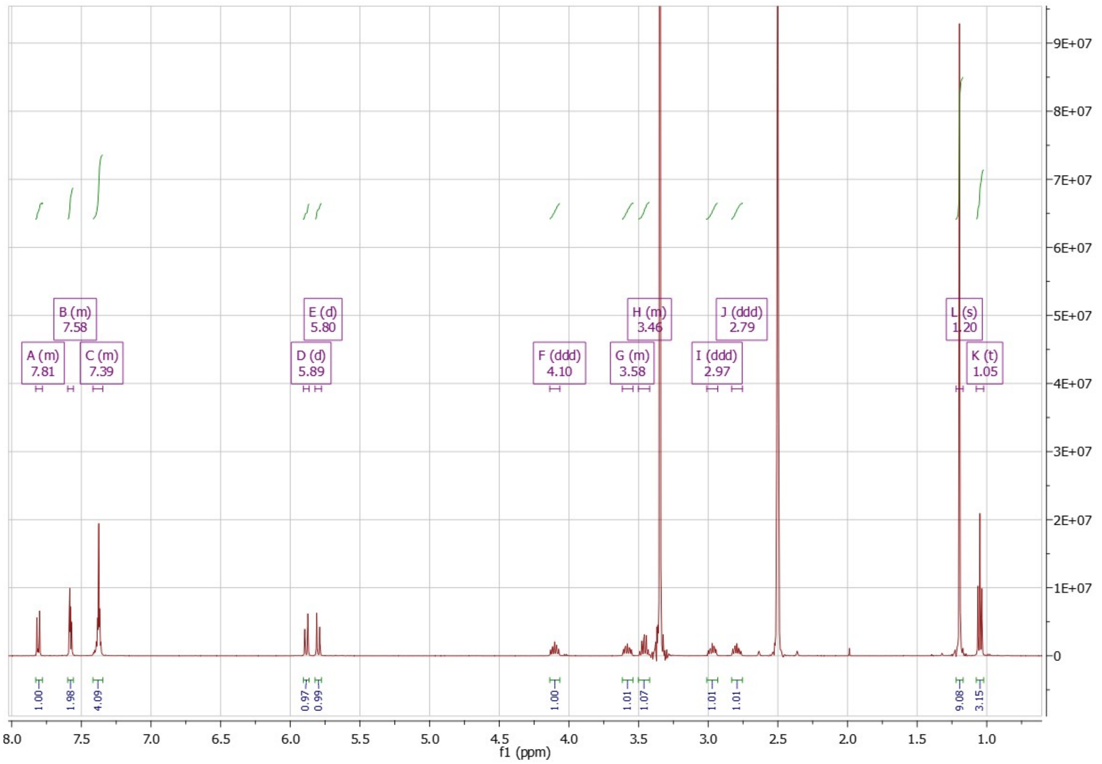

Figure S20. ^{1}H NMR spectrum of **IVb** in DMSO-d_6.

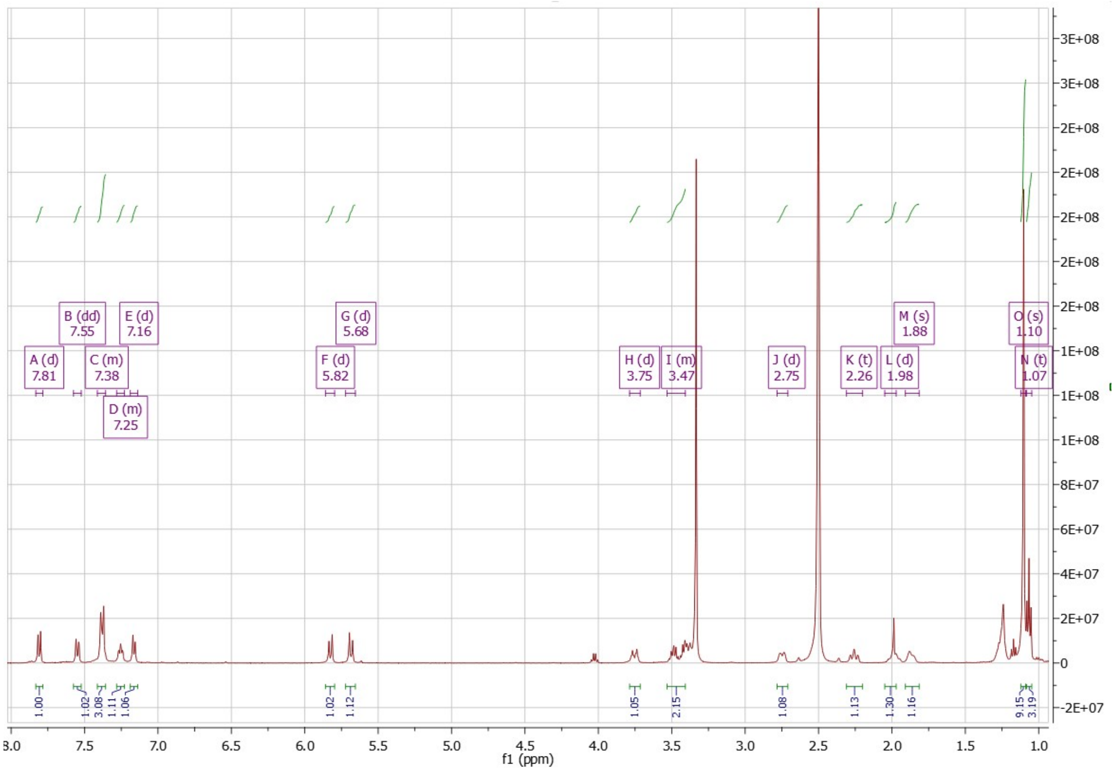

Figure S21. ^{1}H NMR spectrum of **IVd** in DMSO-d_6.

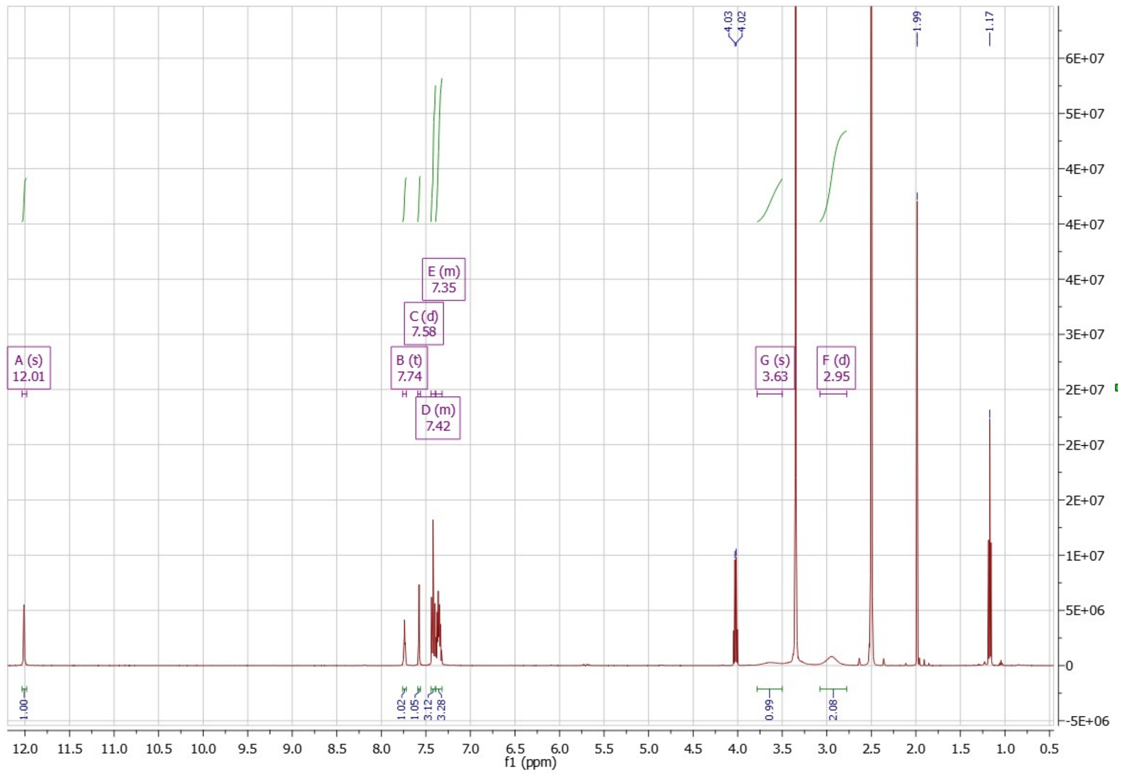

Figure S22. ^{1}H NMR spectrum of **Vb** in DMSO-d_6.

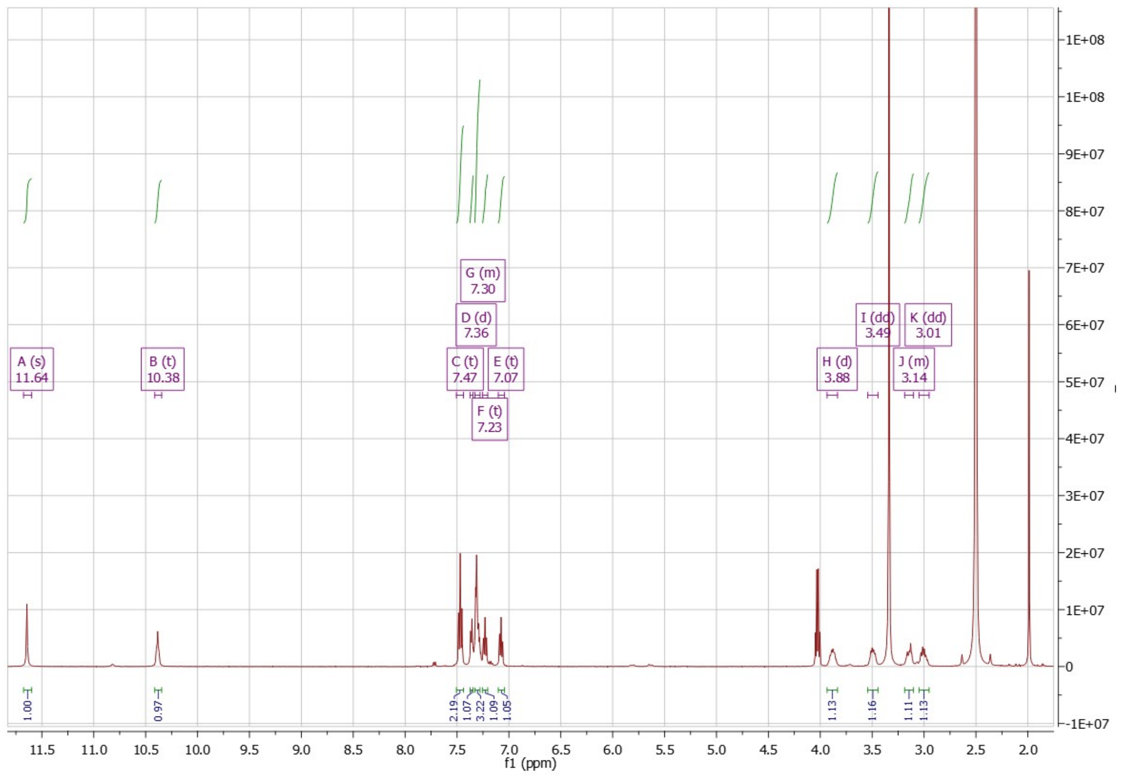

Figure S23. ^{1}H NMR spectrum of **VIa** in DMSO-d_6.

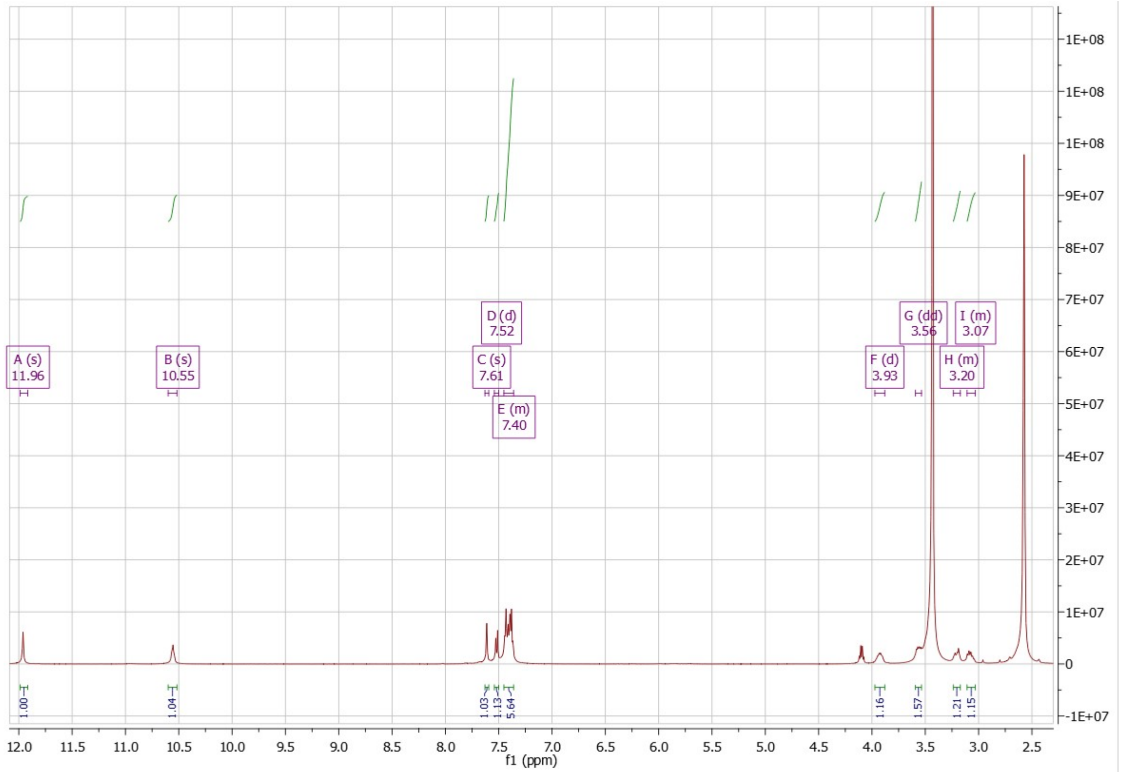

Figure S24. ^{1}H NMR spectrum of **VIb** in DMSO-d_6.

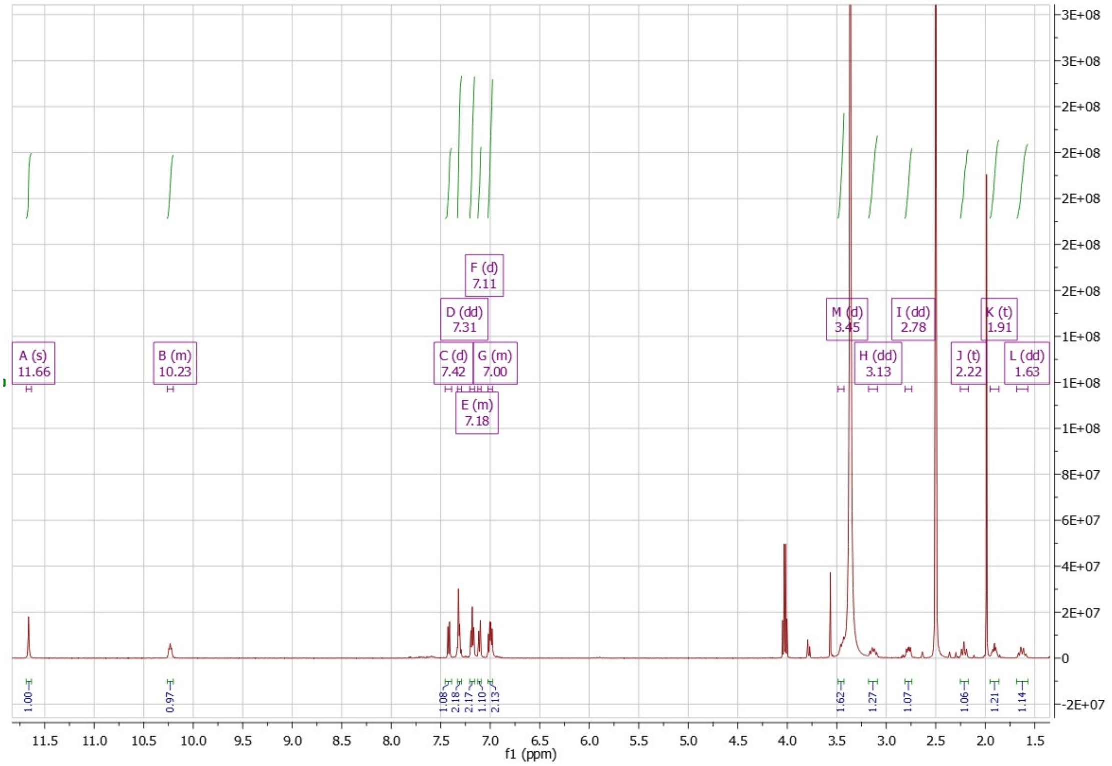

Figure S25. ^{1}H NMR spectrum of **VIc** in DMSO-d_6.

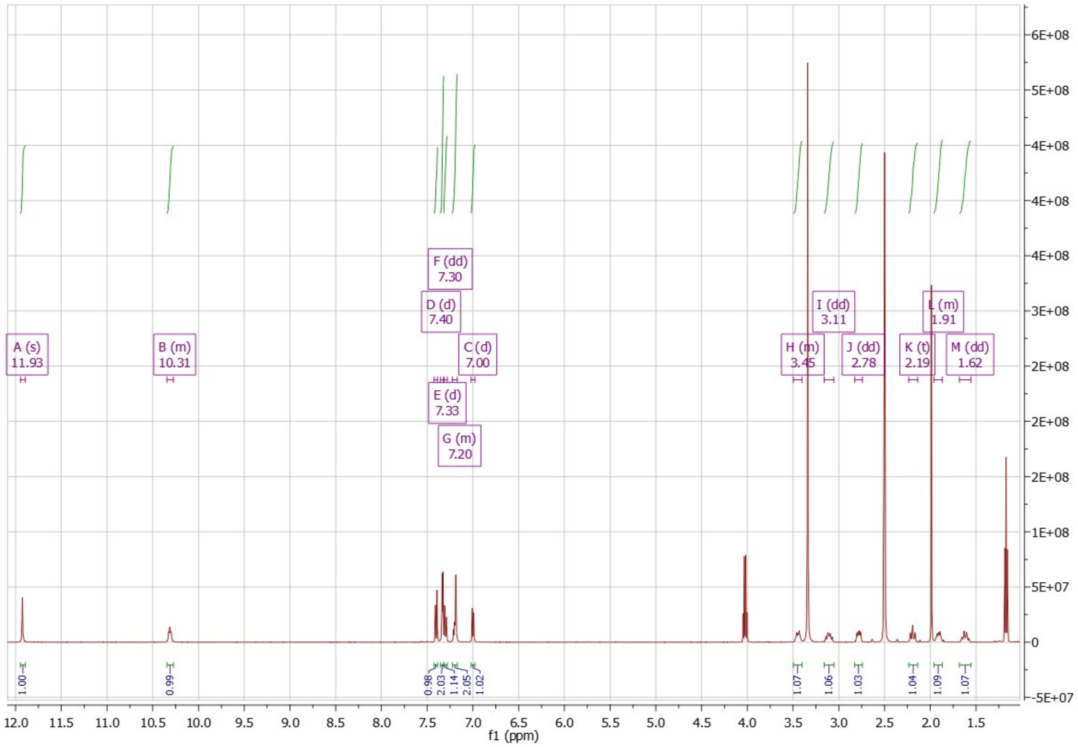

Figure S26. ^{1}H NMR spectrum of **VId** in DMSO-d_6.

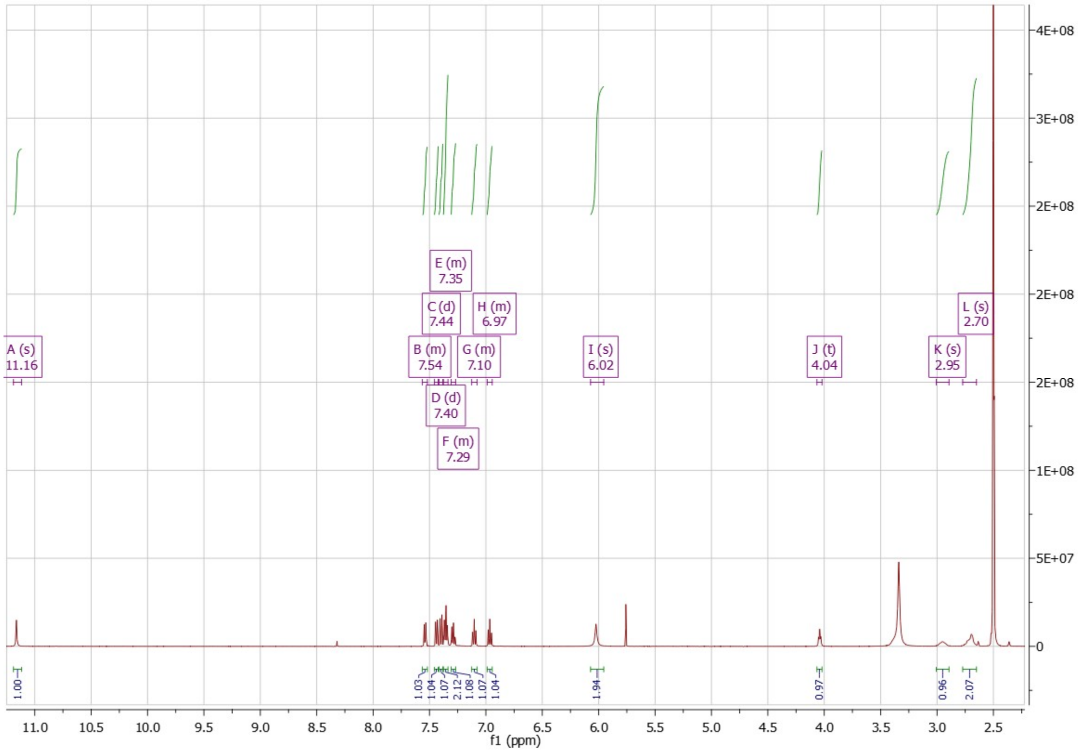

Figure S27. ^{1}H NMR spectrum of **VIIa** in DMSO-d_6.

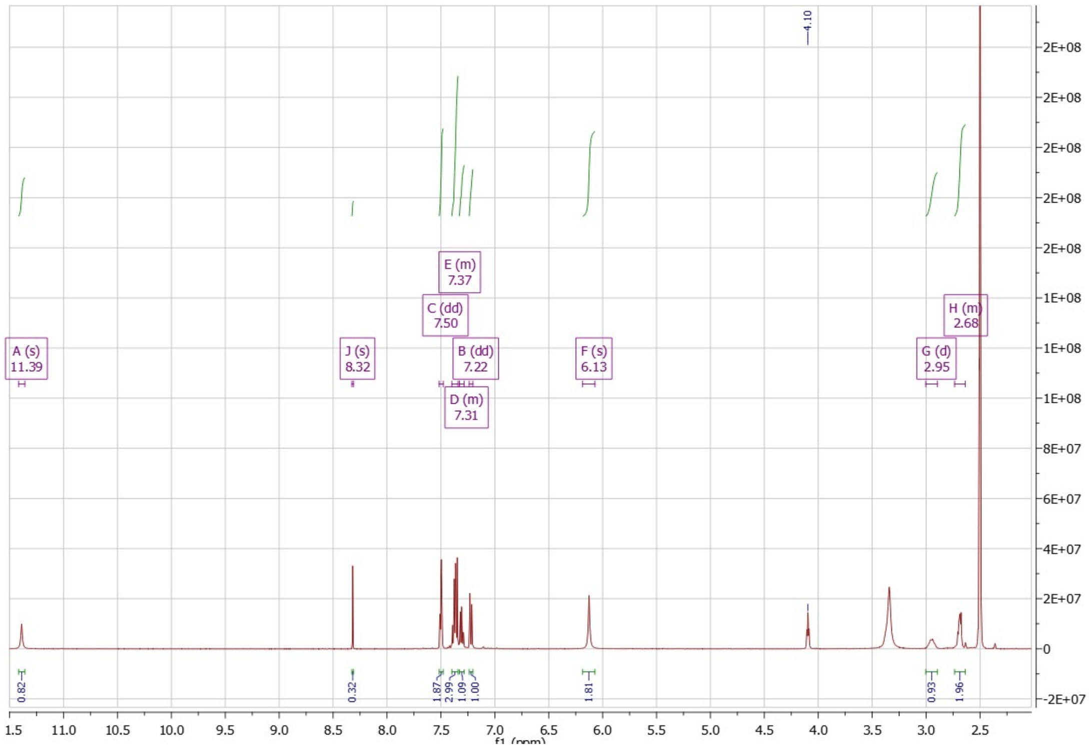

Figure S28. ¹H NMR spectrum of **VIIb** in DMSO-d_6.

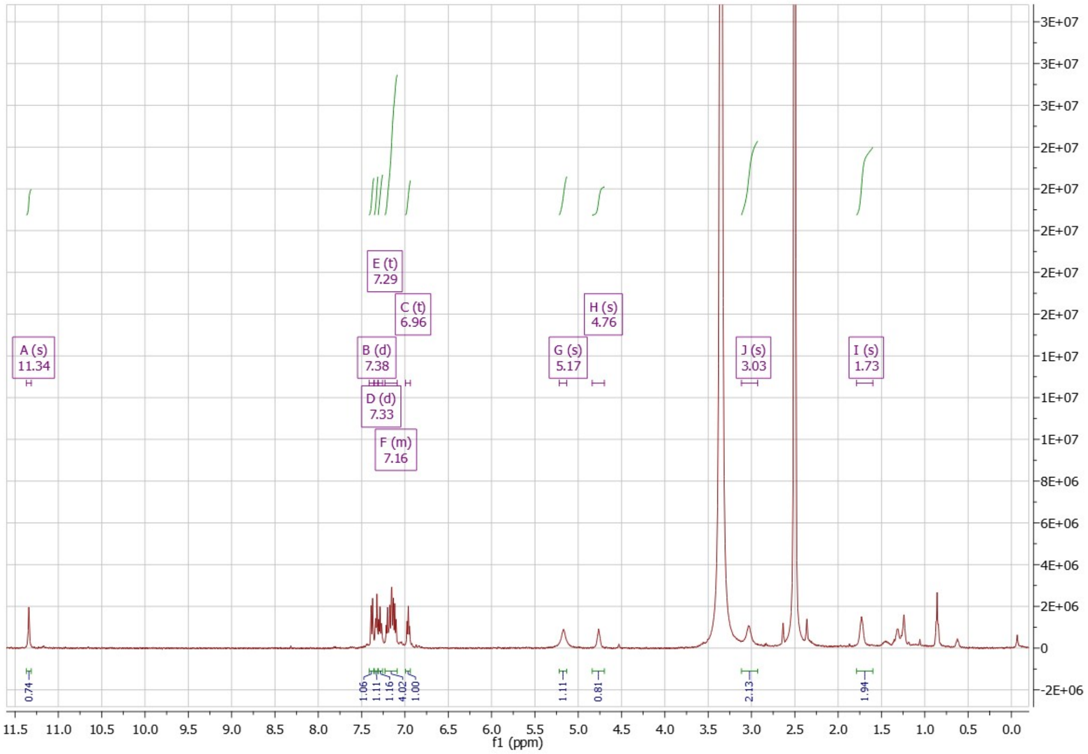

Figure S29. ¹H NMR spectrum of **VIIc** in DMSO-d_6.

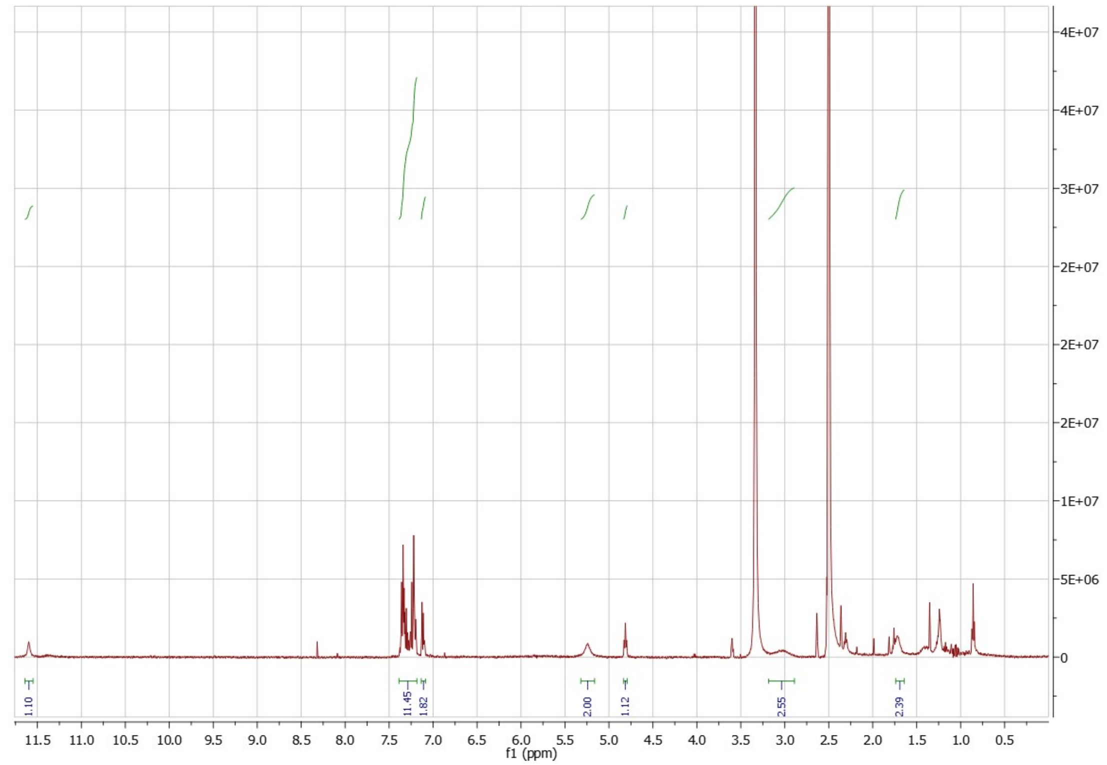

Figure S30. ^{1}H NMR spectrum of **VIId** in DMSO-d_6.

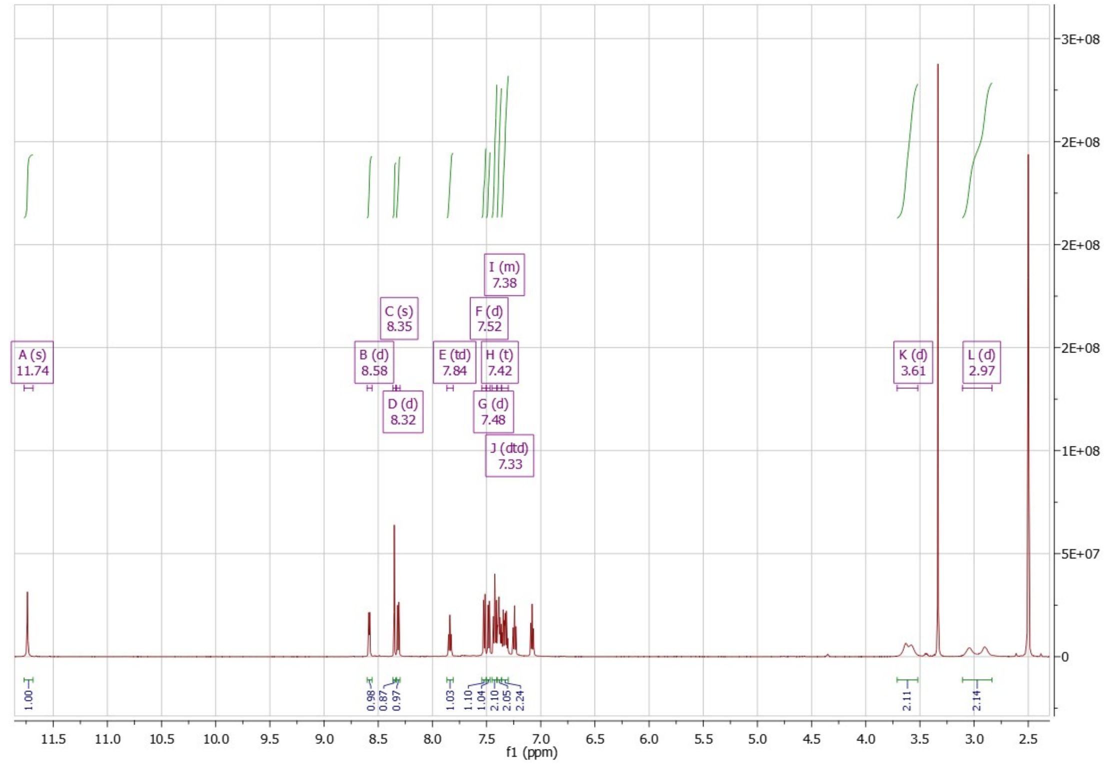

Figure S31. ^{1}H NMR spectrum of **HL**^{1} in DMSO-d_6.

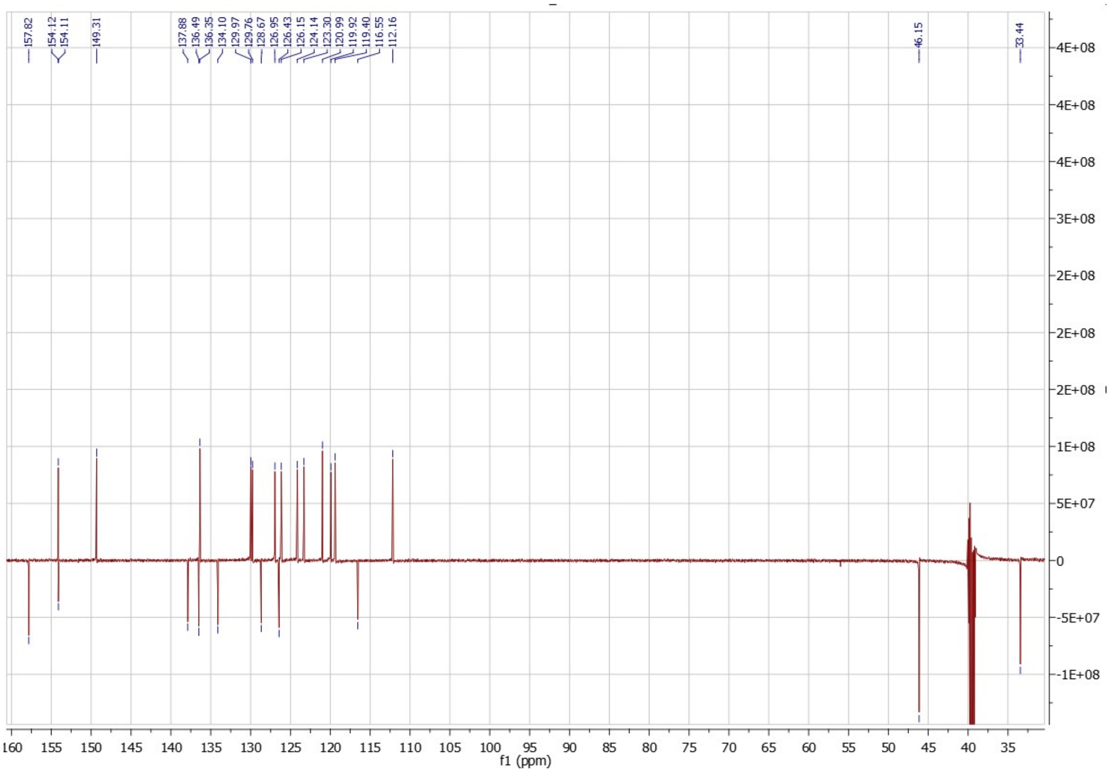

Figure S32. ^{13}C NMR spectrum of **HL^1** in DMSO-d_6.

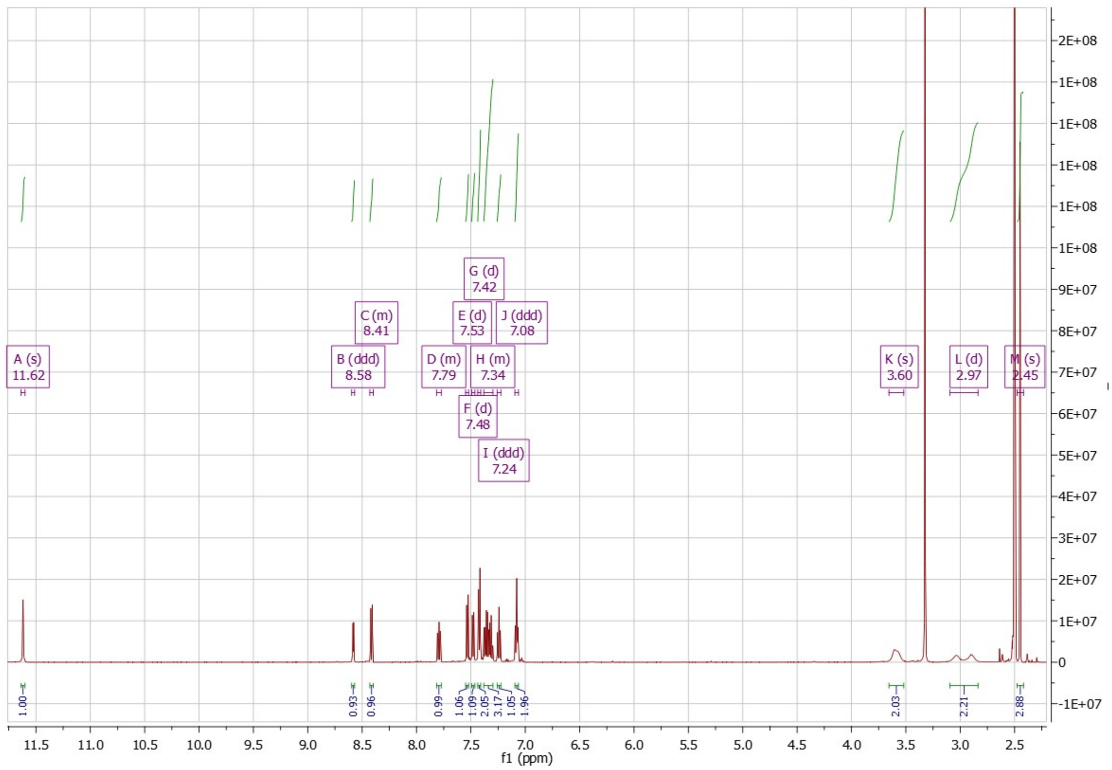

Figure S33. ^{1}H NMR spectrum of **HL^2** in DMSO-d_6.

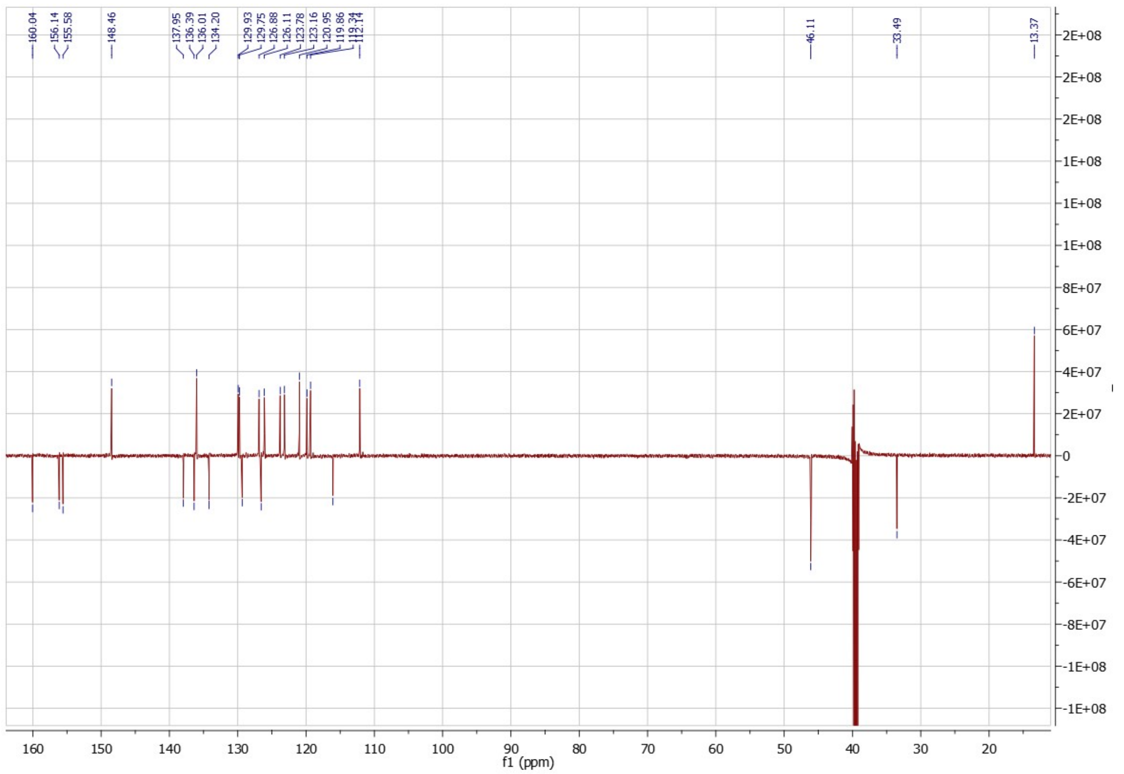

Figure S34. ^{13}C NMR spectrum of **HL^2** in DMSO-d_6.

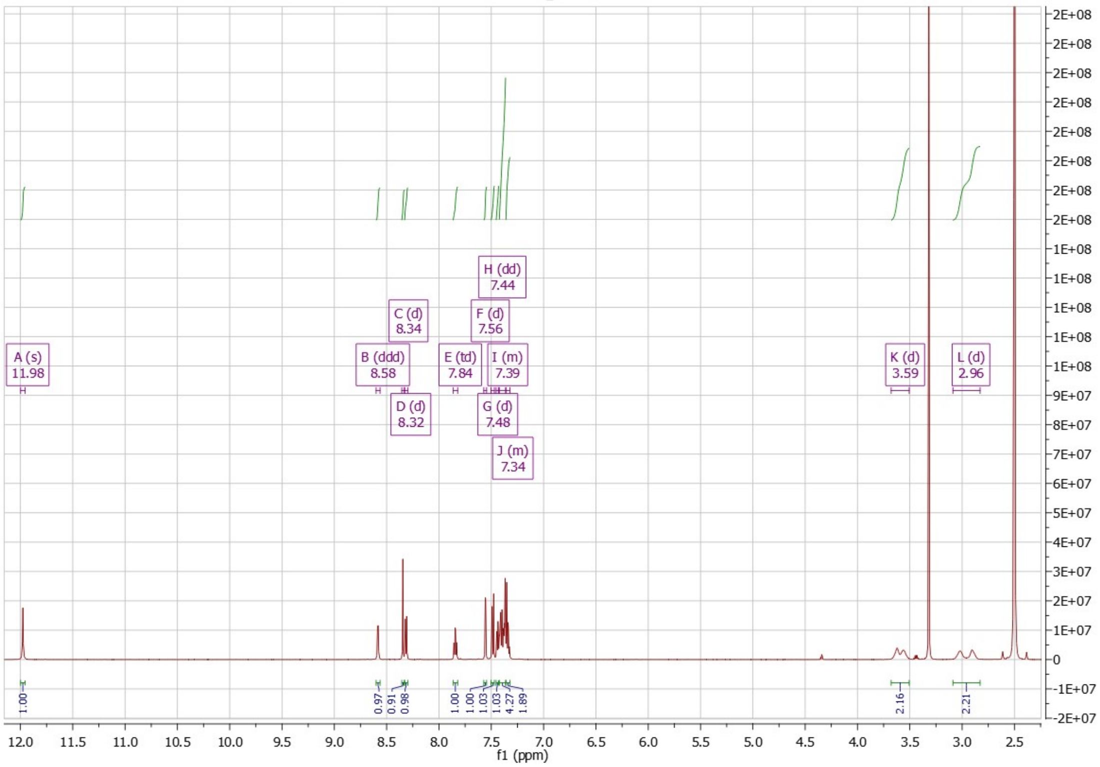

Figure S35. ^{1}H NMR spectrum of **HL^3** in DMSO-d_6.

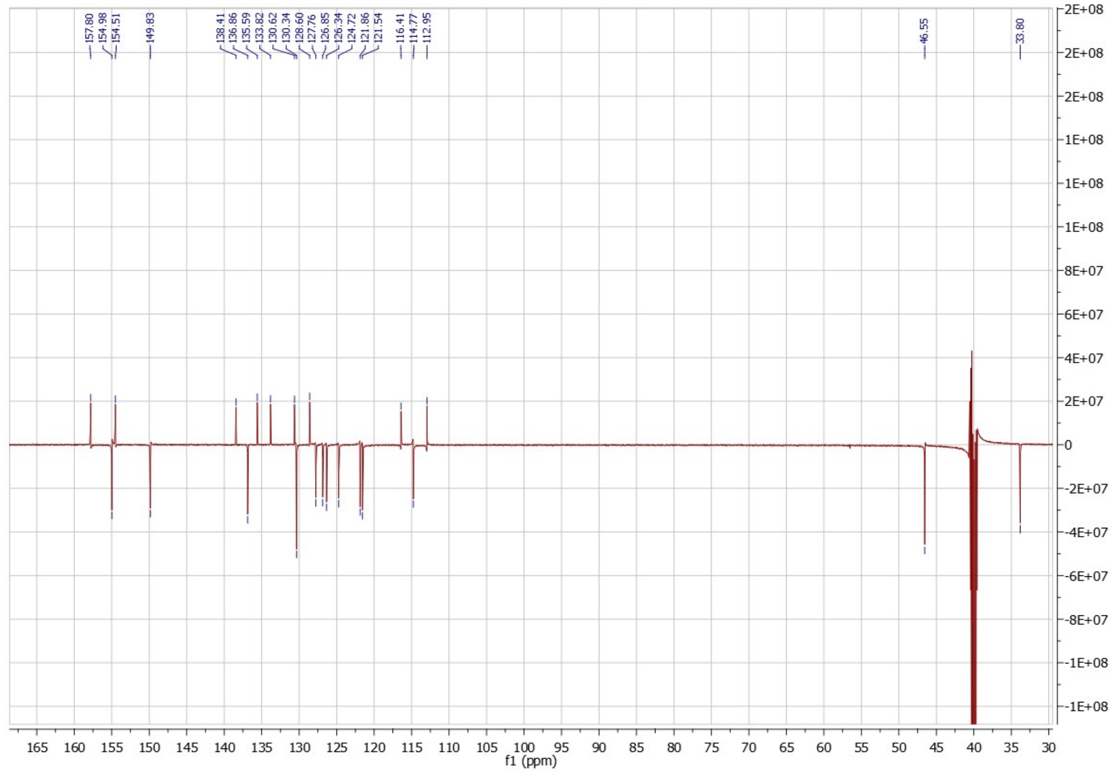

Figure S36. ^{13}C NMR spectrum of $\mathbf{HL^3}$ in DMSO-d_6.

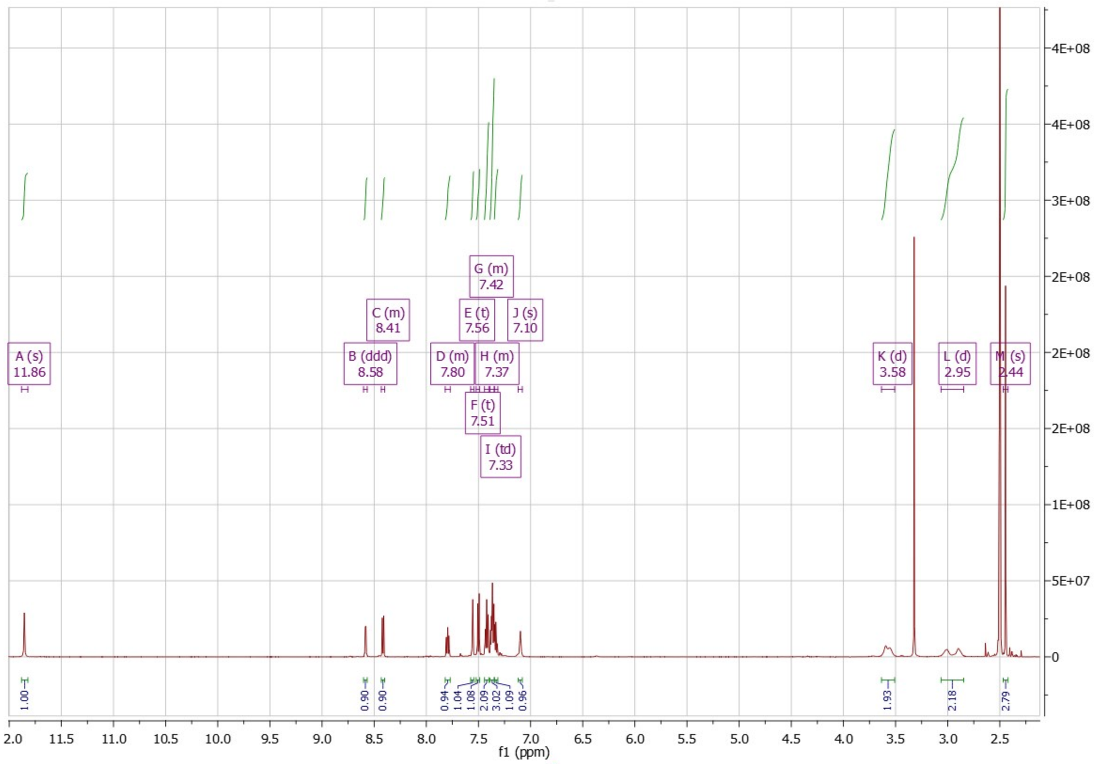

Figure S37. ^{1}H NMR spectrum of $\mathbf{HL^4}$ in DMSO-d_6.

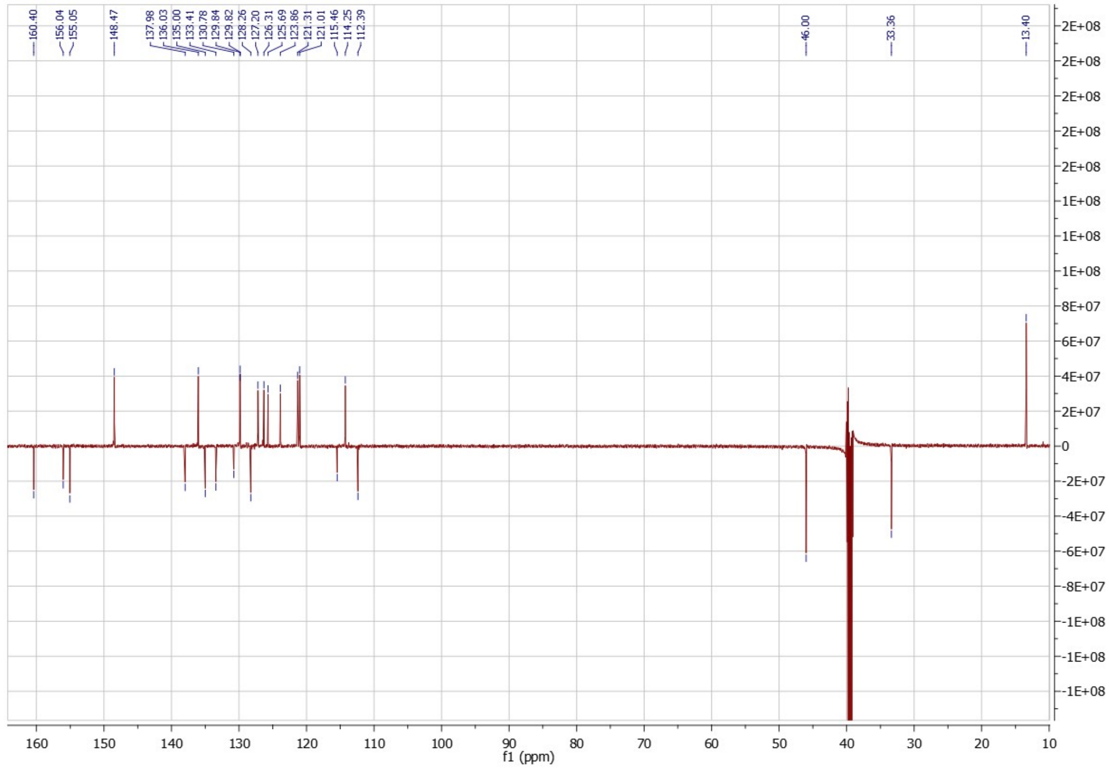

Figure S38. ^{13}C NMR spectrum of **HL⁴** in DMSO-d_6.

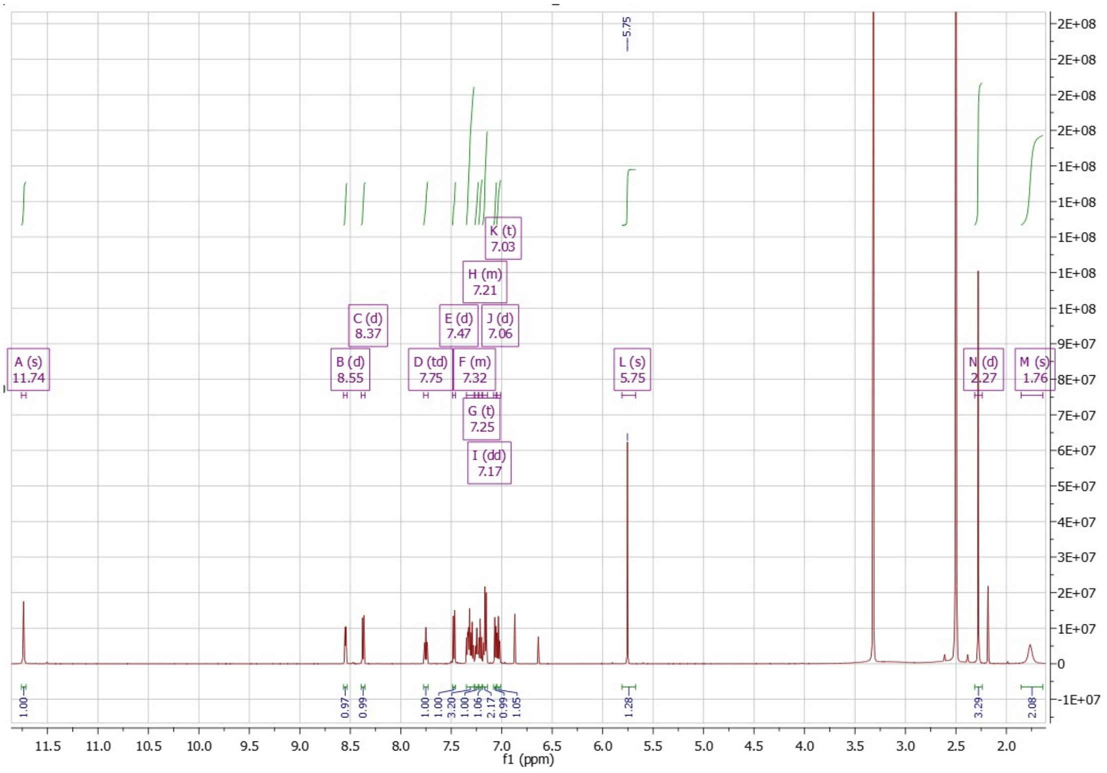

Figure S39. ^{1}H NMR spectrum of **HL⁵** in DMSO-d_6.

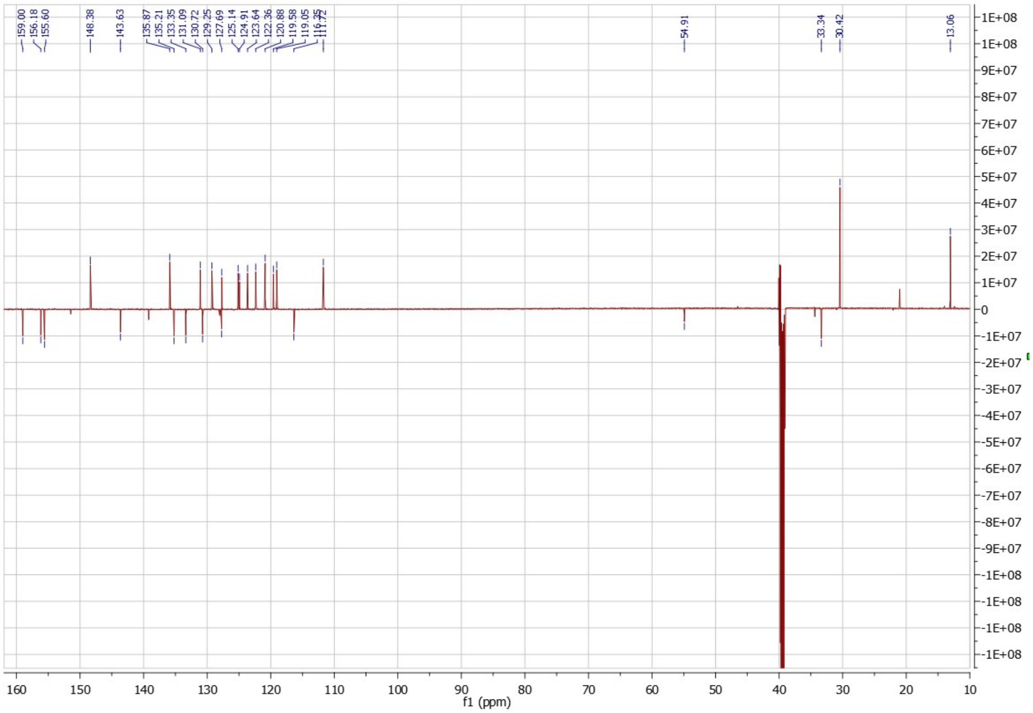

Figure S40. ^{13}C NMR spectrum of **HL⁵** in DMSO-d_6.

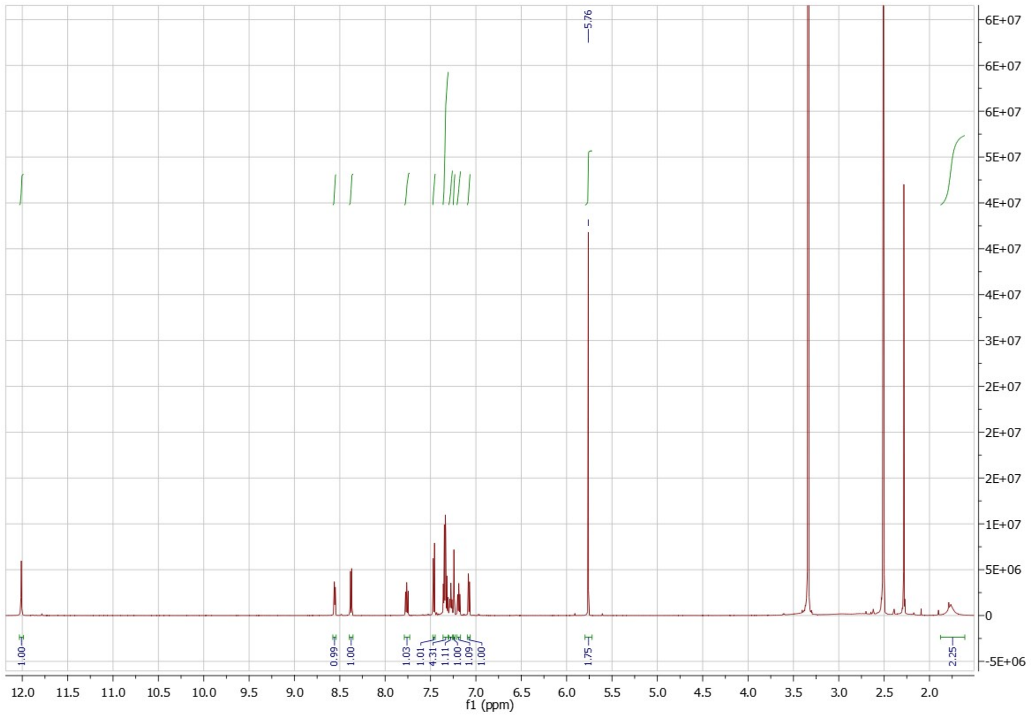

Figure S41. ^{1}H NMR spectrum of **HL⁶** in DMSO-d_6.

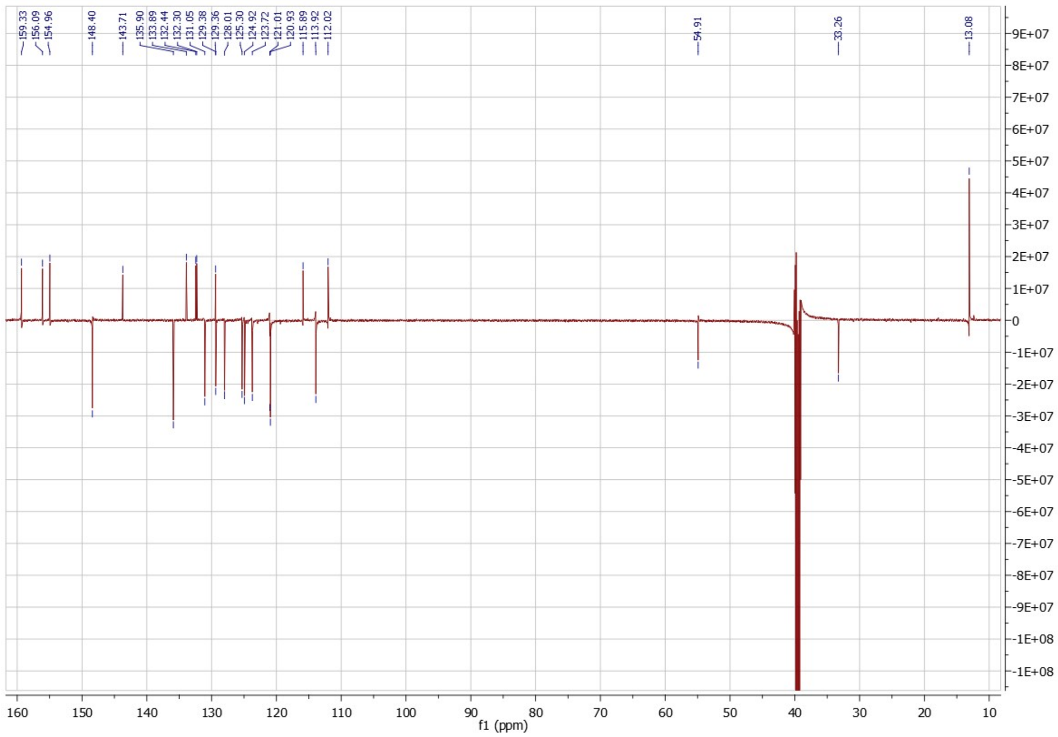

Figure S42. ^{13}C NMR spectrum of $\mathbf{HL^6}$ in DMSO-d_6.

- ESI mass spectra

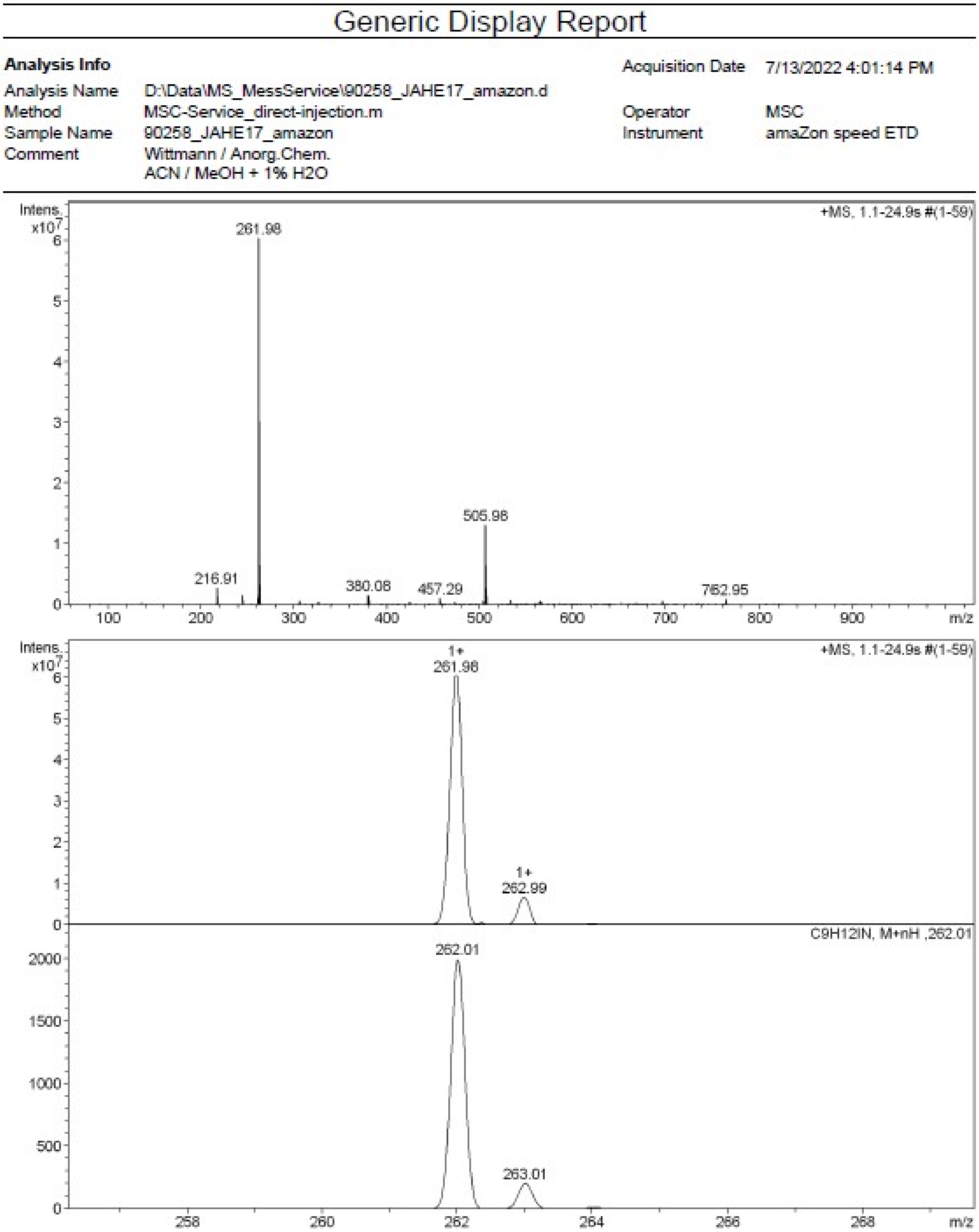

Figure S43. ESI mass spectrum of **X4** in positive ion mode.

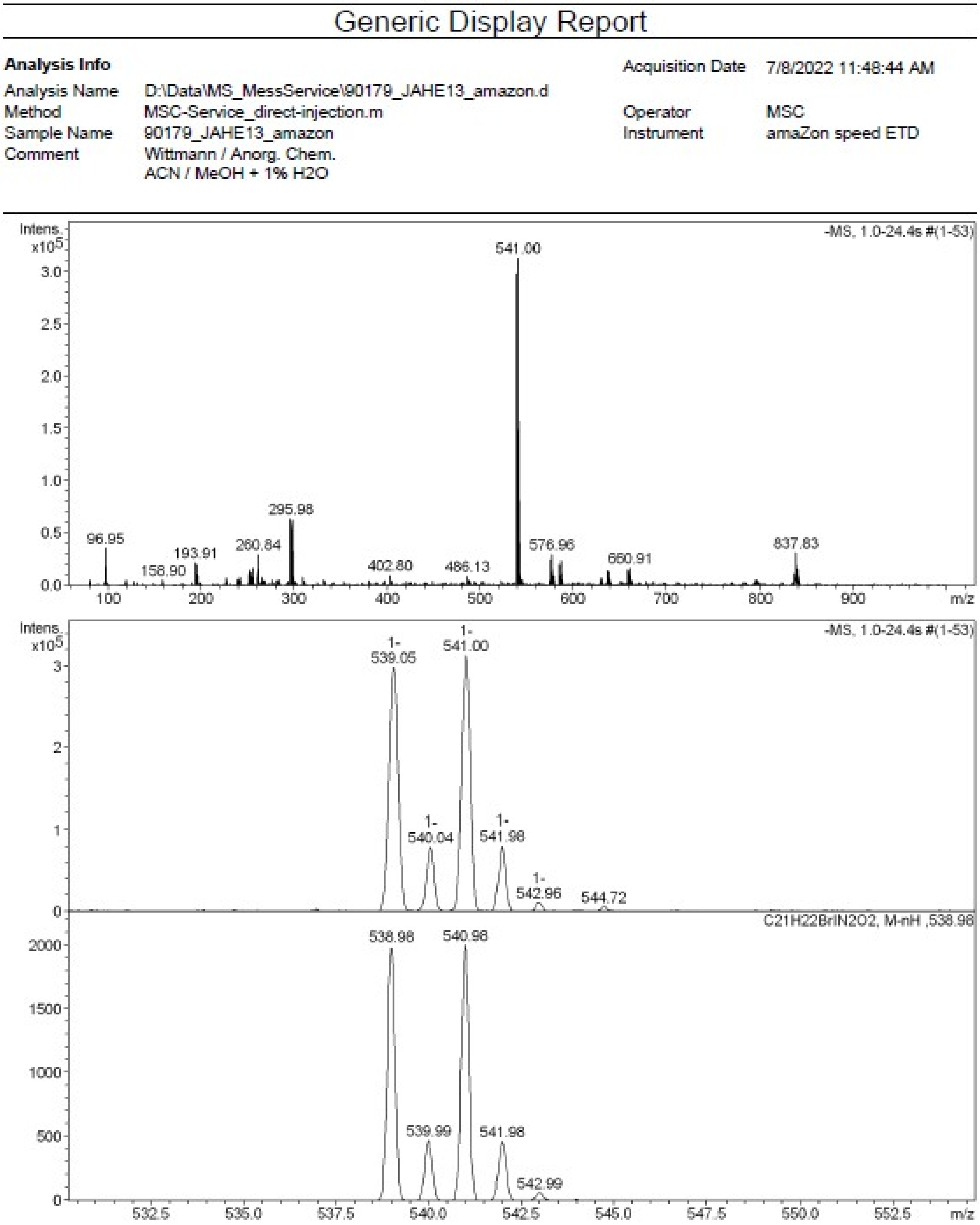

Figure S44. ESI mass spectrum of **IId** in negative ion mode.

Generic Display Report

**Analysis Info**		Acquisition Date	9/1/2020 8:54:37 AM
Analysis Name	D:\Data\MS_MessService\72802_CHWI432_amazon.d		
Method	MSC-Service_direct-injection.m	Operator	MSC
Sample Name	72802_CHWI432_amazon	Instrument	amaZon speed ETD
Comment	Wittmann / AOC<br>MeOH / ACN + 1% H2O		

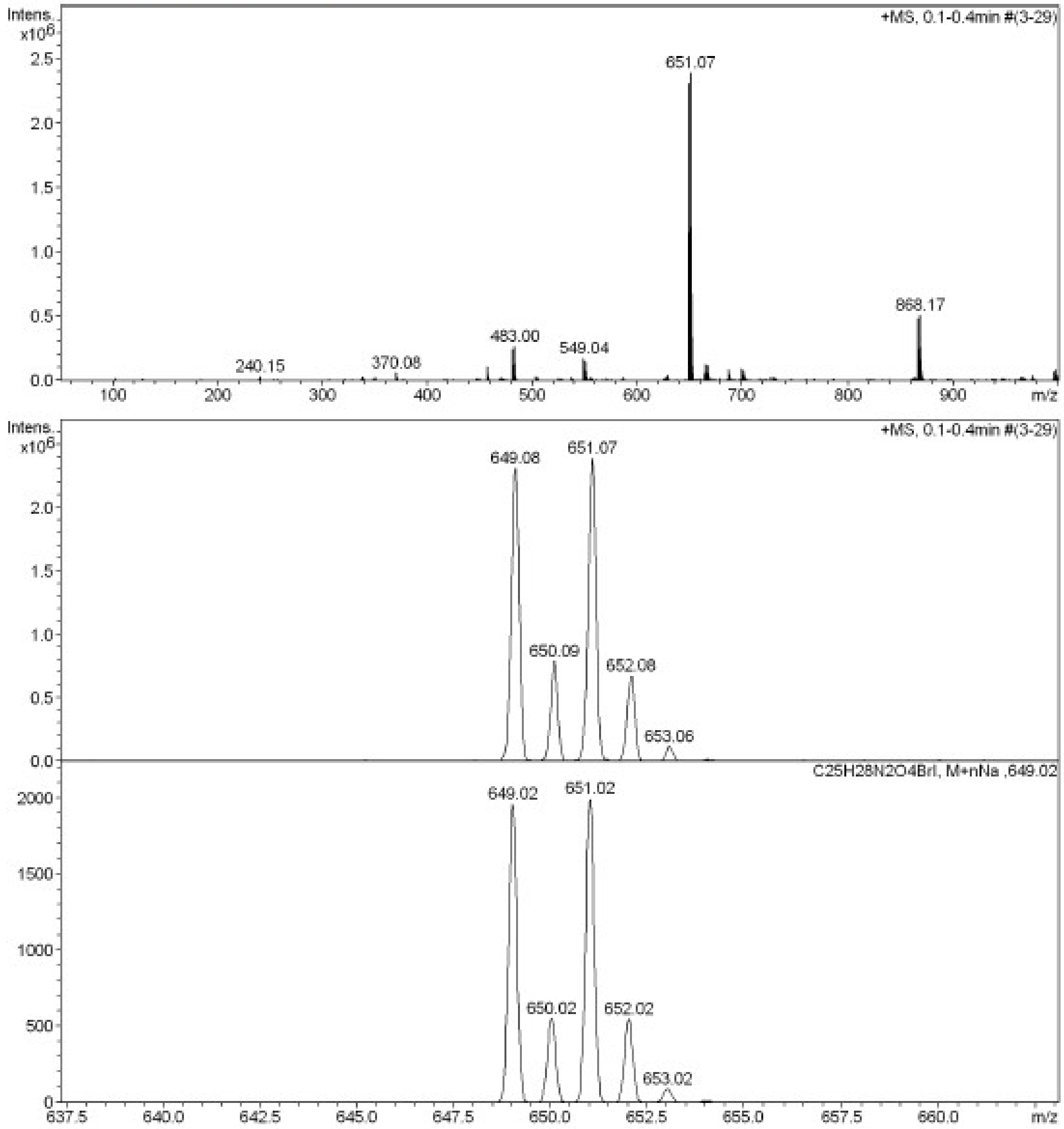

Figure S45. ESI mass spectrum of **IIIb** in positive ion mode.

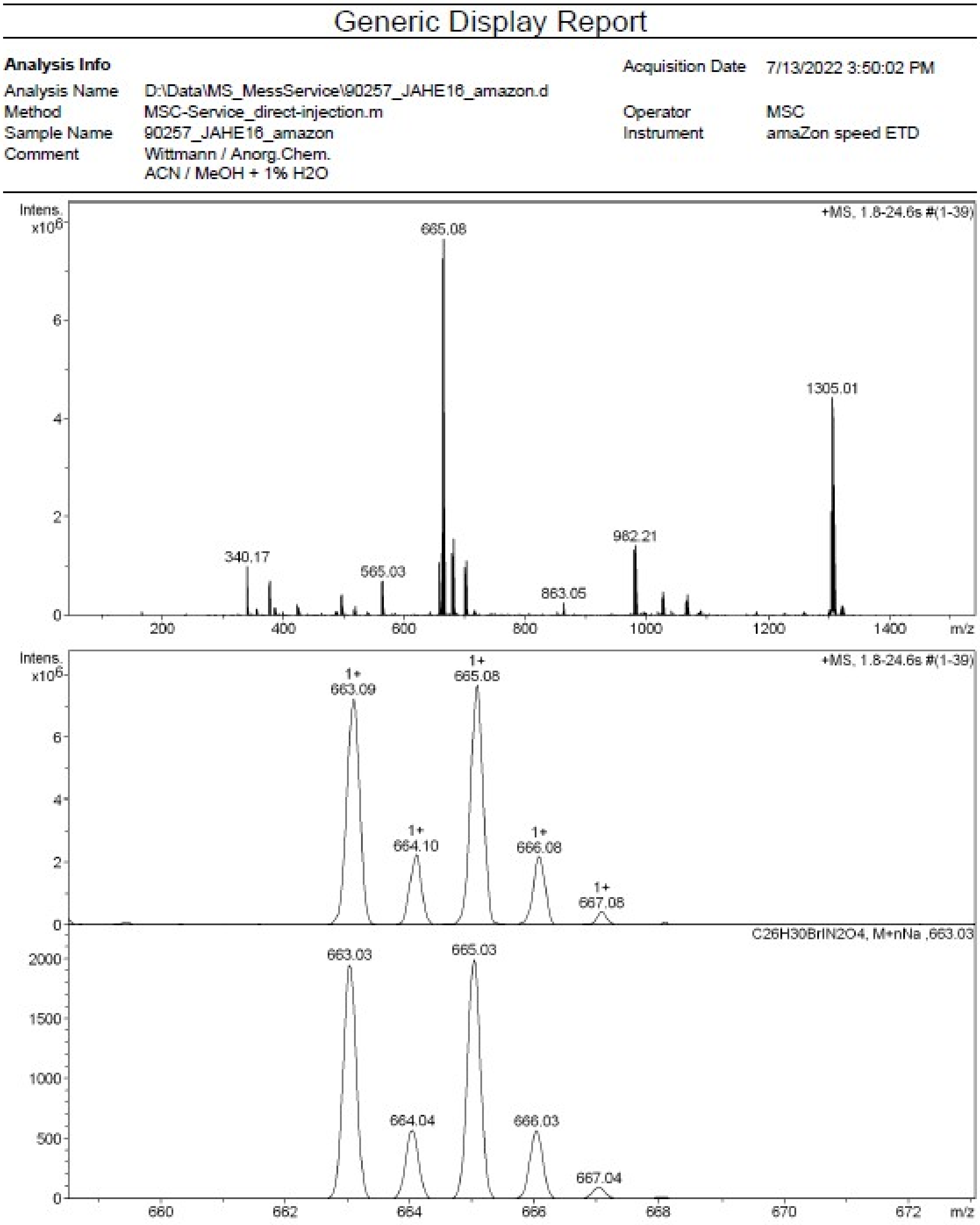

Figure S46. ESI mass spectrum of **IIId** in positive ion mode.

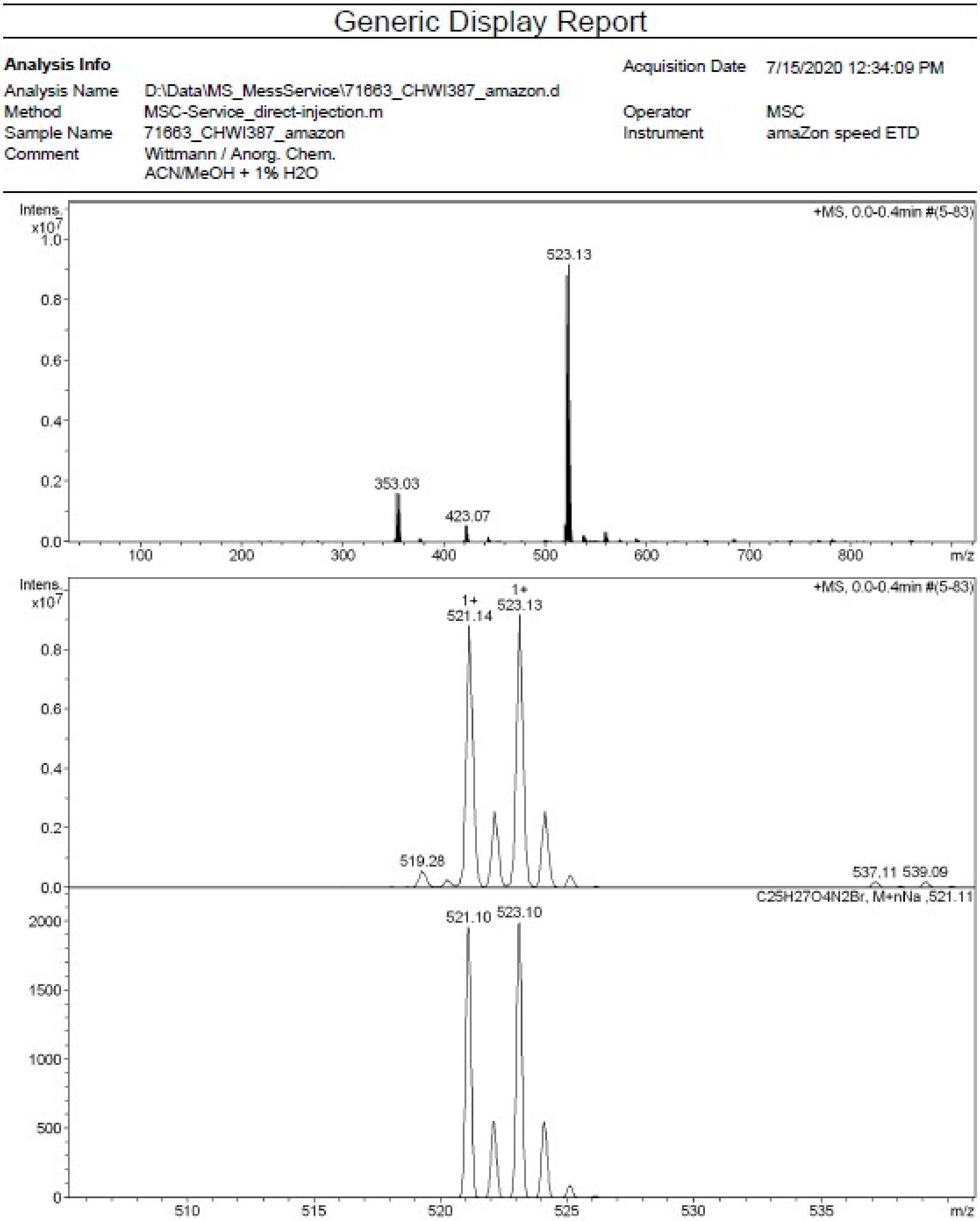

Figure S47. ESI mass spectrum of **IVb** in positive ion mode.

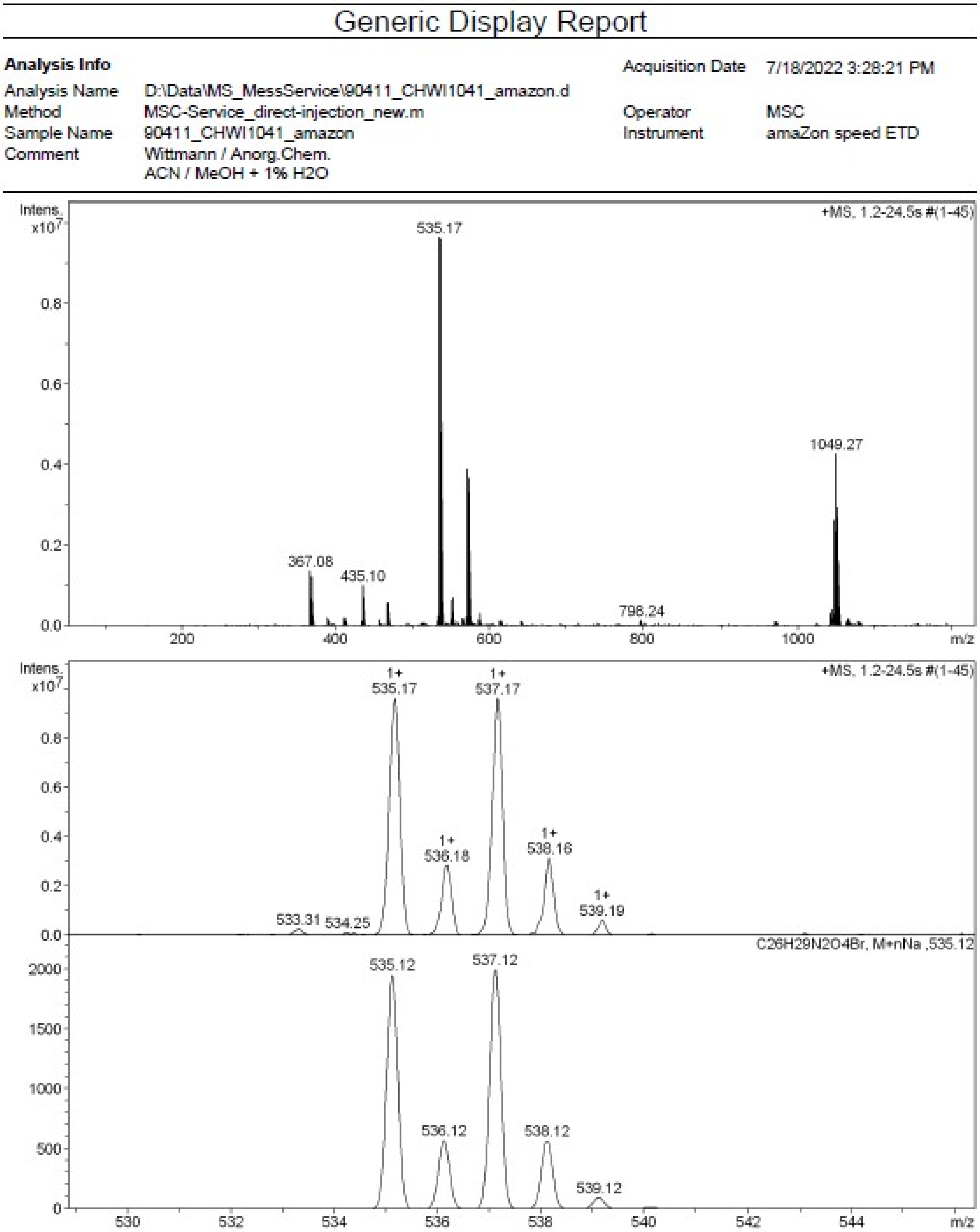

Figure S48. ESI mass spectrum of **IVd** in positive ion mode.

Generic Display Report

Analysis Info		Acquisition Date	11/6/2020 4:31:13 PM
Analysis Name	D:\Data\MS_MessService\74594_CHWI514_amazon.d		
Method	lc-mc_1100_normal_flow_06okt2020_5518.m	Operator	MSC
Sample Name	74594_CHWI514_amazon	Instrument	amaZon speed ETD
Comment	Wittmann / AOC<br>ACN / MeOH + 1% H2O		

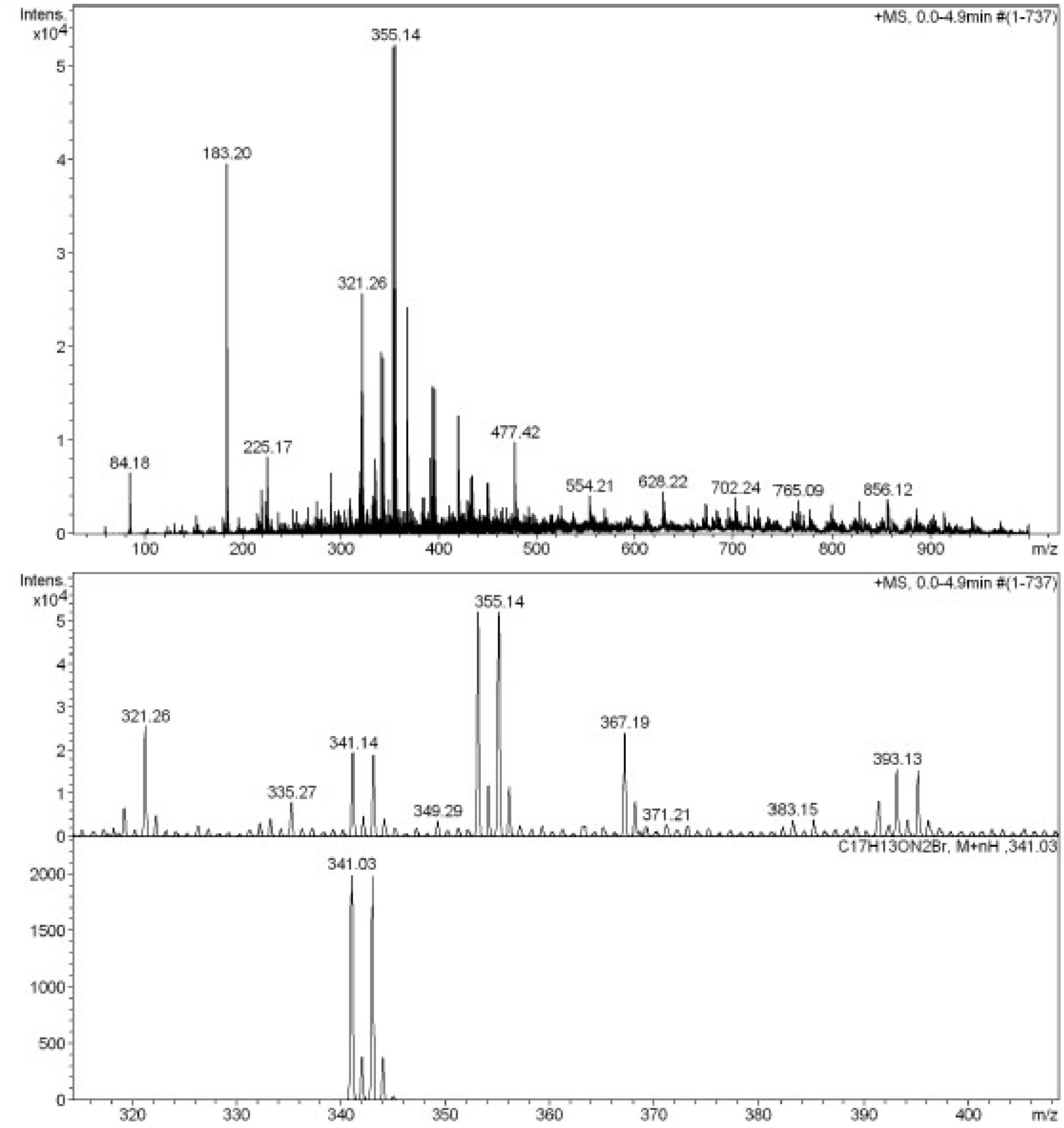

Figure S49. ESI mass spectrum of **Vb** in positive ion mode.

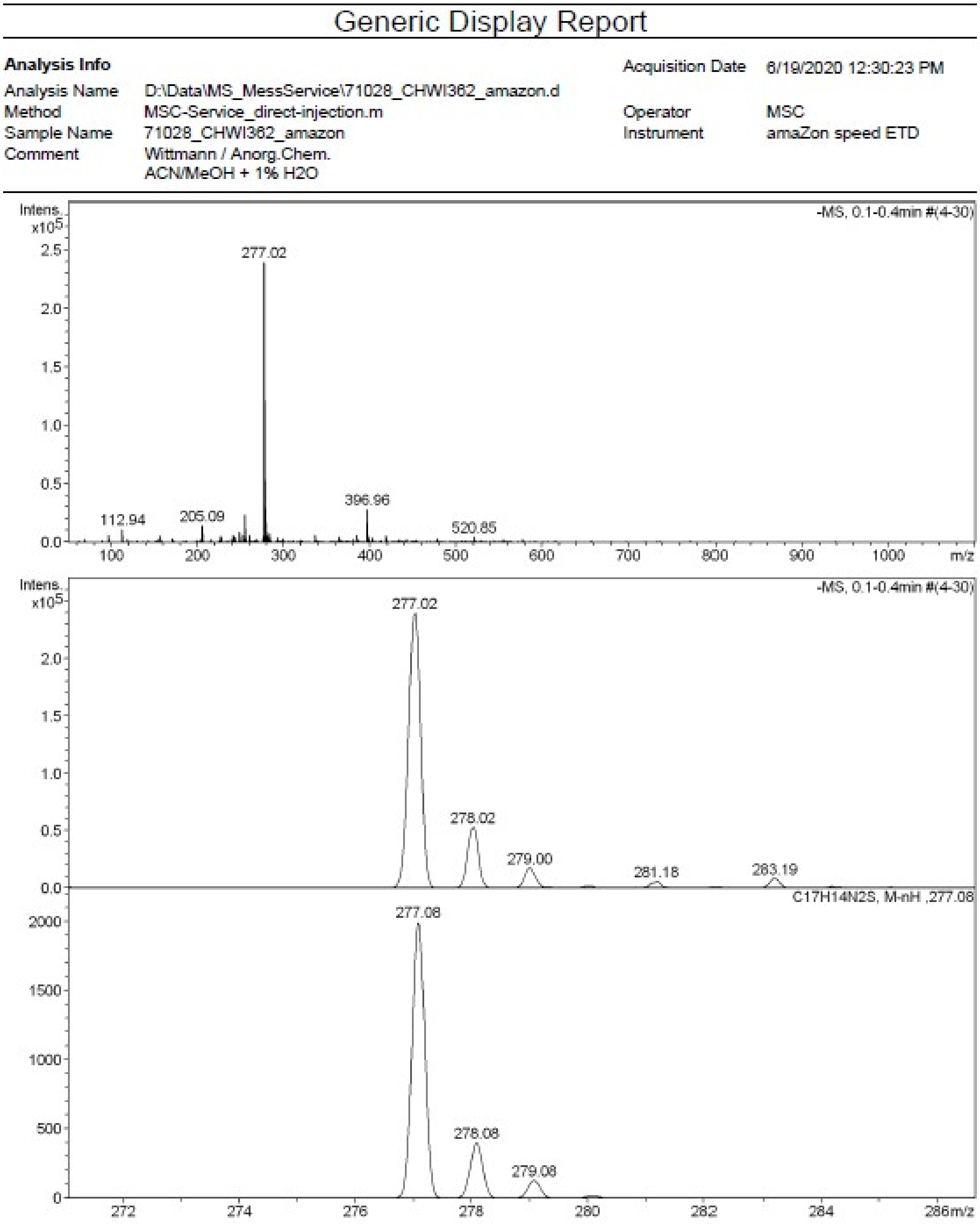

Figure S50. ESI mass spectrum of **VIa** in negative ion mode.

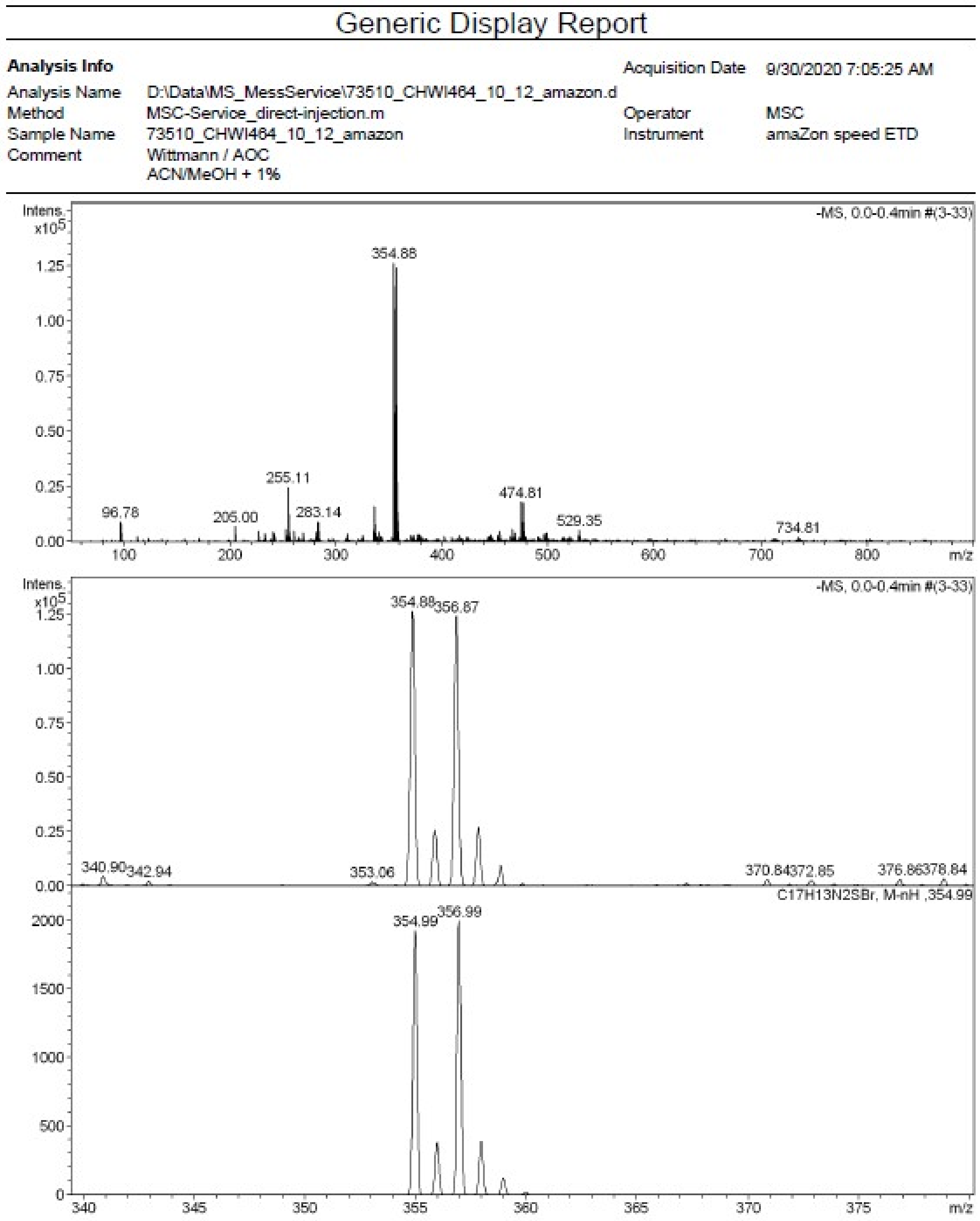

Figure S51. ESI mass spectrum of **VIb** in negative ion mode.

Generic Display Report

Analysis Info

Analysis Name	D:\Data\MS_MessService\92724_RIUR 04.2_amazon.d	Acquisition Date	11/3/2022 10:39:31 AM
Method	MSC-Service_direct-injection.m	Operator	MSC
Sample Name	92724_RIUR 04.2_amazon	Instrument	amaZon speed ETD
Comment	Wittmann / AOC<br>ACN / MeOH +1% H2O		

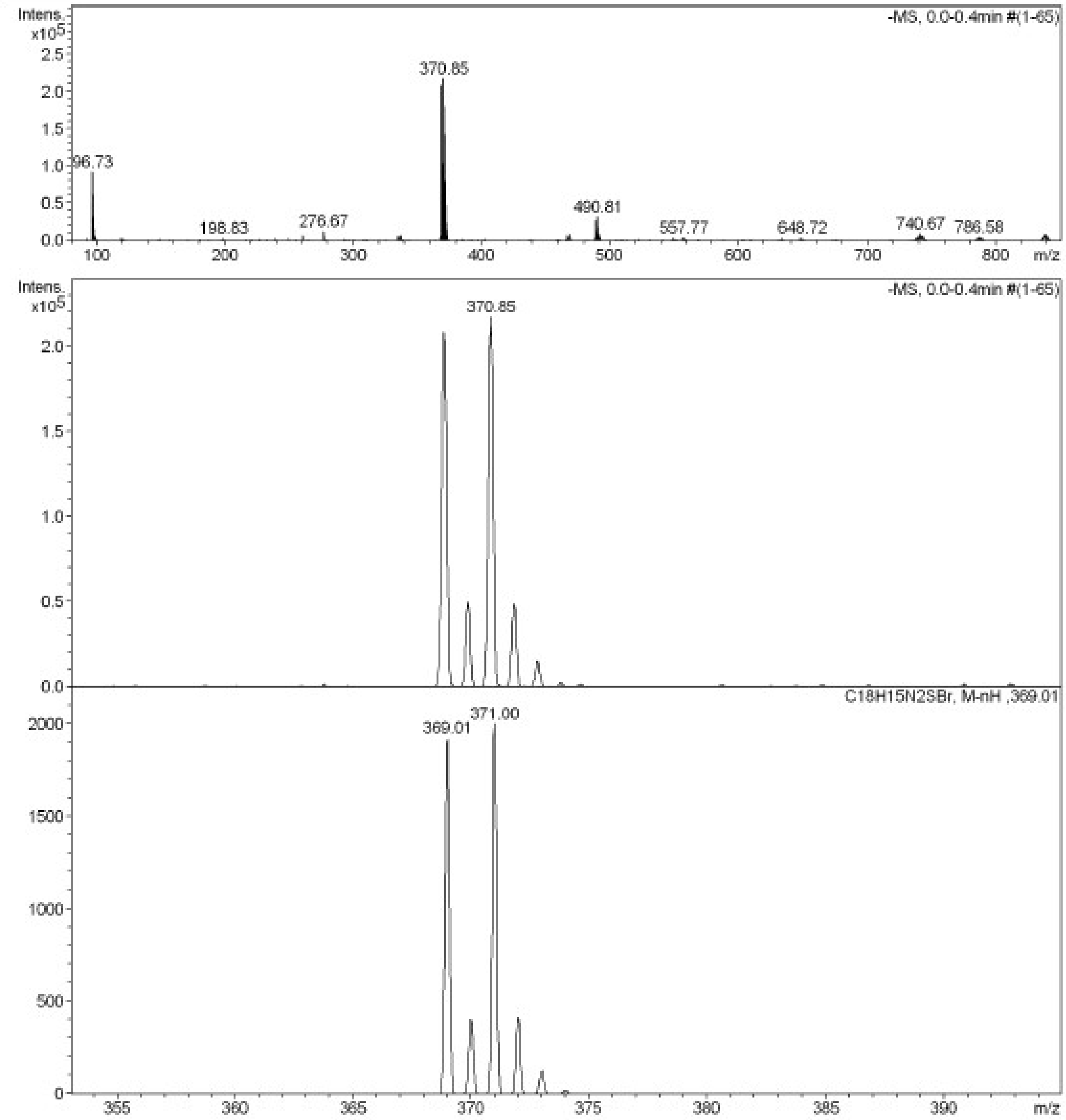

Figure S52. ESI mass spectrum of **VId** in negative ion mode.

Generic Display Report

Analysis Info

Analysis Name	D:\Data\MS_MessService\78114_CHWI655_amazon.d	Acquisition Date	3/11/2021 12:53:26 PM
Method	MSC-Service_direct-injection.m	Operator	MSC
Sample Name	78114_CHWI655_amazon	Instrument	amaZon speed ETD
Comment	Wittmann / Anorg.Chem.<br>ACN / MeOH + 1% H2O		

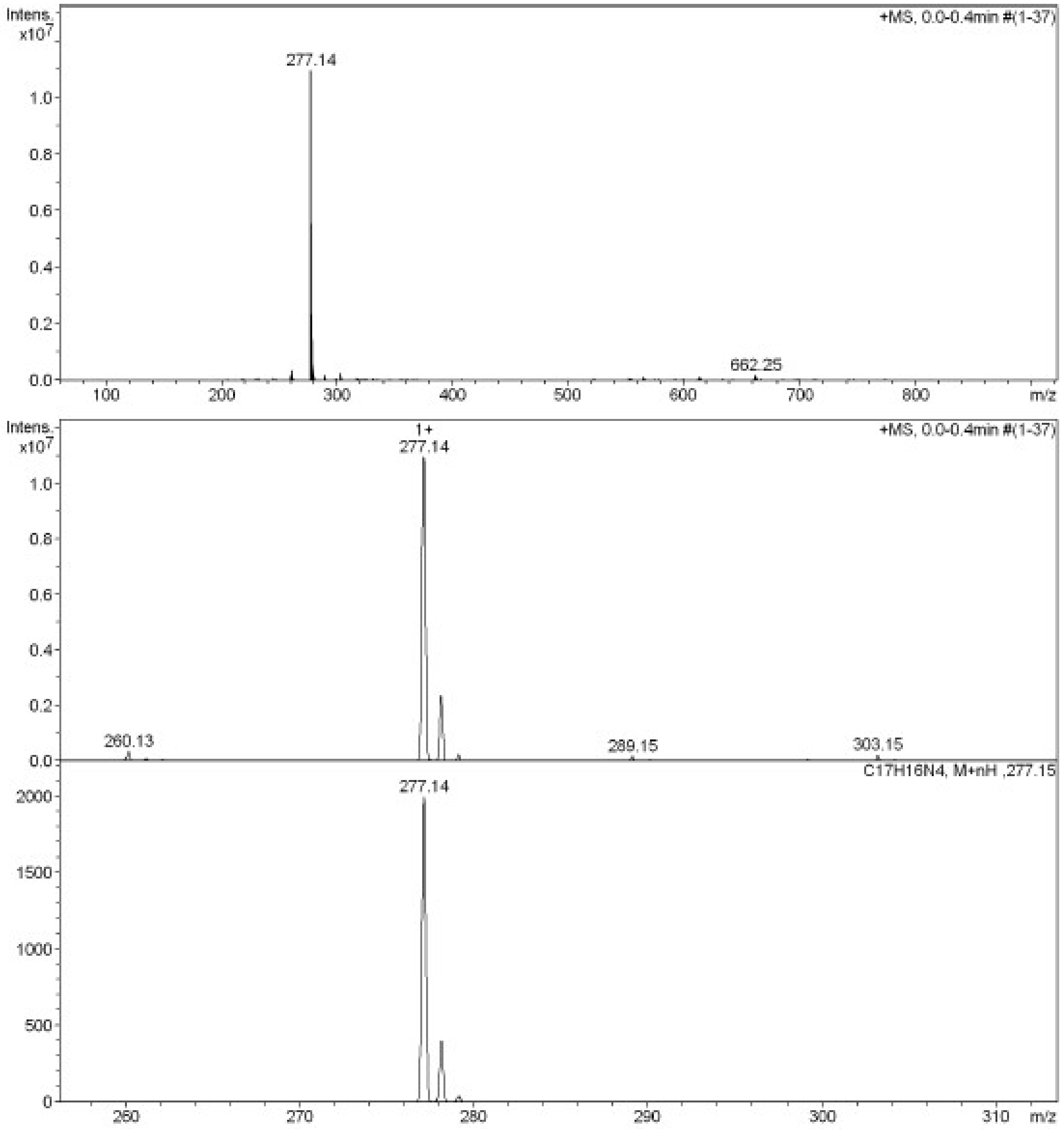

Figure S53. ESI mass spectrum of **VIIa** in positive ion mode.

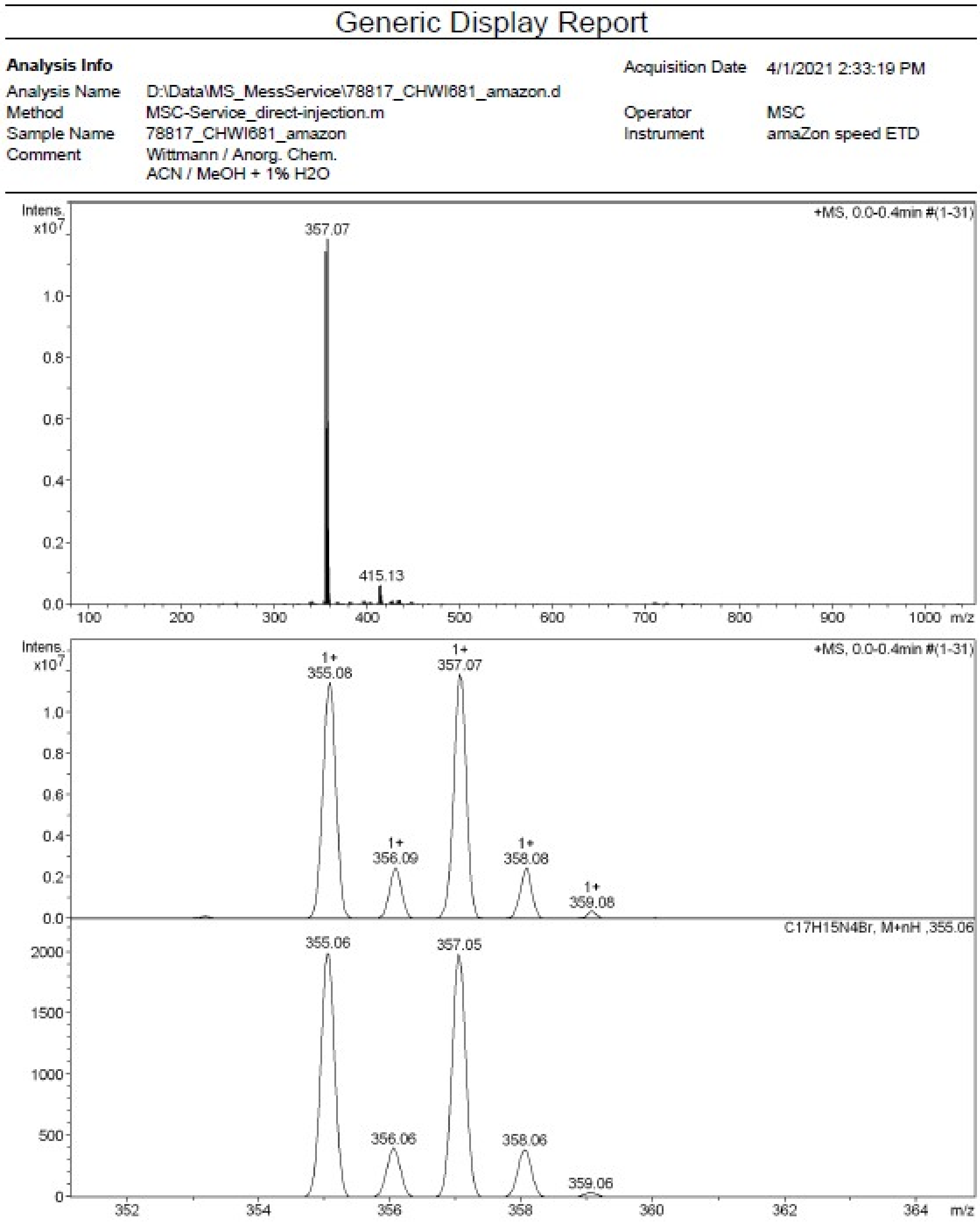

Figure S54. ESI mass spectrum of **VIIb** in positive ion mode.

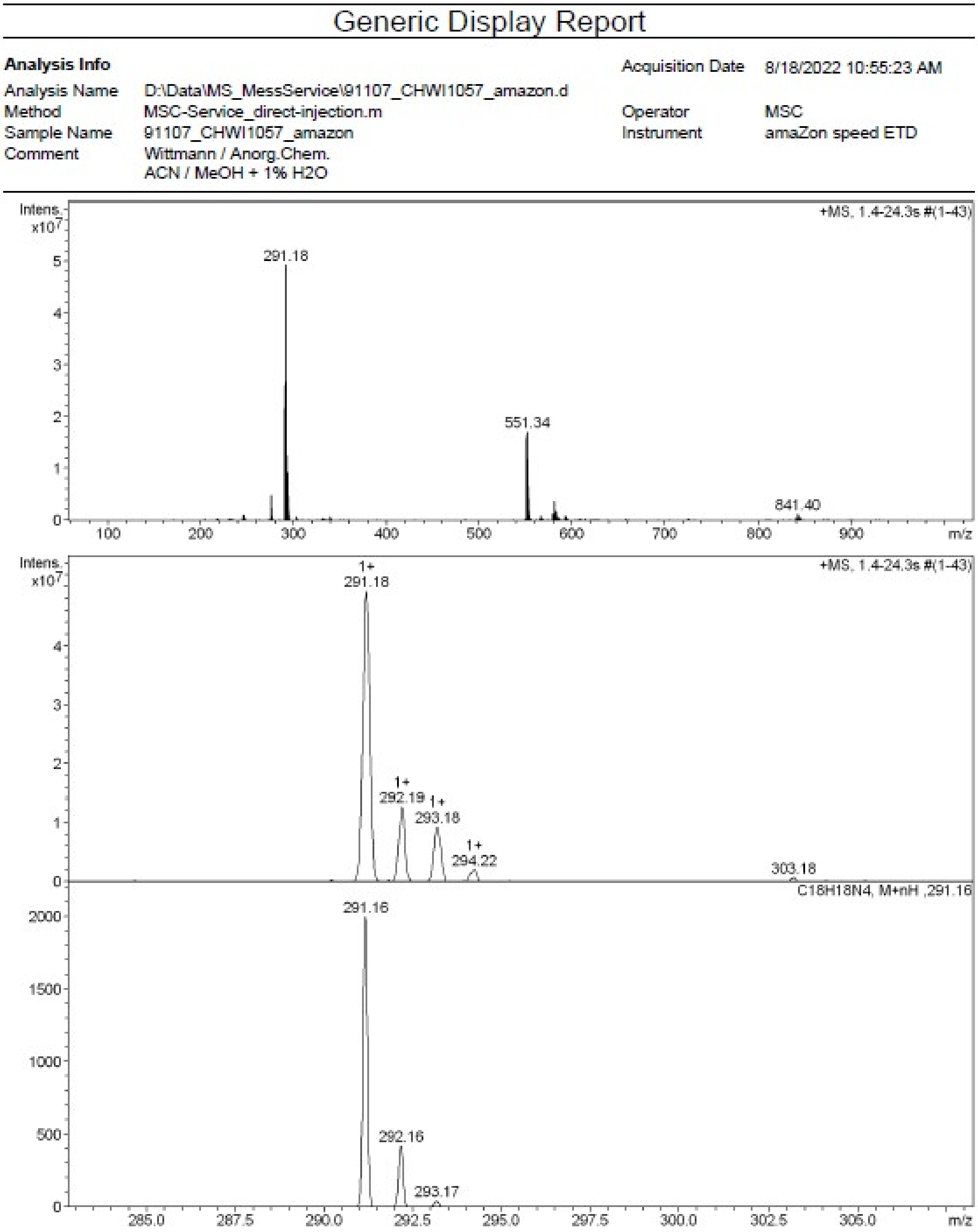

Figure S55. ESI mass spectrum of **VIIc** in positive ion mode.

Generic Display Report

**Analysis Info**		Acquisition Date	11/4/2022 10:15:10 AM
Analysis Name	D:\Data\MS_MessService\92766_RIUR 06_amazon.d		
Method	MSC-Service_direct-injection.m	Operator	MSC
Sample Name	92766_RIUR 06_amazon	Instrument	amaZon speed ETD
Comment	Wittmann / AOC		
	ACN / MeOH +1% H2O		

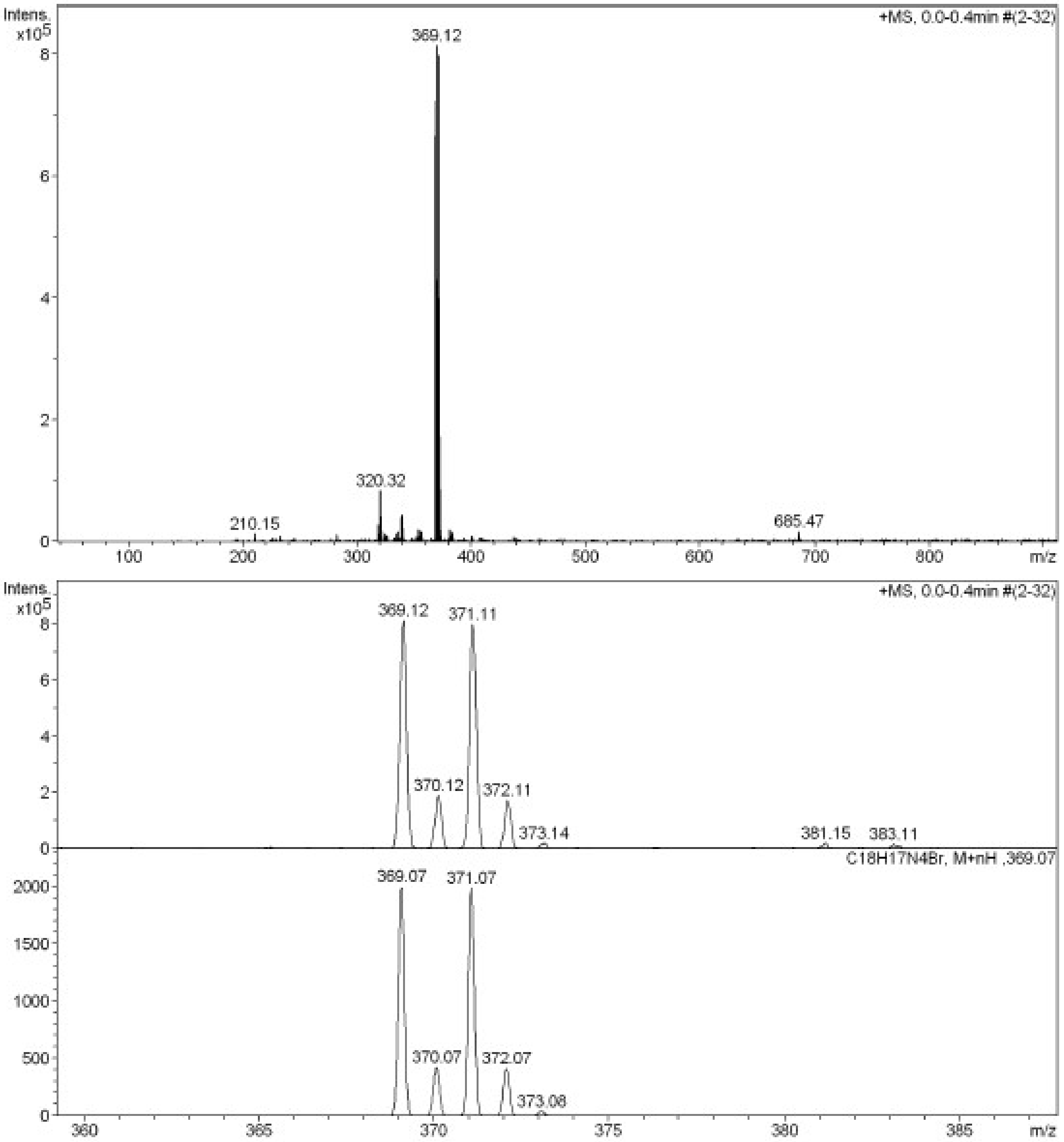

Figure S56. ESI mass spectrum of **VIId** in positive ion mode.

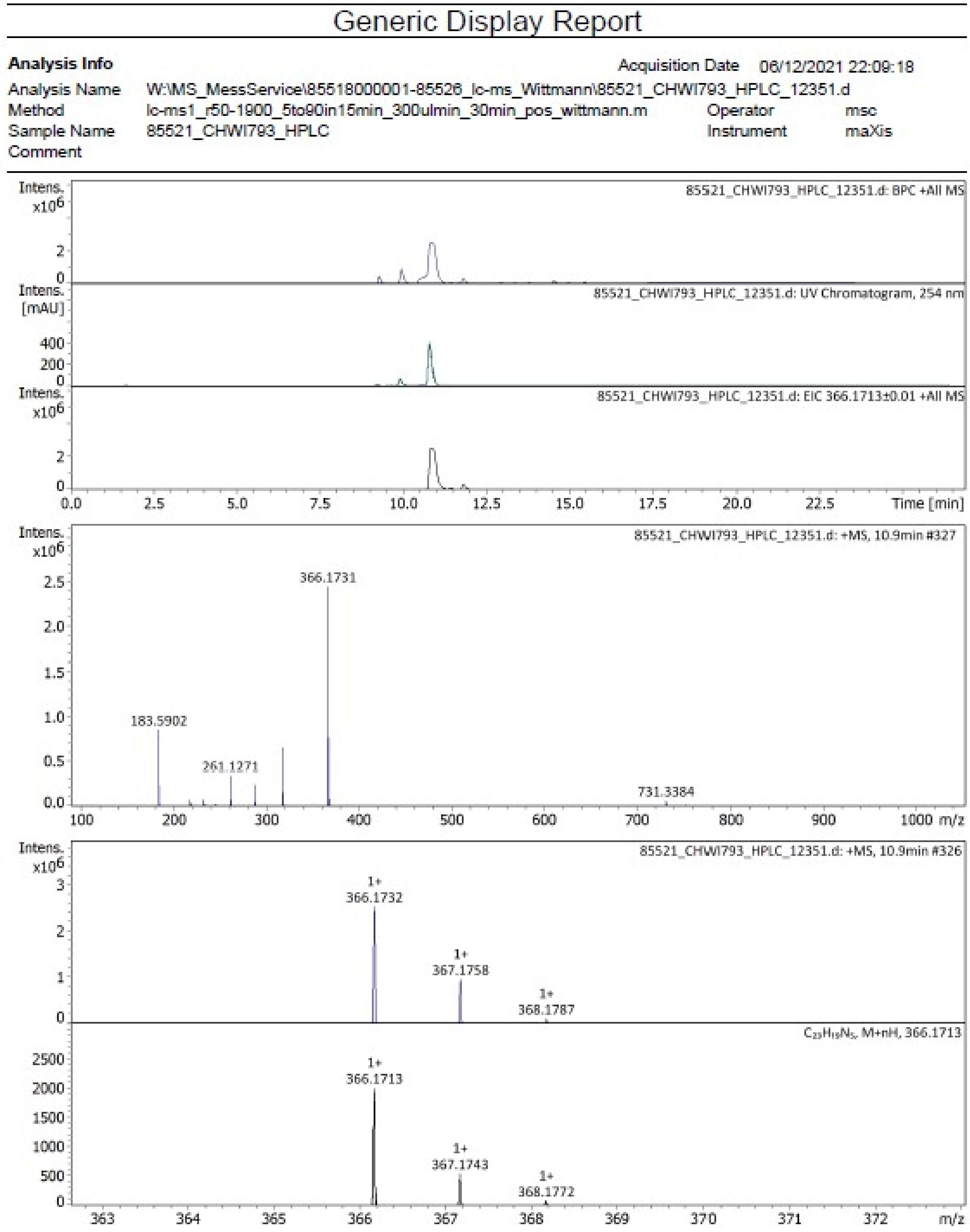

Figure S57. HPLC-HR-MS spectrum of **HL**[1] in positive ion mode.

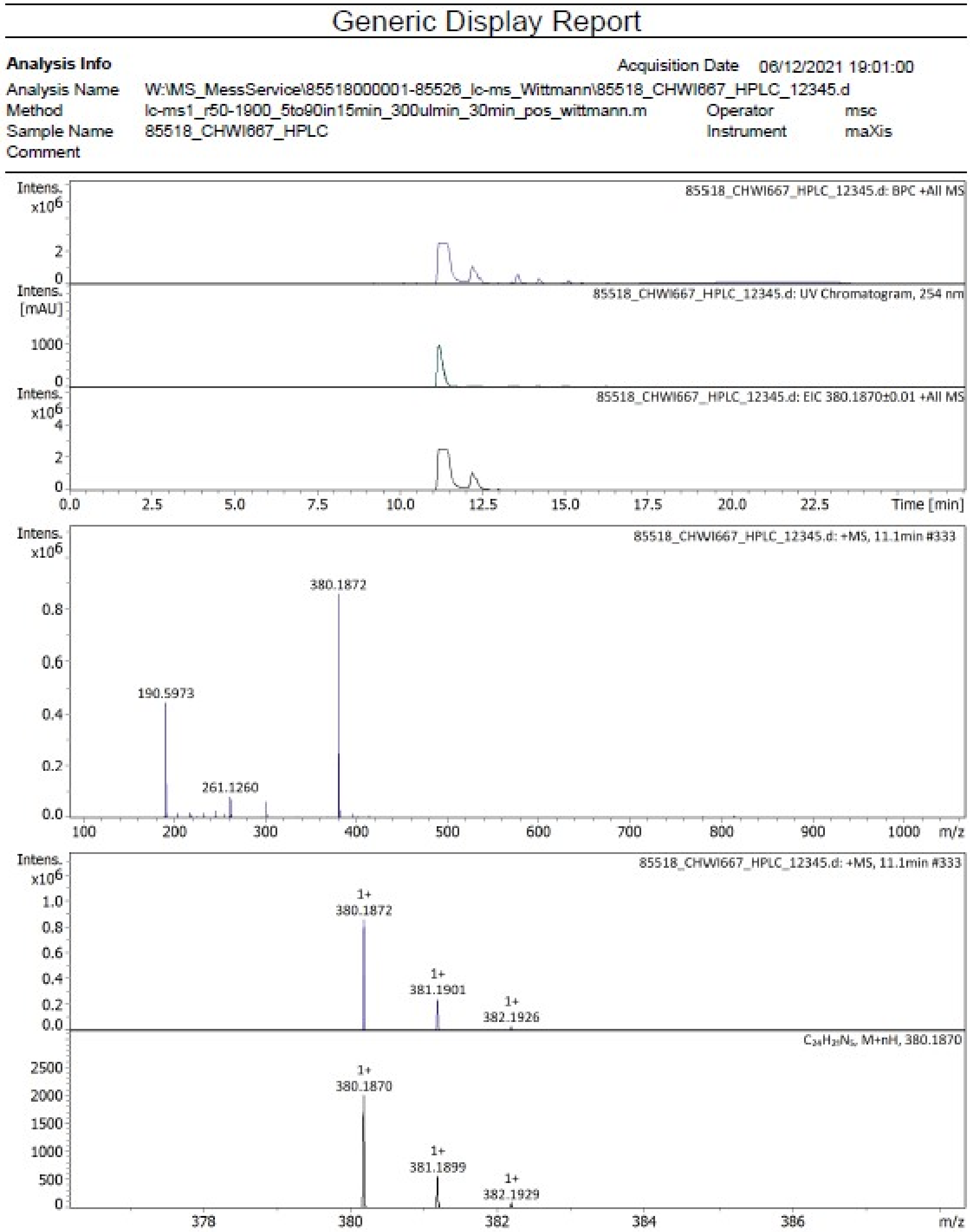

Figure S58. HPLC-HR-MS spectrum of **HL²** in positive ion mode.

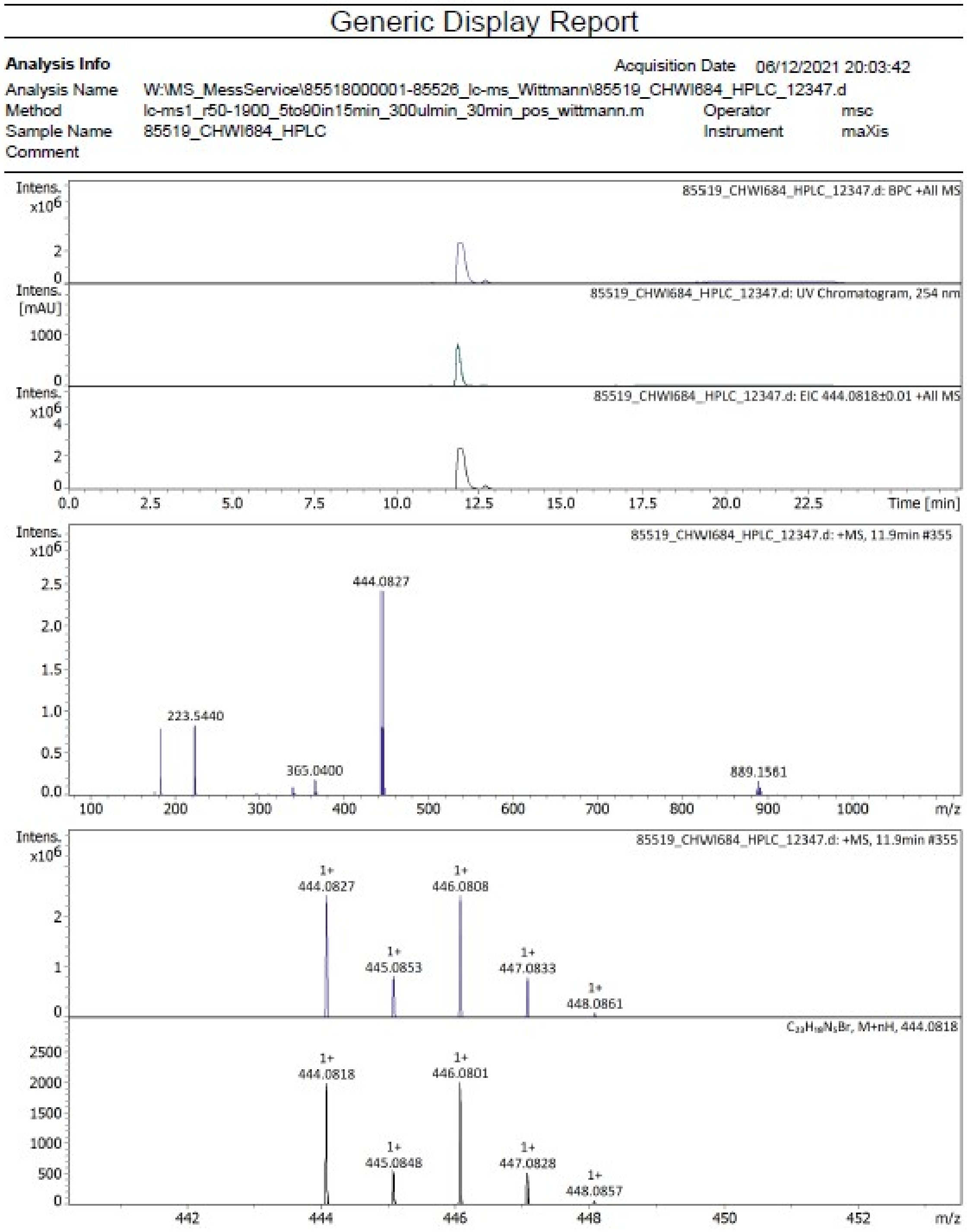

Figure S59. HPLC-HR-MS spectrum of **HL³** in positive ion mode.

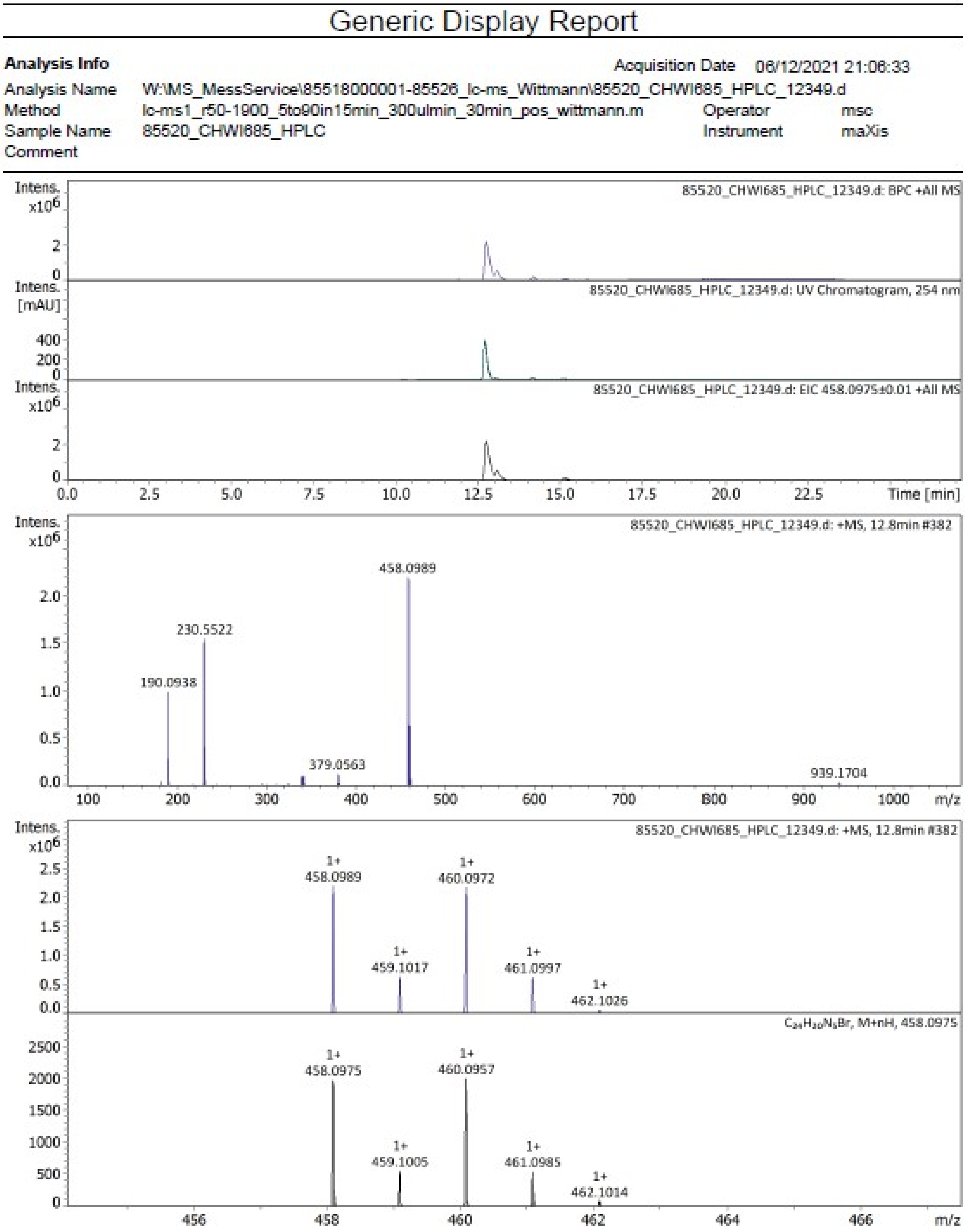

Figure S60. HPLC-HR-MS spectrum of **HL4** in positive ion mode.

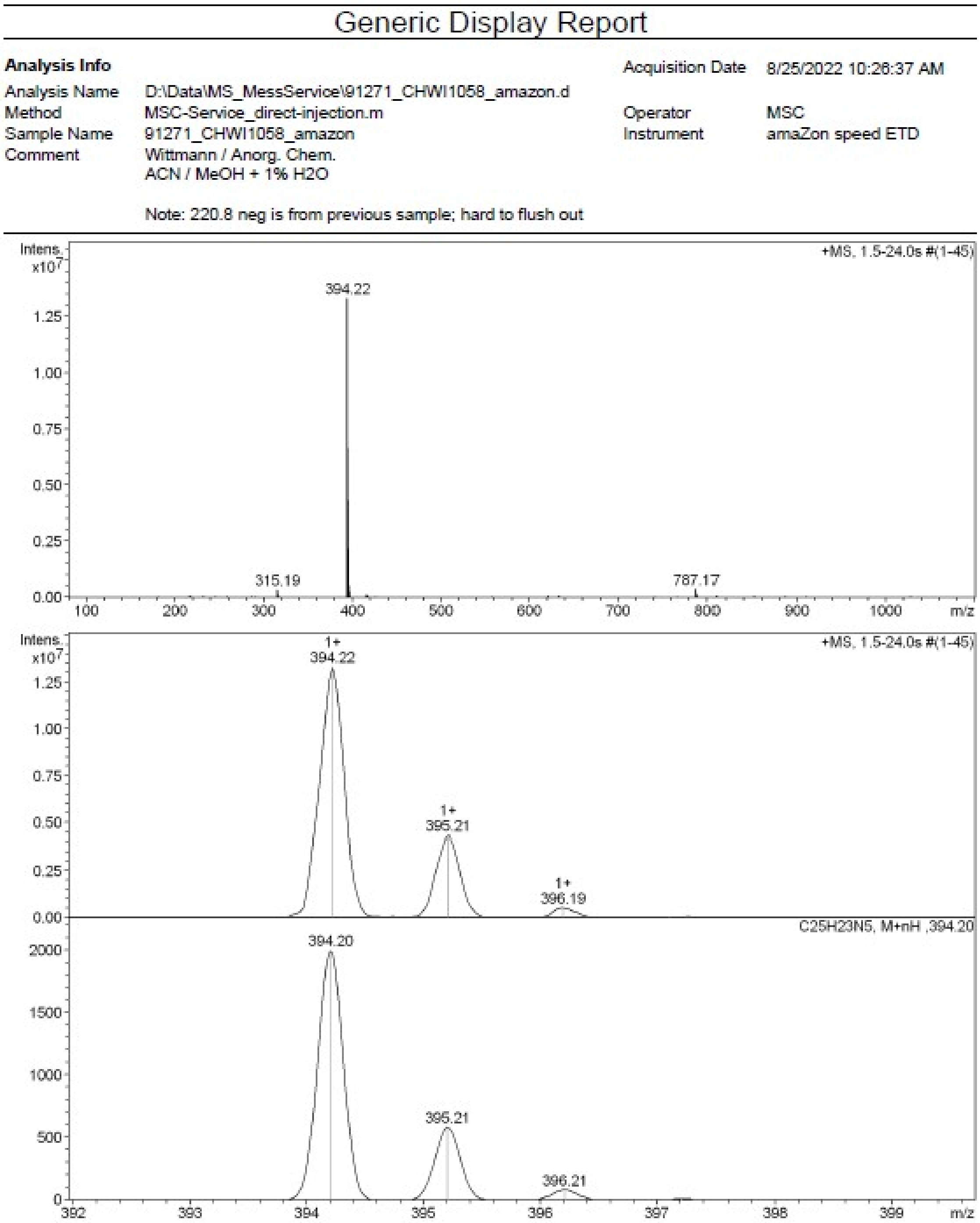

Figure S61. ESI mass spectrum of **HL^5** in positive ion mode.

Generic Display Report

Analysis Info		Acquisition Date	11/7/2022 11:33:09 AM
Analysis Name	D:\Data\MS_MessService\92795_RIUR 08_amazon.d		
Method	MSC-Service_direct-injection.m	Operator	MSC
Sample Name	92795_RIUR 08_amazon	Instrument	amaZon speed ETD
Comment	Wittmann / AOC<br>ACN / MeOH +1% H2O		

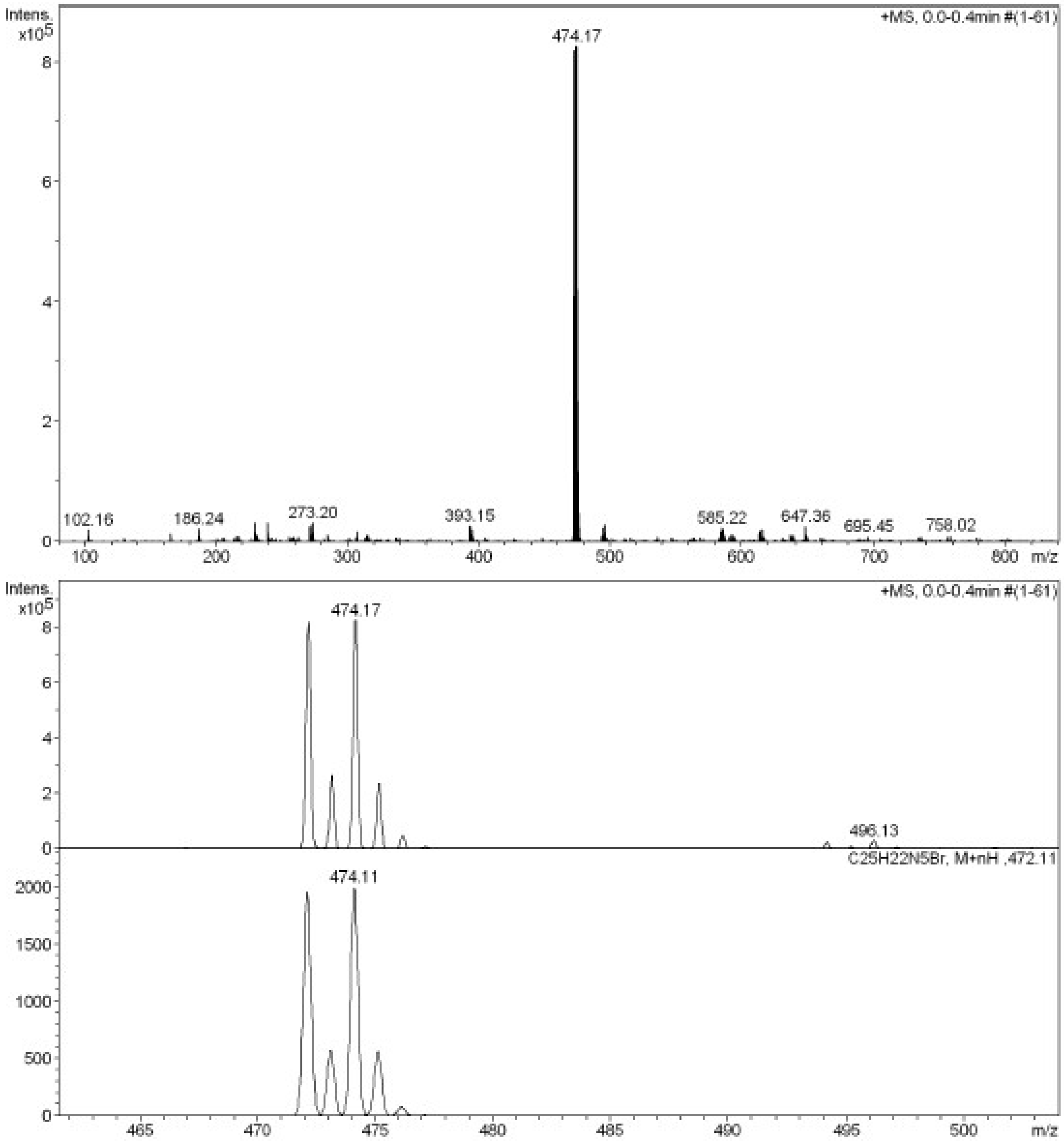

Figure S62. ESI mass spectrum of **HL⁶** in positive ion mode.

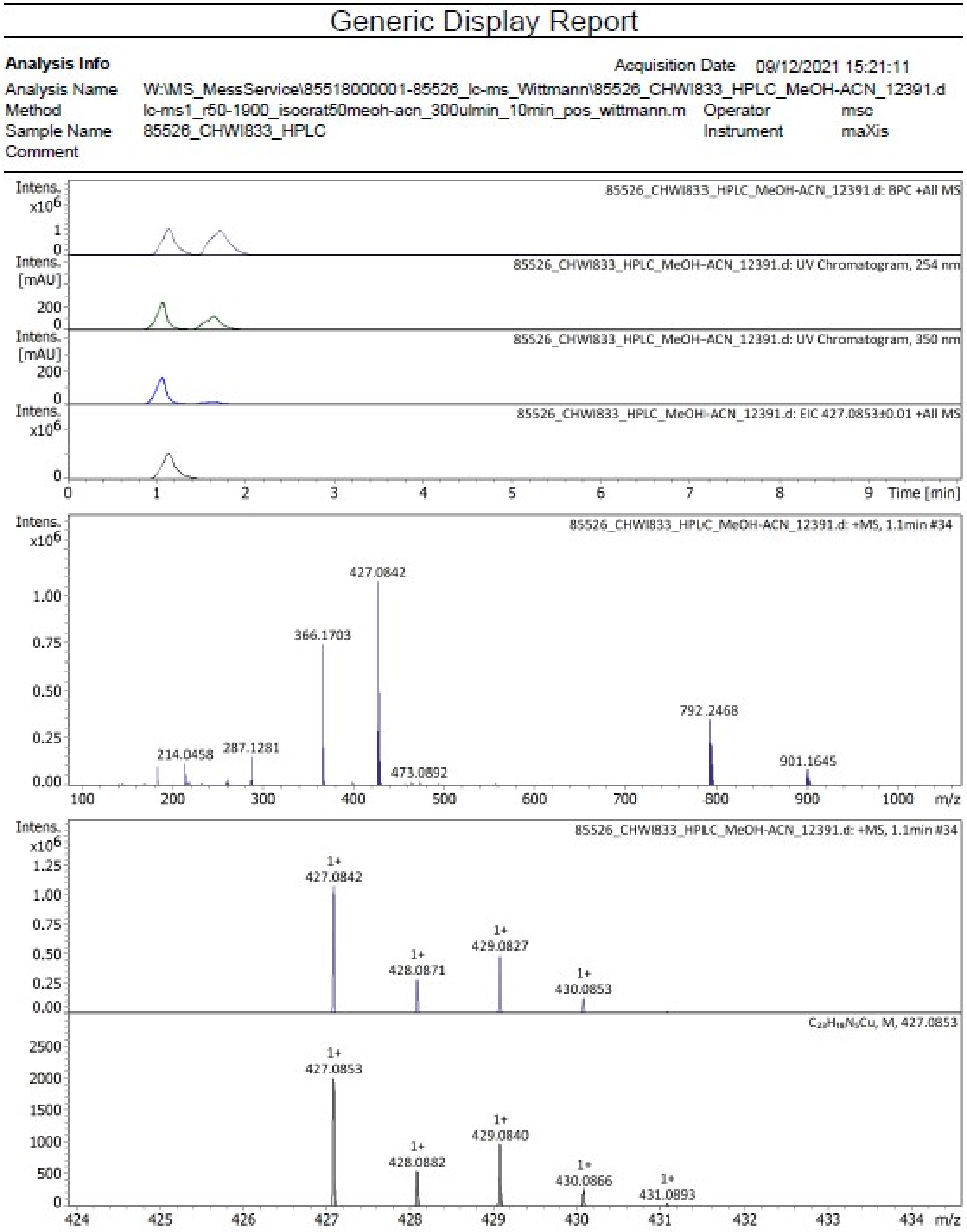

Figure S63. ESI mass spectrum of **1** in positive ion mode.

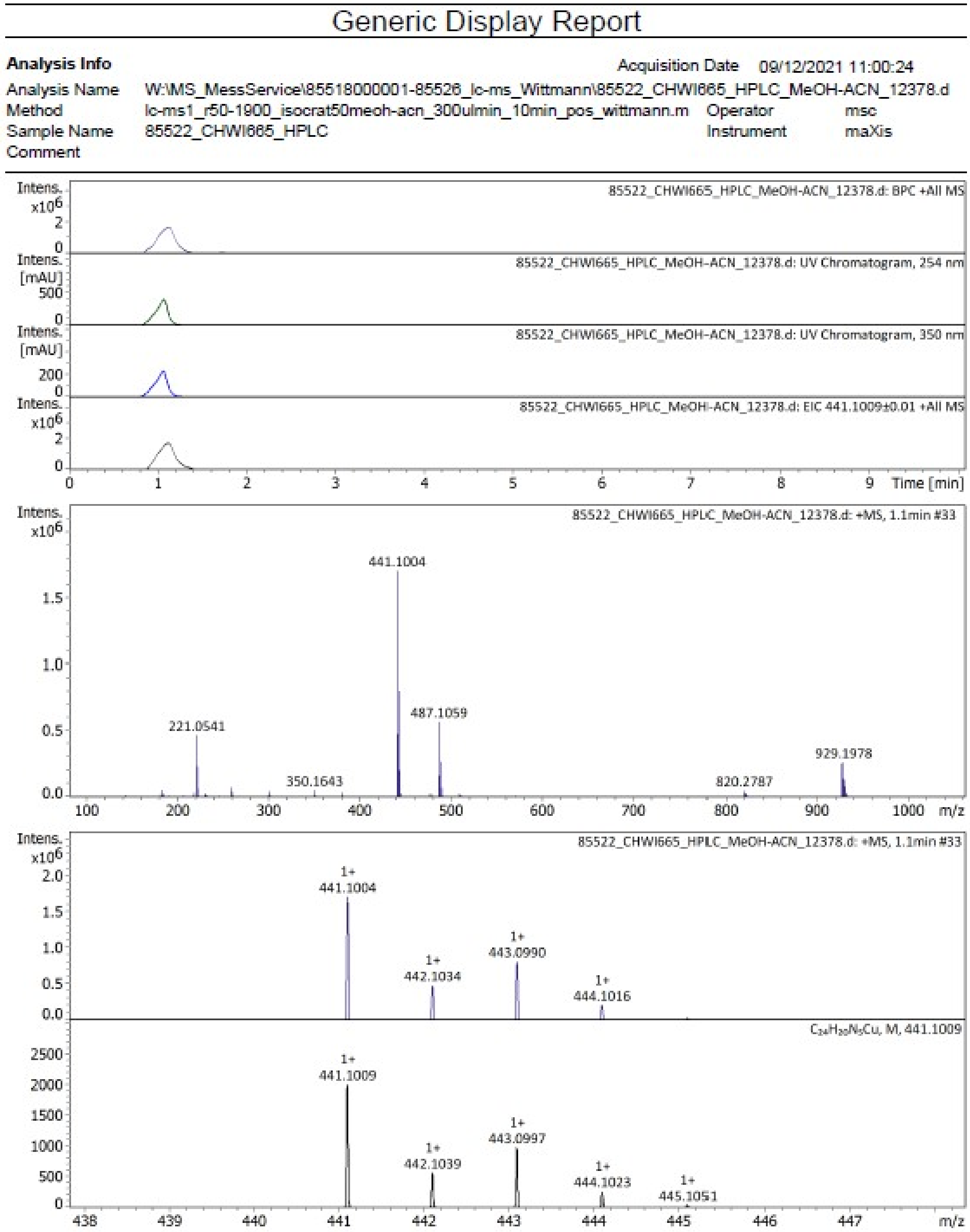

Figure S64. ESI mass spectrum of **2** in positive ion mode.

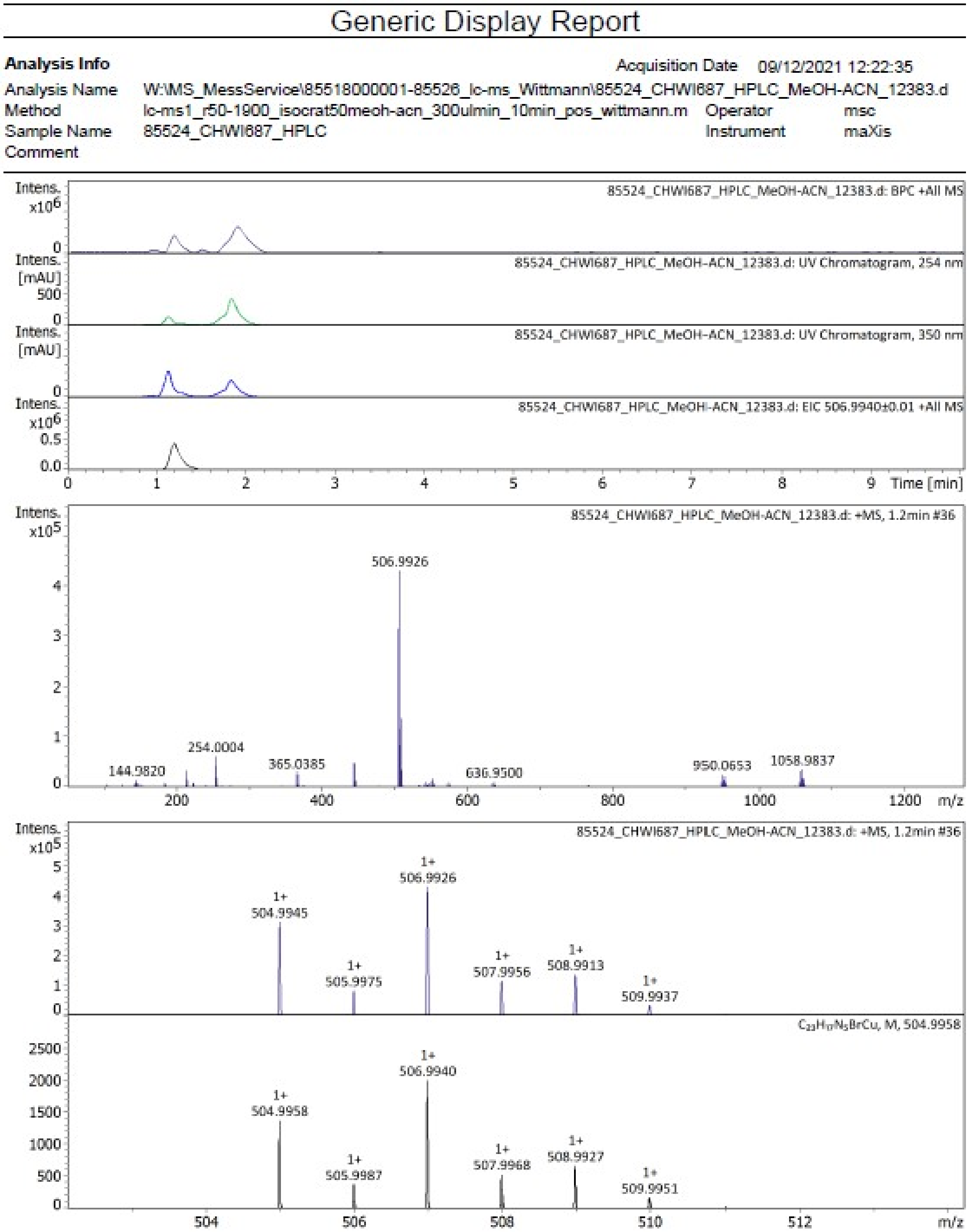

Figure S65. ESI mass spectrum of **3** in positive ion mode.

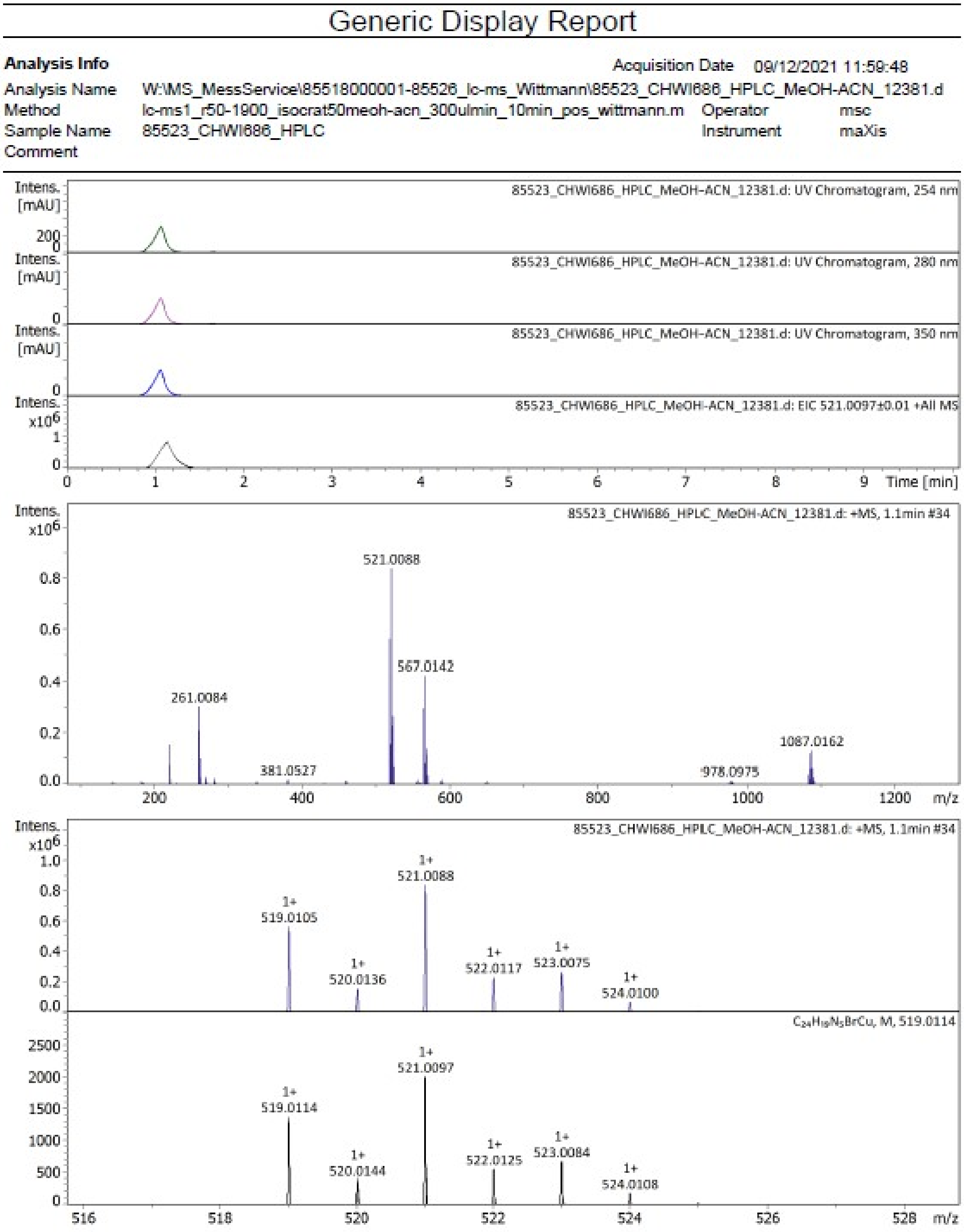

Figure S66. ESI mass spectrum of **4** in positive ion mode.

Generic Display Report

Analysis Info		Acquisition Date	11/9/2022 12:16:10 PM
Analysis Name	D:\Data\MS_Service_MSC_Archiv_2022_861_939\928\92885_CHWI 1059 pree_amazon2.d		
Method	MSC-Service_direct-injection.m	Operator	MSC
Sample Name	92885_CHWI 1059 pree_amazon2	Instrument	amaZon speed ETD
Comment	Wittmann / AOC<br>ACN / MeOH +1% H2O		

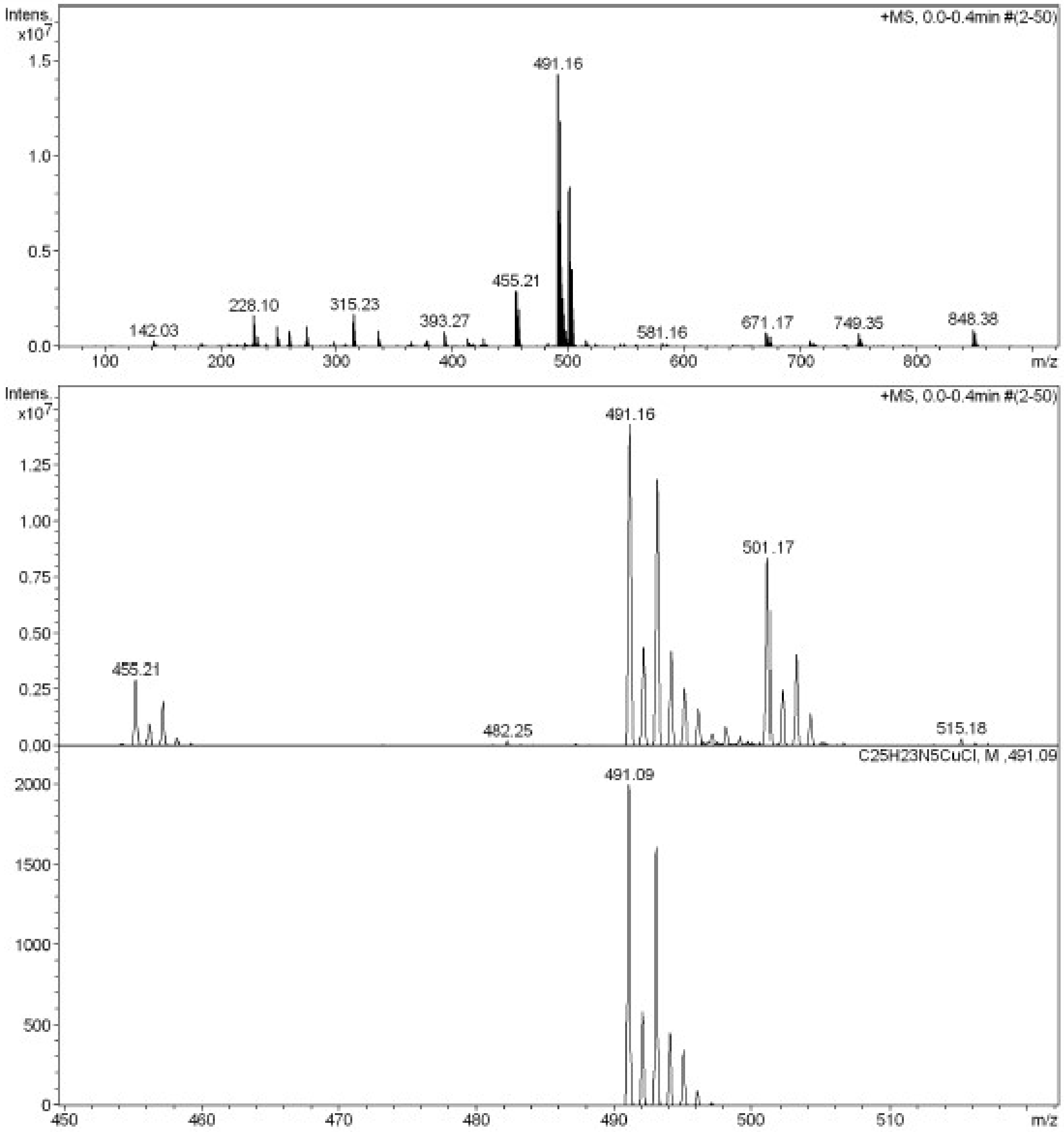

Figure S67. ESI mass spectrum of **5** in positive ion mode.

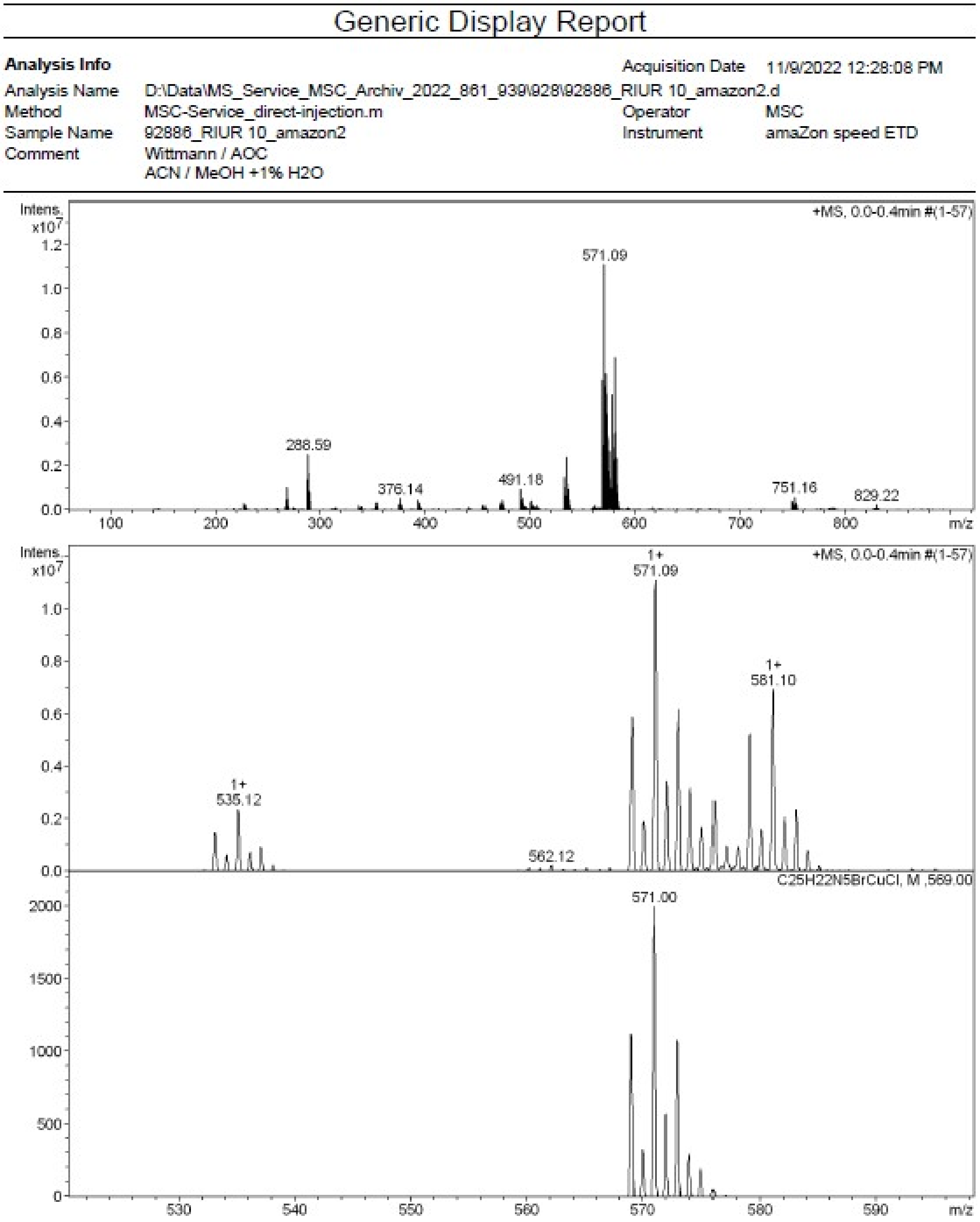

Figure S68. ESI mass spectrum of **6** in positive ion mode.

- **References**

1. Putey, A., Joucla, L., Picot, L., Besson, T. & Joseph, B. Synthesis of latonduine derivatives via intramolecular Heck reaction. *Tetrahedron* 2007, **63**, 867–879.

2. Ravelli, R. B. G. *et al.* Insight into tubulin regulation from a complex with colchicine and a stathmin-like domain. *Nature* 2004, **428**, 198–202.
